# Unveiling productivity: The interplay of cognitive arousal and expressive typing in remote work

**DOI:** 10.1371/journal.pone.0300786

**Published:** 2024-05-15

**Authors:** Samiul Alam, Saman Khazaei, Rose T. Faghih

**Affiliations:** 1 Department of ECE, University of Houston, Houston, Texas, United States of America; 2 Department of Biomedical Engineering, New York University, New York City, New York, United States of America; University of Bologna: Universita di Bologna, ITALY

## Abstract

Cognitive Arousal, frequently elicited by environmental stressors that exceed personal coping resources, manifests in measurable physiological markers, notably in galvanic skin responses. This effect is prominent in cognitive tasks such as composition, where fluctuations in these biomarkers correlate with individual expressiveness. It is crucial to understand the nexus between cognitive arousal and expressiveness. However, there has not been a concrete study that investigates this inter-relation concurrently. Addressing this, we introduce an innovative methodology for simultaneous monitoring of these elements. Our strategy employs Bayesian analysis in a multi-state filtering format to dissect psychomotor performance (captured through typing speed), galvanic skin response or skin conductance (SC), and heart rate variability (HRV). This integrative analysis facilitates the quantification of expressive behavior and arousal states. At the core, we deploy a state-space model connecting one latent psychological arousal condition to neural activities impacting sweating (inferred through SC responses) and another latent state to expressive behavior during typing. These states are concurrently evaluated with model parameters using an expectation-maximization algorithms approach. Assessments using both computer-simulated data and experimental data substantiate the validity of our approach. Outcomes display distinguishable latent state patterns in expressive typing and arousal across different computer software used in office management, offering profound implications for Human-Computer Interaction (HCI) and productivity analysis. This research marks a significant advancement in decoding human productivity dynamics, with extensive repercussions for optimizing performance in telecommuting scenarios.

## Introduction

The intricate relationship between stress and productivity has been a subject of extensive research across various disciplines [1–3]. Pioneering theories like the Yerkes-Dodson law [1] have proposed an inverted U-shaped function, suggesting an optimal level of stress that maximizes productivity. This law has profound implications for understanding the dynamics of workplace performance, individual differences in stress response, and the overall impact on mental and physical health. However, the universality and applicability of this law have been topics of debate, prompting further exploration into the nuanced relationship between stress and productivity across different contexts and populations.

Delving deeper into the neuroscientific perspective, stress regulation and productivity are intricately linked with specific brain regions and their interconnections. The amygdala, known for its role in processing emotions, especially fear and stress-related response, is a critical player in this domain. It interacts closely with the prefrontal cortex, which is central to executive functions, decision-making, and emotional regulation. The hippocampus, another key region, is vital for memory consolidation and is particularly sensitive to stress, which can impact its function and structure. These brain regions form a complex network that governs our responses to stress and influences cognitive capacities like attention, memory, and decision-making processes [4–6]. Understanding these neural mechanisms is crucial for comprehending how stress affects cognitive functions and, consequently, productivity.

In the context of this study, the physiological signals under investigation—such as autonomic nervous system (ANS) activations and HRV—are reflections of the underlying neural activities. The ANS, with its sympathetic and parasympathetic branches, is directly influenced by brain activity, particularly in regions like the amygdala and prefrontal cortex [7]. Heart rate variability, as a measure of ANS function, offers insights into the body’s stress response and its regulation by the central nervous system. Thus, exploring these physiological markers in the context of neural substrates provides a more comprehensive understanding of the stress-productivity nexus.

This study takes a closer look at the physiological dimensions of productivity, exploring parameters such as ANS activation, typing dynamics, and HRV. The assessment of ANS activations through EDA data via deconvolution techniques [8–22] provides valuable insights into the body’s arousal levels and stress response. HRV data, derived from ECG measurements [23], further complement these findings by offering a window into the heart’s response to stress, reflecting the dynamic balance between the sympathetic and parasympathetic nervous systems. Cognitive arousal, as influenced by these physiological factors, has significant implications for memory, attention, and overall cognitive performance [24, 25].

The investigation extends to typing dynamics, an innovative measure of cognitive state and productivity. The correlation between typing speed and brain activity levels [26] reveals how cognitive and emotional states can manifest in physical activity. Expressive writing, requiring significant emotional and cognitive engagement, serves as a critical area of study. This type of writing, often reflective and personal, can induce physiological changes correlating with heart rate and emotion [27, 28]. The typing patterns observed during such tasks offer a unique lens through which to view an individual’s cognitive and emotional state, providing a practical method to assess productivity [29].

A comprehensive approach that integrates different physiological signals enhances the accuracy of depicting arousal and emotion. Studies have shown that a combination of HRV, EDA, and typing dynamics can provide a more nuanced understanding of an individual’s cognitive state and emotional well-being [19, 30–47]. In our study, the simultaneous analysis of these signals aims to paint a comprehensive picture of the interplay between physiological responses and cognitive states, especially in the context of productivity and stress management.

The emergence of advanced IoT devices like the Empatica E4 and Emotibit [48, 49] has revolutionized the field of physiological measurement, allowing for high-frequency, non-intrusive data collection. These technological advancements facilitate deeper insights into the interaction between physiological signals and cognitive processes, opening new avenues for research in productivity, especially in remote work settings [50–53]. Additionally, these insights have the potential to inform the development of innovative closed-loop control systems for performance regulation and mental health monitoring [54–60].

In response to these advancements and the identified gaps in the literature, our study proposes a state-space approach to concurrently track cognitive arousal and expressive typing states, utilizing EDA, HRV, and typing dynamics as key indicators. This approach, grounded in control systems engineering and informed by Bayesian filtering techniques, enables us to model and monitor internal brain states and their physiological manifestations. Our methodology differs from previous works, which predominantly focused on either EDA or HRV for measuring cognitive arousal [10, 30, 33, 39, 41]. By integrating these diverse data streams, we aim to provide a more holistic view of the cognitive and emotional factors that influence productivity.

The inclusion of typing dynamics in our analysis is particularly novel, offering a tangible measure of cognitive engagement and expressive capacity. This aspect of the study is grounded in the understanding that cognitive processes are not only reflected internally but also externalized through physical behaviors like typing. By examining these dynamics, we can gain insights into the real-time cognitive states of individuals, especially in work environments where typing is a central activity.

Furthermore, the study aims to explore the role of stress in cognitive processes more deeply. Stress, while often perceived negatively, can also be a driving force for productivity when managed effectively. Understanding the optimal levels of stress, as suggested by the Yerkes-Dodson law, and their impact on different cognitive functions such as memory, attention, and problem-solving, is essential for this analysis. This exploration is particularly relevant in the context of remote work, where traditional stressors may be replaced or compounded by new challenges related to isolation, work-life balance, and technological reliance.

With the increasing prevalence of remote work, understanding the interplay between physiological responses, cognitive states, and productivity has taken on new significance. Our study aims to contribute valuable insights into how remote workers can optimize their performance by managing stress and leveraging their cognitive and emotional states. The application of our findings could lead to the development of tools and strategies to enhance productivity and well-being in remote work settings. Moreover, the potential for integrating these insights into the design of ergonomic workspaces, productivity software, and mental health support systems is vast.

Our methodology involves detailed data processing and employs cutting-edge technologies and sophisticated Bayesian models. The findings from this research are expected to not only validate existing theories but also reveal new patterns and insights into the relationship between stress, cognitive arousal, and productivity. We will present results from both simulated and experimental data, offering a robust evaluation of our approach.

Finally, the discussion and conclusion sections of this manuscript will reflect on the implications of our findings, considering both their theoretical and practical applications. We will also outline potential future research directions, emphasizing the importance of continued exploration in this field to fully harness the benefits of understanding the stress-productivity relationship in modern work environments.

## Methods

In this section, we delineate the methodologies employed in our study. We describe the dataset [61] that we used, emphasizing the multimodal nature of the dataset which includes physiological signals, computer logging, and behavioral observations under varying stress conditions. The heart of our methodological framework lies in the application of a state-space model to capture the latent dynamics of cognitive arousal and expressive typing state, as represented by physiological and psychomotor parameters. This model is complemented by a multi-state filtering algorithm, following an Expectation-Maximization (EM) structure, to robustly estimate the system states and parameters [53, 62]. The algorithm integrates both forward and backward data [34, 40], refining the state space and parameter estimates to enhance the accuracy and reliability of our findings. Through these sophisticated statistical techniques, we aim to offer a comprehensive understanding of the interplay between cognitive arousal, stress, and physiological responses within the context of knowledge work.

### Data

In this study, we utilize the SWELL Knowledge Work (SWELL-KW) dataset [61], which is particularly significant due to its comprehensive collection of multimodal data like computer logging, facial expression from camera recordings, body posture from a Kinect 3D sensor, as well as physiological signals like heart rate and skin conductance obtained from body sensors. The dataset, encompassing recordings from 25 subjects performing typical desk work tasks under varied conditions, provides a rich source for analyzing the complex interactions between physiological states and knowledge work activities. The subjects were exposed to different stressors, such as email interruptions and time constraints, making it an ideal context for exploring the relationship between cognitive load, stress, and physiological responses. Despite its robustness, the dataset does have limitations, including the relatively small sample size and the controlled experimental setup, which might not fully capture the complexity of real-world work settings. In our study, we focus specifically on EDA and HRV to assess stress and cognitive load, complemented by detailed analysis of computer logs for mouse and keyboard activities to measure the expressive typing state. The participants’ subjective experiences, assessed through validated questionnaires, add qualitative depth to our multi-faceted analysis, providing a holistic view of the impact of stressors in knowledge work.

### State-space model

Assume that our state vector **x**_*k*_ involves two states (cognitive arousal state and expressive typing state) and evolves with time following

$$\begin{array}{c} x_{k}=x_{k-1}+e_{k}. \end{array}$$ (1)

where $x_{k}={\left[x_{k,1}\;x_{k,2}\right]}^{⊺}$, $e_{k}∼N\left(0,\Sigma \right).$ We observe two sets of binary variables. These are the deconvolved skin conductance impulse events *n*_*k*,1_ and the key press events *n*_*k*,2_ which we consider as Bernoulli-distributed random variables with mass functions $p_{k,1}^{n_{k,1}}{\left(1-p_{k,1}\right)}^{1-n_{k,1}}$ and $p_{k,2}^{n_{k,2}}{\left(1-p_{k,2}\right)}^{1-n_{k,2}}$, respectively. Here *p*_*k*,1_ = *P*(*n*_*k*,1_ = 1) and *p*_*k*,2_ = *P*(*n*_*k*,2_ = 1). These variables are related to **x**_*k*_ as

$$\begin{array}{c} p_{k,m}=\frac{1}{1+e^{-(\beta_{m}+x_{k,m})}}, \end{array}$$ (2)

where *m* ∈ 1, 2 and *β*_*m*_ are constant coefficients to be determined and *x*_*k*,1_ and *x*_*k*,2_ are independent random variables. We determine $B=[\beta_{1},\beta_{2}]$ empirically similar to [44] assuming that *x*_0,*m*_ ≈ 0 ∀*j* at the very beginning of the random walk. Therefore,

$$\begin{array}{c} \beta_{m}=\text{log}(\frac{p_{0,m}}{1-p_{0,m}}). \end{array}$$ (3)

We use HRV as our continuous variable *r*_*k*_ which we assume is related to ***x***_*k*_ linearly as

$$\begin{array}{c} r_{k}=\gamma_{0}+\gamma_{1}x_{k,1}+\gamma_{2,k}x_{k,2}+v_{k}, \end{array}$$ (4)

where $v_{k}∼N\left(0,\sigma_{v}^{2}\right)$ is sensor noise and $\gamma =[\begin{array}{ccc} \gamma_{0} & \gamma_{1} & \gamma_{2} \end{array}]$ are parameters relating **r_k_** with the hidden state variable **x**_k_.

### Multi-state filter algorithm overview

The multi-state filter algorithm is based on the Expectation-Maximization (EM) approach, used for estimating parameters in a statistical model. The algorithm involves iterative calculations over two main steps: the E-step (Expectation) and the M-step (Maximization).

#### E-Step

The algorithm begins by approximating the posterior density as a Gaussian distribution following [62]. The filter update process involves solving for the state estimate ***x***_*k*|*k*_, which results in the following update rules:

$$\begin{array}{cc} & x_{k|k}=x_{k|k-1}+W_{k|k-1}\frac{\partial f(x_{k})}{\partial x_{k}}{|}_{x_{k|k}}, \end{array}$$ (5)

$$\begin{array}{c} W_{k|k}={\left(W_{k|k-1}^{-1}-{\frac{\partial^{2}f\left(x_{k}\right)}{\partial x_{k}^{2}}|}_{x_{k|k}}\right)}^{-1}, \end{array}$$ (6)

#### Backward smoother equations

For improving state space estimates by using both backward and forward data, the following backward smoothing update equations are utilized:

$$\begin{array}{c} A_{k}=W_{k|k}W_{k+1|k}^{-1} \end{array}$$ (7)

$$\begin{array}{c} x_{k|K}=x_{k|k}+A_{k}(x_{k+1|K}-x_{k+1|k}) \end{array}$$ (8)

$$\begin{array}{c} W_{k|K}=W_{k|k}+A_{k}^{2}(W_{k+1|K}-W_{k+1|k}). \end{array}$$ (9)

#### M-step derivations

This next step focuses on updating the model parameters based on the expected log-likelihood. The updates for *γ*_0_, *γ*_1_ and *γ*_2_ can be derived by solving for the following system of linear equations:

$$\begin{array}{rrr} \sum_{k=1}^{K}r_{k}-K\gamma_{0}-\gamma_{1}\sum_{k=1}^{K}x_{k|K,1} & -\gamma_{2}\sum_{k=1}^{K}x_{k|K,2} & =0\\ \sum_{k=1}^{K}r_{k}x_{k|K,1}-\gamma_{0}\sum_{k=1}^{K}x_{k|K,1} & -\gamma_{1}\sum_{k=1}^{K}u_{k|K,1} & \;\\ \; & -\gamma_{2}\sum_{k=1}^{K}x_{k|K,1}x_{k|K,2} & =0\\ \sum_{k=1}^{K}r_{k}x_{k|K,2}-\gamma_{0}\sum_{k=1}^{K}x_{k|K,2} & -\gamma_{1}\sum_{k=1}^{K}x_{k|K,1}x_{k|K,2} & \;\\ \; & -\gamma_{2}\sum_{k=1}^{K}u_{k|K,2} & =0. \end{array}$$ (10)

The updates for $\sigma_{v}^{2}$, *σ*_*ϵ*,1_ and *σ*_*ϵ*,2_ are as follows:

$$\begin{array}{c} \sigma_{v}^{2}=\frac{1}{K}\{\sum_{k=1}^{K}r_{k}^{2}+K\gamma_{0}^{2}+\gamma_{1}^{2}\sum_{k=1}^{K}u_{k|K,1}+\gamma_{2}^{2}\sum_{k=1}^{K}u_{k|K,2}\\ \;-2\gamma_{0}\sum_{k=1}^{K}r_{k}-2\sum_{k=1}^{K}r_{k}x_{k|K,1}-2\sum_{k=1}^{K}r_{k}x_{k|K,2}\\ \;+2\gamma_{0}\sum_{k=1}^{K}x_{k|K,1}+2\gamma_{0}\sum_{k=1}^{K}x_{k|K,2}+\sum_{k=1}^{K}x_{k|K,1}x_{k|K,2}\}. \end{array}$$ (11)

$$\begin{array}{c} \sigma_{\epsilon ,1}^{2}=\frac{2}{K}\{\sum_{k=1}^{K}u_{k|K,1}-2\sum_{k=1}^{K}v_{k|K,1}+\sum_{k=1}^{K}u_{k-1|K,1}\}\\ \sigma_{\epsilon ,2}^{2}=\frac{2}{K}\{\sum_{k=1}^{K}u_{k|K,2}-2\sum_{k=1}^{K}v_{k|K,2}+\sum_{k=1}^{K}u_{k-1|K,2}\}. \end{array}$$ (12)

The full derivations for these equations can be found in the S1 Appendix. Following this steps the algorithm executes iteratively, alternating between the E-Step and M-Step for a set number of iterations. Each iteration refines the parameter estimates, thereby improving the model’s fit to the observed data.

An overview of the algorithm is shown in Algorithm 1

**Algorithm 1** Overview of the Multi-State Filter Algorithm

Initialize Variables

**for** N iterations **do**

 *x*_1_ ← 0

 *W*_1_ ← *σ*_*e*_

 **for** j from 2 to J **do**      ▷ E − Step

  *X*_*j*_ ← *X*_*j*−1_

  Solve $x_{k|k}=x_{k|k-1}+W_{k|k-1}\frac{\partial f(x_{k})}{\partial x_{k}}{|}_{x_{k|k}}$ for *X*_*j*|*j*−1_ from Eq (5)

  $W_{k|k}={\left(W_{k|k-1}^{-1}-{\frac{\partial^{2}f\left(x_{k}\right)}{\partial x_{k}^{2}}|}_{x_{k|k}}\right)}^{-1}$

 **end for**      ▷ Smoothing

 $A_{k}=W_{k|k}W_{k+1|k}^{-1}$

 ***x***_*k*|*K*_ = ***x***_*k*|*k*_ + ***A***_*k*_(***x***_*k*+1|*K*_ − ***x***_*k*+1|*k*_)

 $W_{k|K}=W_{k|k}+A_{k}^{2}(W_{k+1|K}-W_{k+1|k})$      ▷ M Step

 Update *γ*[0, 1, 2] via Eq (10)

 Update *σ*_*v*_ via Eq (11)

 Update *σ*_*ϵ*,[1,2]_ via Eq (12)

**end for**

## Results and discussion

### Evaluation using simulated data

**Fig 1:**
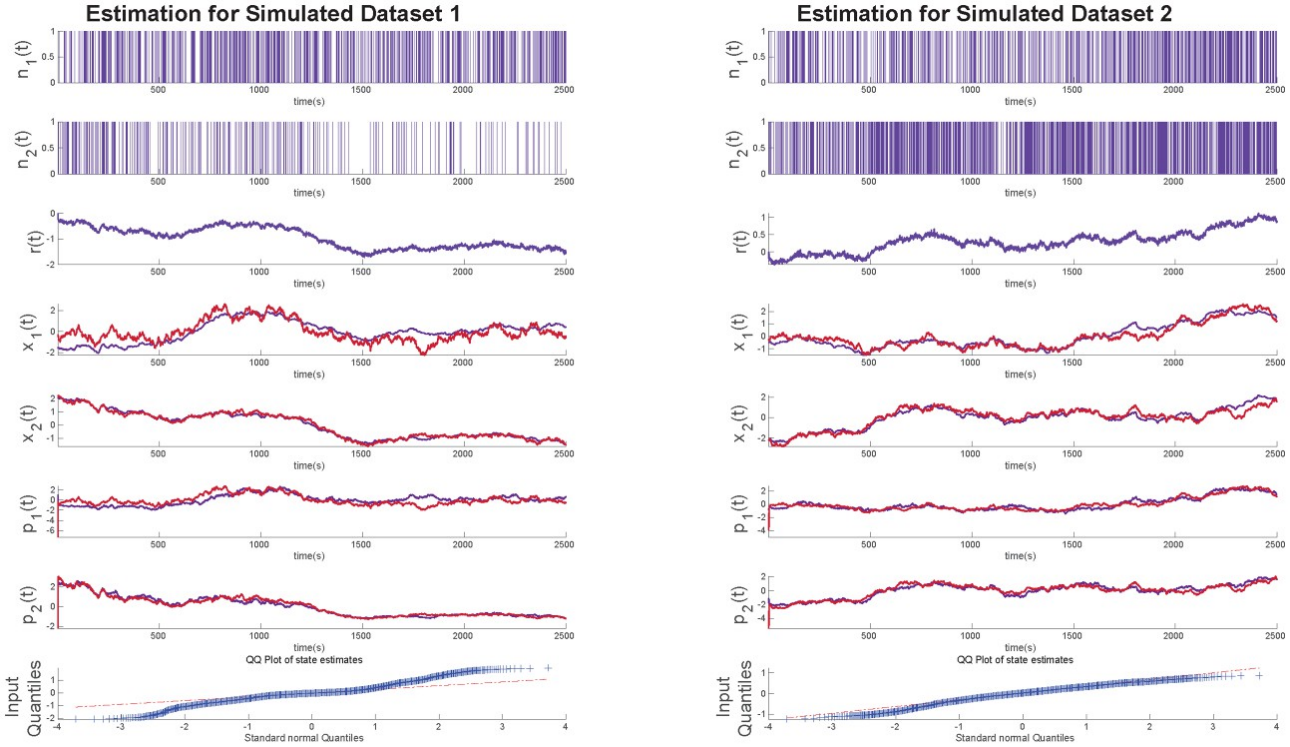
Latent state estimations for simulated data. The two panels above in order from top show the simulated binary random variables *n*_1_ and *n*_2_, the continuous variable *r*, the estimated latent variables ${\tilde{x}}_{1}$ and ${\tilde{x}}_{2}$ (shown in blue) alongside the ground truths *x*_1_ and *x*_2_ (shown in red). Following that are the occurrence probabilities ${\tilde{p}}_{1}$ and ${\tilde{p}}_{2}$ (shown in blue) alongside the ground truths *p*_1_ and *p*_2_ (shown in red). Lastly, the quantile-quantile (QQ) plot for the residual error of *x* is shown.

To validate the multi-state filter, we compare its accuracy on simulated datasets. We generated ten sets of simulated data. To generate the data we randomly assigned typical values of $\gamma =[\begin{array}{ccc} \gamma_{0} & \gamma_{1} & \gamma_{2} \end{array}]$ such that they are in range [−0.7, 0.7]. We also assigned *σ*_*e*_ = [0.003, 0.003] and *σ*_*v*_ = 0.03. We created a random vector $\epsilon =N(0,\sigma_{e}^{2})$ of length *K* = 2500 and generated the state values *x* according to Eq (1). We then calculated the value of vector **r** from Eq (4). Following this, we calculate B as shown in Eq (3) based on approximating *p*_0,1_ and *p*_0,2_ by the average probability of an impulse occurring in the data similar to [44]. We simulated two point processes having a true value of *p*_0,1_ = 0.2 and *p*_0,2_ = 0.3. Using these generated data, we tested our algorithm’s capacity to estimate the unobserved sympathetic arousal and expressive typing states and recover the model parameters from the set of observations. The results of this simulation for 2 datasets are shown in Fig 1. The QQ plots indicate that we obtain good fits to the simulated data. The state estimate can deviate from the true value in regions where there are large gaps between impulses for an extended period of time as we see for the 1st simulated data in the left panel. The estimates are closer to the true state in regions where more impulses tend to occur.

We estimate observed system parameters for 10 different simulated datasets and the percentage deviations from the true values are shown in Table 1. In the table, we see that, *γ*_0_, *γ*_2_, and *σ*_*v*_ show relatively smaller errors overall. It is important to note that these system parameters can have multiple solutions due to the greater degrees of freedom afforded by multiple states. Therefore, the technique may not always converge to the exact values with which the simulated data were created.

**Table 1:** Estimation Error of system parameters for our approach on simulated data.

System Parameter	Average Percentage Error
*γ*_0_	2.85
*γ*_1_	11.4
*γ*_2_	2.95
*σ*_*v*_	5.00
$\sigma_{e_{1}}$	8.6
$\sigma_{e_{2}}$	9.9

### Experimental results and discussions

**Fig 2:**
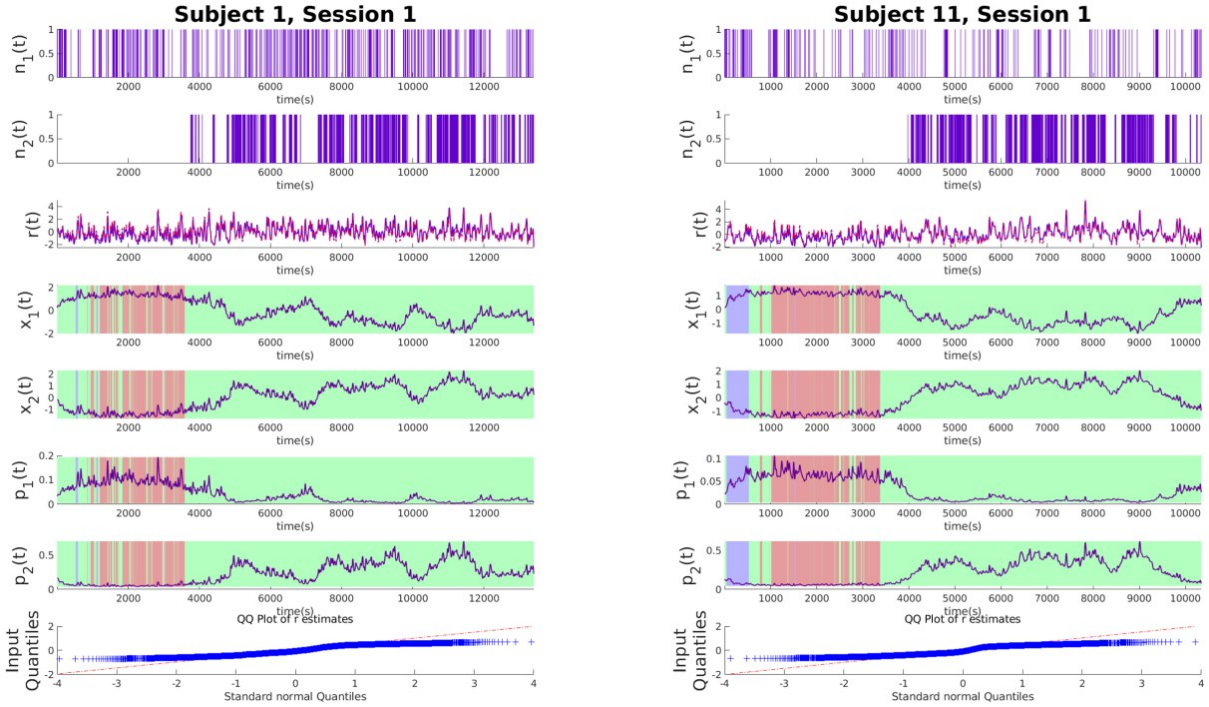
Latent state estimations for experimental data with no stressors. Each of the panels above shows the experimental data for no-stressor sessions. From the top, the binary variables *n*_1_ and *n*_2_ derived from deconvolved EDA data and typing data respectively, the continuous variable *r* denoting the RR intervals derived from heart rate (red line) and $\tilde{r}$ estimated *x*_1_ and *x*_2_ (purple line) *x*_1_ and *x*_2_ in order from top indicating cognitive arousal state and expressive typing state respectively. *p*_1_ and *p*_2_ show the estimated probabilities. Patches of green, red, and cyan indicate what application the subject was using at the time of measurement. Green indicates applications for information search like internet explorer, red is for typing like Microsoft word and PowerPoint, and cyan is for when subjects are looking at their emails. Finally, the QQ plot for the residual error of *r* is shown. The panel on the left is for subject 1 and the one on the right is for subject 11.

**Fig 3:**
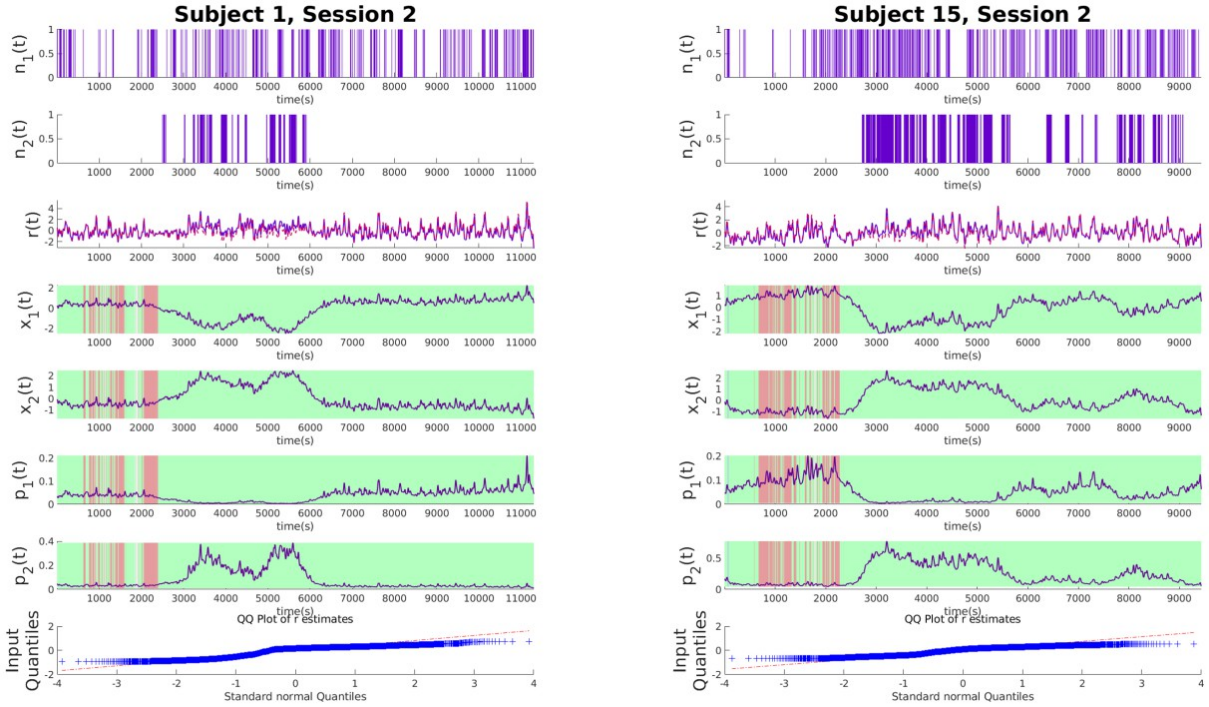
Latent state estimations for experimental data with time limit. Each of the panels above shows the experimental data for no-stressor sessions. From the top, the binary variables *n*_1_ and *n*_2_ derived from deconvolved EDA data and typing data respectively, the continuous variable *r* denoting the RR intervals derived from heart rate (red line) and $\tilde{r}$ estimated *x*_1_ and *x*_2_ (purple line), *x*_1_ and *x*_2_ in order from top indicating cognitive arousal state and expressive typing state respectively. *p*_1_ and *p*_2_ show the estimated probabilities. Patches of green, red, and cyan indicate what application the subject was using at the time of measurement. Green indicates applications for information search like internet explorer, red is for typing like Microsoft word and PowerPoint, and cyan is for when subjects are looking at their emails. Finally, the QQ plot for the residual error of *r* is shown. The panel on the left is for subject 1 and the one on the right is for subject 15.

**Fig 4:**
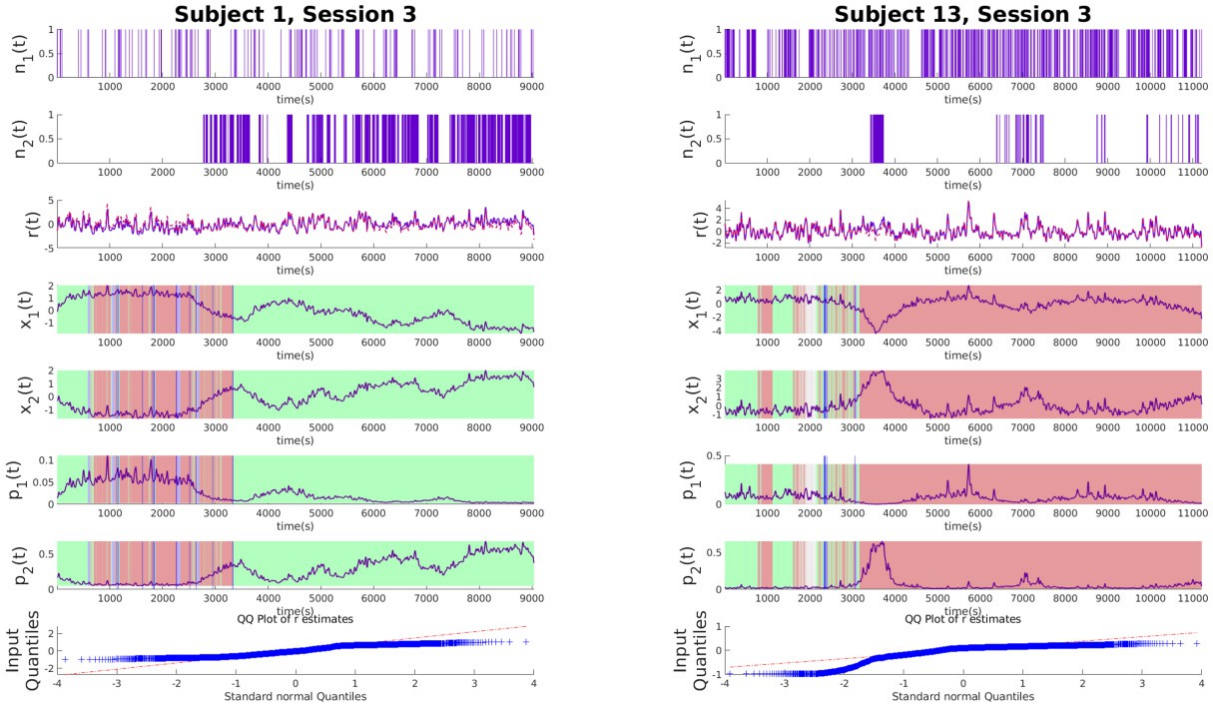
Latent state estimations for experimental data with interruptions. Each of the panels above shows the experimental data for no-stressor sessions. From the top, the binary variables *n*_1_ and *n*_2_ derived from deconvolved EDA data and typing data respectively, the continuous variable *r* denoting the RR intervals derived from heart rate (red line) and $\tilde{r}$ estimated from latent variables *x*_1_ and *x*_2_ (purple line), *x*_1_ and *x*_2_ in order from top indicating cognitive arousal state and expressive typing state respectively. *p*_1_ and *p*_2_ show the estimated probabilities. Patches of green, red, and cyan indicate what application the subject was using at the time of measurement. Green indicates applications for information search like internet explorer, red is for typing like Microsoft word and PowerPoint, and cyan is for when subjects are looking at their emails. The Blue vertical line indicates the time email notifications were sent. Finally, the QQ plot for the residual error of *r* is shown. The panel on the left is for subject 1 and the one on the right is for subject 13.

To illustrate the effectiveness of the algorithm, we analyze its performance on experimental data from the SWELL-KW dataset. In the experiment, the subject is tasked with writing a general essay under different conditions as explained in the Data section. Figs 2–4 show examples of decoded arousal and expressive typing states for each session. We can expect the subject to feel more cognitive stress initially when they start searching for resources online, which gradually decreases once the subject feels more confident about the topic. Conversely, after gaining a good handle on the topic, the expressive typing state will also increase after some time as the stress decreases. Figs 2–4 also indicate that this trend is common for all sessions and is appropriately captured by the algorithm.

It is essential to note that unlike in [34] where the absence of neural stimuli caused the EM estimate to fail in predicting minute changes in the latent state, here, the algorithm does not suffer from that problem. This is due to the fact that the algorithm uses multiple binary random variables to predict the latent states and it will only fail when both impulses become scarcer which we see exemplified in Fig 1.

### Influence of stressors

In our analysis of the SWELL dataset, where three distinct sessions were recorded for each experiment, we observe significant behavioral patterns emerging across different stress conditions. Each session involved subjects writing essays, a task requiring both information gathering and opinion expression. The first session serves as a baseline with no stressors, providing a control scenario for comparison. The second session introduces a time constraint, adding a layer of urgency and thereby testing the subjects’ ability to manage time pressure. In the final session, subjects face frequent email interruptions, challenging their focus and multitasking abilities. These varying conditions, analyzed in Figs 2–4, reveal clear patterns in cognitive and emotional responses to stressors. Notably, the hidden states captured in the data effectively track the arousal and expressive typing states across different sessions. This is evident when correlating the active applications used by the subjects with the respective state values. The significance of these findings lies in their potential to enhance our understanding of stress impacts in knowledge work environments. By analyzing how subjects adapt their cognitive and emotional responses under varying stress conditions, we can gain insights into designing more effective work strategies and tools, particularly in scenarios replicating real-world challenges such as time constraints and frequent interruptions.

#### Baseline session

For the first session or the baseline session, where no external stressors were introduced, subjects exhibited a unique pattern of cognitive and expressive behaviors, as seen in Fig 2 for subjects 1 and 11. This session was characterized by a more relaxed environment, allowing subjects to engage in the essay-writing task with relative ease. Initially, the subjects focused on gathering information, which is reflected in the elevated cognitive arousal state, denoted as *x*_1_. This heightened state of arousal during the information gathering phase indicates an intensive cognitive engagement, a critical aspect of the knowledge work. As the session progressed and the subjects shifted from information gathering to writing, there was a noticeable transition in their cognitive state. The arousal state gradually stabilized or oscillated, indicating a shift in cognitive demands. Concurrently, the expressive typing state, *x*_2_, which initially started at a lower level, began to rise. This increase in *x*_2_ aligns with the subjects’ transition into the writing phase, where expressive typing becomes more pronounced. The elevated expressive typing state towards the latter part of the session underscores the subjects’ engagement in the creative and expressive aspect of essay writing. This observation from the baseline session provides valuable insights into the natural progression of cognitive and expressive states in a stress-free environment, serving as a crucial reference point for comparing subject behaviors under different stress conditions in subsequent sessions.

#### Timed sessions

In the timed sessions of our study, while the general patterns of expressive typing state and cognitive arousal observed across all sessions align with the trends described in Experimental Results And Discussions, a distinct dynamic emerges under the time constraints. Subjects in these sessions faced added pressure to complete their tasks within a set time frame, influencing their cognitive and expressive behaviors, as depicted in Fig 3. A notable observation is the peak in the middle of the session in the expressive typing state for most subjects, indicating a concentrated effort to accomplish the majority of the writing task. This peak is a significant indicator of increased typing activity, possibly reflecting a heightened state of focused work. The cognitive arousal state, denoted as *x*_1_, is observed to be high at the beginning and end of these sessions. Initially, the subjects engage in intensive information gathering, which requires significant cognitive processing, thereby elevating the arousal state. As they transition from gathering to writing, a decrease in cognitive arousal is observed, coinciding with an increase in the expressive typing state. This inverse relationship between the two states highlights the shift from cognitive-intensive to expression-intensive phases of the task. Towards the end of the session, the cognitive arousal rises again, likely due to the subjects’ efforts to review and finalize their work within the time limit. This rise at the end underscores the return to cognitive-intensive activities, such as editing and revising the written content. The behavioral patterns observed in these time-limited sessions offer insightful revelations about the impact of time pressure on cognitive and expressive states, shedding light on how individuals manage their workload under constrained conditions.

#### Email interrupted sessions

The email-interrupted sessions in our study present an intriguing scenario where subjects encounter continual distractions in the form of emails, leading to varied patterns in cognitive and expressive states. This variability among subjects is particularly evident in their responses to interruptions, as some exhibit a marked change in their arousal state while others maintain a more steady level, reflecting their individual coping strategies with such disruptions. These interruptions inevitably impact the expressive typing state, typically causing a degradation, which can be attributed to the frequent breaks in concentration and the resultant shift in focus away from the primary task of essay writing. Unlike the time-limited sessions where distinct peaks in arousal and expressive typing states were observed, the patterns in the email-interrupted sessions do not exhibit such definitive forms. The lack of pronounced peaks in these sessions suggests a more erratic and disrupted workflow, indicating how the constant email notifications influence cognitive load and the ability to maintain a consistent level of expressive output. This observation sheds light on the impact of intermittent distractions on task performance, highlighting the need for strategies to manage such interruptions in work environments where maintaining focus is crucial for productivity.

#### State distribution for different office applications

**Fig 5:**
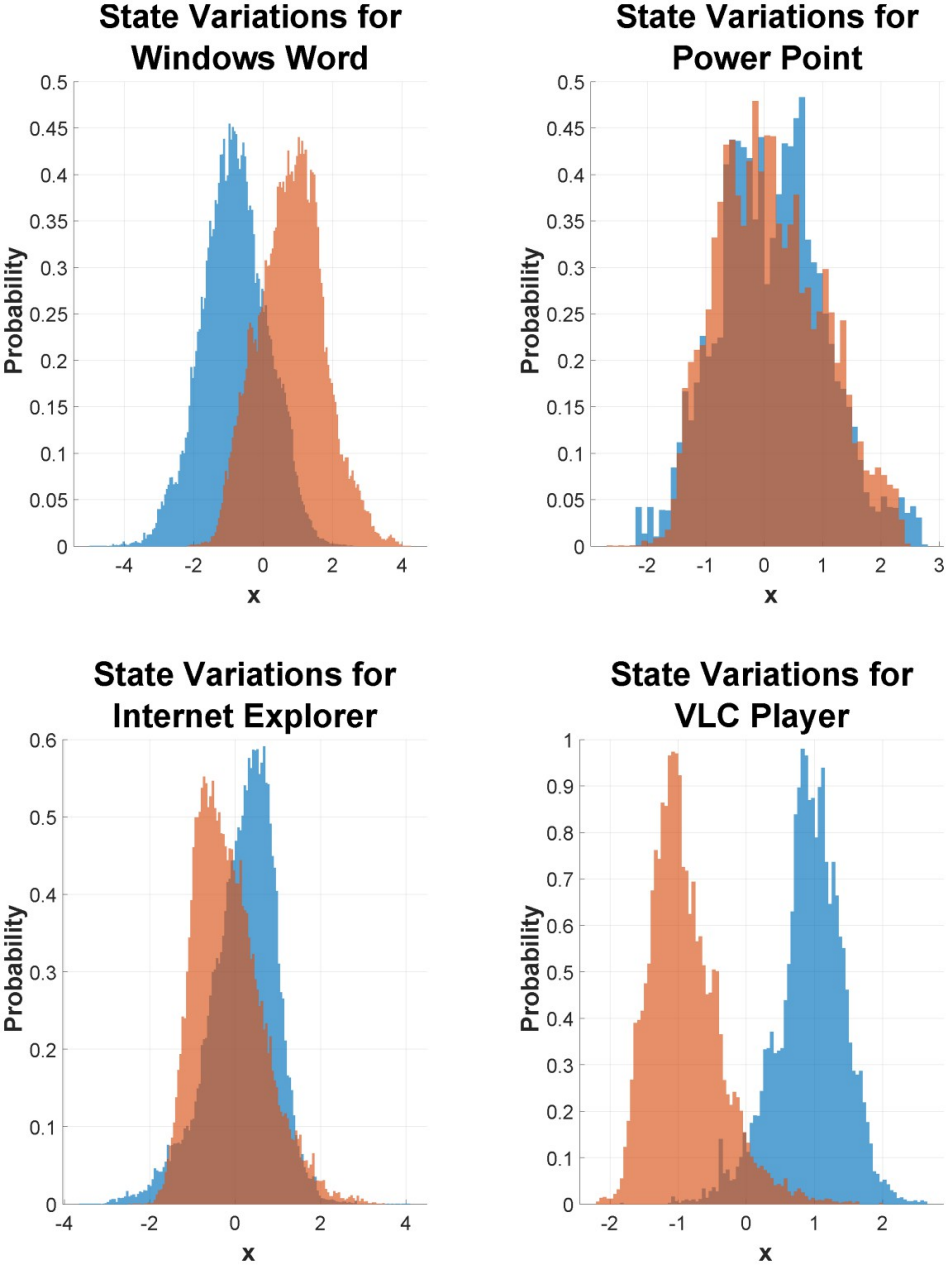
State distribution for different applications. The histograms above show the variations of state vectors *x*_1_ representing cognitive arousal state and *x*_2_ representing expressive typing state for four applications Word, PowerPoint, Internet Explorer, and VLC Player in order from left. The expressive typing state variations are shown in orange bars whereas the arousal state by blue bars. Notice the variations depend on the nature of the applications.

In our analysis, the visualization of cognitive arousal and expressive typing states with respect to the use of various applications offers insightful revelations about the subjects’ interaction with technology. By examining these states in the context of specific applications, we gain an understanding of how different tasks influence cognitive and expressive behaviors. For instance, as shown in Fig 5, subjects exhibit a higher level of cognitive arousal when engaged in responding to emails. This heightened arousal could be attributed to the immediate cognitive processing required to comprehend and respond to incoming information [1, 63]. Contrastingly, when subjects use Microsoft Word, a platform primarily for writing, they demonstrate a high expressive typing state, coupled with a relatively lower level of cognitive arousal. This pattern suggests a more focused and sustained engagement in the expressive aspect of the task. Furthermore, the use of applications like PowerPoint, which necessitates a combination of typing and cognitive efforts to create and structure documents, results in overlapping variations in the histograms of both cognitive arousal and expressive typing states. These overlapping variations highlight the dual demands of such applications, requiring both cognitive processing for conceptualizing content and expressive output for its articulation. The distinct patterns observed across different applications underscore the nuanced relationship between the nature of the task and the associated cognitive and expressive states, providing a window into how specific activities can differentially engage cognitive and expressive faculties.

The findings of this study have significant practical implications, especially in remote work environments where managing stress and maintaining productivity are crucial. Understanding the impact of different stressors and work activities on cognitive states can inform the design of more effective work strategies and tools. Additionally, the study lays the groundwork for future research, expanding the understanding of cognitive responses in work environments. Translating these findings into practice involves developing interventions and tools to manage stress and enhance productivity. By leveraging the study’s insights, practitioners can better understand and mitigate the impact of stressors in various work settings. Therefore, this line of research is crucial for advancing our understanding of workplace stress and productivity. It opens avenues for developing more adaptive and responsive work environments, ultimately contributing to improved well-being and efficiency. However, while the study provides valuable insights, it has some limitations, including the controlled nature of the experimental setup and the dataset’s sample size. Despite these limitations, the study’s multimodal approach and detailed analysis offer a comprehensive understanding of the interaction between stressors, cognitive states, and productivity.

## Implications of the study

The field of cognitive response analysis in work environments, a critical aspect of occupational psychology and human-computer interaction, is undergoing rapid evolution. This change is primarily driven by the technological advancements in data collection and analysis methodologies. In today’s era, marked by an increasing reliance on digital tools and remote working arrangements [64], understanding the nuances of cognitive responses to various stressors in the workplace is not just academically intriguing but also practically essential [65]. It has significant implications for enhancing both employee well-being and workplace productivity [66].

Our study builds upon the foundational work in state space modeling and Bayesian filters [30, 31, 33, 34, 37–47, 62], extending the knowledge frontier by integrating refined multi-state filtering methodologies. We delve deeper into the nuanced effects of stressors on cognitive and expressive states and their inter-relation, a subject that has been of perennial interest but inadequately explored due to technological and methodological limitations in the past. By employing a multi-state filtering technique, and leveraging a more diverse dataset, this research provides intricate insights into the dynamic interplay between various work-related stressors and cognitive responses. These insights are pivotal in our understanding of the cognitive load, a concept critical in designing effective and humane work environments.

The implications of our findings are far-reaching and multifaceted. Primarily, they contribute significantly to the discourse on workplace productivity and stress management. By systematically unraveling how different stressors—ranging from time constraints to intermittent distractions—affect cognitive arousal and expressive typing states, our study provides empirical evidence that can inform the design of workplace strategies and tools. These tools and strategies are not just theoretical constructs but have real-world applicability in enhancing work efficiency and reducing the detrimental effects of stress [67].

In the context of the evolving dynamics of the workplace, particularly with the surge in remote work and digital collaboration, the insights from our study are especially pertinent. Remote work, while offering flexibility and perceived freedom, brings with it unique challenges, including the blurring of work-life boundaries and the potential for increased stress due to isolation or overwork. Our findings offer a roadmap for navigating these challenges [68], helping organizations and individuals to strike a balance between productivity and well-being in these digitally mediated work environments.

Moreover, our research methodology and the resulting findings lay a solid foundation for future studies. The detailed analysis of cognitive responses to different stressors, conducted with rigor and precision, opens up new avenues for research. These include personalized stress management strategies and productivity enhancement techniques. Our study thus acts as a catalyst, spurring further exploration and innovation in this domain.

One of the most exciting prospects of this research is its potential contribution to HCI, especially to the development of intelligent tools for real-time stress and productivity monitoring by using underlying states. Such tools, harnessing the power of artificial intelligence and machine learning, could provide immediate feedback and interventions, helping individuals manage their workloads and stress levels more effectively as showcased in [60, 69]. This aligns with the broader trend of digitalization in the workplace [70] and represents a significant leap forward from traditional, more reactive approaches to workplace well-being.

The ultimate goal of this line of research is ambitious yet profoundly impactful: to achieve a comprehensive understanding of the cognitive impacts of various work-related stressors and to translate this understanding into practical tools and strategies. These tools and strategies aim to enhance productivity and well-being in the workplace, addressing one of the fundamental challenges of modern work life.

However, reaching this goal is not without its challenges. The primary challenge lies in the translation of theoretical knowledge and experimental findings into practical, real-world applications that are effective across diverse work environments and individual differences. This requires not just academic insight but also a deep understanding of the complexities and variabilities of real-world work settings in addition to strategic data collection that mimic practical use-cases.

To achieve this ambitious goal, interdisciplinary knowledge spanning psychology, data science, human-computer interaction, and occupational health is required. Moreover, technological advancements, particularly in non-invasive physiological monitoring [71], sophisticated data analytics [72], and AI-driven predictive modeling [73], are crucial. The development and refinement of such technologies will play a pivotal role in the practical application of our findings, bridging the gap between research and real-world application.

As workplaces continue to evolve, particularly with the increasing prevalence of remote work, the insights gained from this research will be invaluable. They will not only shape the future of work but also ensure that this future is more adaptable, efficient, and conducive to mental health and well-being. This research, therefore, stands at the intersection of technological innovation and human-centric design, heralding a new era in workplace productivity and health.

## Conclusion

In this study, we have explored the intricate relationship between expressive typing state, and cognitive arousal. Our research demonstrates the potential of multi-state filters in accurately tracking these states, offering a promising tool for performance tracking in both traditional and remote work environments. The utilization of such advanced algorithms could revolutionize how we understand and manage productivity and stress in the workplace. Theoretically, our findings contribute to the broader discourse on cognitive workload and its manifestation in behavioral patterns. Practically, the application of this research could lead to the development of intelligent systems capable of enhancing workplace efficiency by providing real-time feedback on cognitive states. While our results with simulated data show promise, we recognize the need for further refinement, particularly in modeling the complexity of real-world interactions. Future research directions include expanding the scope of variables considered, such as incorporating analysis of correct and incorrect key responses and utilizing marked point process observations based on physiological data. Additionally, integrating more complex interactions, like mouse usage and application switching, could provide a richer understanding of cognitive states. However, limitations in current datasets, such as the SWELL dataset’s lack of comprehensive typing data, highlight the need for more detailed experimental studies tailored to the evolving landscape of remote work.

## Supporting information

S1 Appendix Summary derivations. Derivations for different steps of the multi-state filter.
(PDF)

S1 FigLatent state estimations for experimental data with no stressors for subject 1.The panel shows the experimental data for no stressor sessions. From top, the binary variables *n*_1_ and *n*_2_ derived from deconvolved EDA data and typing data respectively, the continuous variable *r* denoting the RR intervals derived from heart rate (red line) and $\tilde{r}$ estimated from latent variables *x*_1_ and *x*_2_ (purple line), *x*_1_ and *x*_2_ in order from top indicating cognitive arousal state and expressive typing state respectively. *p*_1_ and *p*_2_ show the estimated probabilities. Patches of green, red, and cyan indicate what application the subject was using at the time of measurement. Green indicates applications for information search like internet explorer, red is for typing like Microsoft word and PowerPoint and cyan is for when subjects are looking at their emails. Finally, the QQ plot for the residual error of *r* is shown.(TIF)

S2 FigLatent state estimations for experimental data with time limit for subject 1.The panel shows the experimental data with time limit. From top, the binary variables *n*_1_ and *n*_2_ derived from deconvolved EDA data and typing data respectively, the continuous variable *r* denoting the RR intervals derived from heart rate (red line) and $\tilde{r}$ estimated from latent variables *x*_1_ and *x*_2_ (purple line), *x*_1_ and *x*_2_ in order from top indicating cognitive arousal state and expressive typing state respectively. *p*_1_ and *p*_2_ show the estimated probabilities. Patches of green, red, and cyan indicate what application the subject was using at the time of measurement. Green indicates applications for information search like internet explorer, red is for typing like Microsoft word and PowerPoint and cyan is for when subjects are looking at their emails. Finally, the QQ plot for the residual error of *r* is shown.(TIF)

S3 FigLatent state estimations for experimental data with interruptions for subject 1.The panel shows the experimental data with interruptions. From top, the binary variables *n*_1_ and *n*_2_ derived from deconvolved EDA data and typing data respectively, the continuous variable *r* denoting the RR intervals derived from heart rate (red line) and $\tilde{r}$ estimated from latent variables *x*_1_ and *x*_2_ (purple line), *x*_1_ and *x*_2_ in order from top indicating cognitive arousal state and expressive typing state respectively. *p*_1_ and *p*_2_ show the estimated probabilities. Patches of green, red, and cyan indicate what application the subject was using at the time of measurement. Green indicates applications for information search like internet explorer, red is for typing like Microsoft word and PowerPoint and cyan is for when subjects are looking at their emails. The Blue vertical line indicates the time email notifications were sent. Finally, the QQ plot for the residual error of *r* is shown.(TIF)

S4 FigLatent state estimations for experimental data with no stressors for subject 2.The panel shows the experimental data for no stressor sessions. From top, the binary variables *n*_1_ and *n*_2_ derived from deconvolved EDA data and typing data respectively, the continuous variable *r* denoting the RR intervals derived from heart rate (red line) and $\tilde{r}$ estimated from latent variables *x*_1_ and *x*_2_ (purple line), *x*_1_ and *x*_2_ in order from top indicating cognitive arousal state and expressive typing state respectively. *p*_1_ and *p*_2_ show the estimated probabilities. Patches of green, red, and cyan indicate what application the subject was using at the time of measurement. Green indicates applications for information search like internet explorer, red is for typing like Microsoft word and PowerPoint and cyan is for when subjects are looking at their emails. Finally, the QQ plot for the residual error of *r* is shown.(TIF)

S5 FigLatent state estimations for experimental data with time limit for subject 2.The panel shows the experimental data with time limit. From top, the binary variables *n*_1_ and *n*_2_ derived from deconvolved EDA data and typing data respectively, the continuous variable *r* denoting the RR intervals derived from heart rate (red line) and $\tilde{r}$ estimated from latent variables *x*_1_ and *x*_2_ (purple line), *x*_1_ and *x*_2_ in order from top indicating cognitive arousal state and expressive typing state respectively. *p*_1_ and *p*_2_ show the estimated probabilities. Patches of green, red, and cyan indicate what application the subject was using at the time of measurement. Green indicates applications for information search like internet explorer, red is for typing like Microsoft word and PowerPoint and cyan is for when subjects are looking at their emails. Finally, the QQ plot for the residual error of *r* is shown.(TIF)

S6 FigLatent state estimations for experimental data with interruptions for subject 2.The panel shows the experimental data with interruptions. From top, the binary variables *n*_1_ and *n*_2_ derived from deconvolved EDA data and typing data respectively, the continuous variable *r* denoting the RR intervals derived from heart rate (red line) and $\tilde{r}$ estimated from latent variables *x*_1_ and *x*_2_ (purple line), *x*_1_ and *x*_2_ in order from top indicating cognitive arousal state and expressive typing state respectively. *p*_1_ and *p*_2_ show the estimated probabilities. Patches of green, red, and cyan indicate what application the subject was using at the time of measurement. Green indicates applications for information search like internet explorer, red is for typing like Microsoft word and PowerPoint and cyan is for when subjects are looking at their emails. The Blue vertical line indicates the time email notifications were sent. Finally, the QQ plot for the residual error of *r* is shown.(TIF)

S7 FigLatent state estimations for experimental data with no stressors for subject 3.The panel shows the experimental data for no stressor sessions. From top, the binary variables *n*_1_ and *n*_2_ derived from deconvolved EDA data and typing data respectively, the continuous variable *r* denoting the RR intervals derived from heart rate (red line) and $\tilde{r}$ estimated from latent variables *x*_1_ and *x*_2_ (purple line), *x*_1_ and *x*_2_ in order from top indicating cognitive arousal state and expressive typing state respectively. *p*_1_ and *p*_2_ show the estimated probabilities. Patches of green, red, and cyan indicate what application the subject was using at the time of measurement. Green indicates applications for information search like internet explorer, red is for typing like Microsoft word and PowerPoint and cyan is for when subjects are looking at their emails. Finally, the QQ plot for the residual error of *r* is shown.(TIF)

S8 FigLatent state estimations for experimental data with time limit for subject 3.The panel shows the experimental data with time limit. From top, the binary variables *n*_1_ and *n*_2_ derived from deconvolved EDA data and typing data respectively, the continuous variable *r* denoting the RR intervals derived from heart rate (red line) and $\tilde{r}$ estimated from latent variables *x*_1_ and *x*_2_ (purple line), *x*_1_ and *x*_2_ in order from top indicating cognitive arousal state and expressive typing state respectively. *p*_1_ and *p*_2_ show the estimated probabilities. Patches of green, red, and cyan indicate what application the subject was using at the time of measurement. Green indicates applications for information search like internet explorer, red is for typing like Microsoft word and PowerPoint and cyan is for when subjects are looking at their emails. Finally, the QQ plot for the residual error of *r* is shown.(TIF)

S9 FigLatent state estimations for experimental data with interruptions for subject 3.The panel shows the experimental data with interruptions. From top, the binary variables *n*_1_ and *n*_2_ derived from deconvolved EDA data and typing data respectively, the continuous variable *r* denoting the RR intervals derived from heart rate (red line) and $\tilde{r}$ estimated from latent variables *x*_1_ and *x*_2_ (purple line), *x*_1_ and *x*_2_ in order from top indicating cognitive arousal state and expressive typing state respectively. *p*_1_ and *p*_2_ show the estimated probabilities. Patches of green, red, and cyan indicate what application the subject was using at the time of measurement. Green indicates applications for information search like internet explorer, red is for typing like Microsoft word and PowerPoint and cyan is for when subjects are looking at their emails. The Blue vertical line indicates the time email notifications were sent. Finally, the QQ plot for the residual error of *r* is shown.(TIF)

S10 FigLatent state estimations for experimental data with no stressors for subject 4.The panel shows the experimental data for no stressor sessions. From top, the binary variables *n*_1_ and *n*_2_ derived from deconvolved EDA data and typing data respectively, the continuous variable *r* denoting the RR intervals derived from heart rate (red line) and $\tilde{r}$ estimated from latent variables *x*_1_ and *x*_2_ (purple line), *x*_1_ and *x*_2_ in order from top indicating cognitive arousal state and expressive typing state respectively. *p*_1_ and *p*_2_ show the estimated probabilities. Patches of green, red, and cyan indicate what application the subject was using at the time of measurement. Green indicates applications for information search like internet explorer, red is for typing like Microsoft word and PowerPoint and cyan is for when subjects are looking at their emails. Finally, the QQ plot for the residual error of *r* is shown.(TIF)

S11 FigLatent state estimations for experimental data with time limit for subject 4.The panel shows the experimental data with time limit. From top, the binary variables *n*_1_ and *n*_2_ derived from deconvolved EDA data and typing data respectively, the continuous variable *r* denoting the RR intervals derived from heart rate (red line) and $\tilde{r}$ estimated from latent variables *x*_1_ and *x*_2_ (purple line), *x*_1_ and *x*_2_ in order from top indicating cognitive arousal state and expressive typing state respectively. *p*_1_ and *p*_2_ show the estimated probabilities. Patches of green, red, and cyan indicate what application the subject was using at the time of measurement. Green indicates applications for information search like internet explorer, red is for typing like Microsoft word and PowerPoint and cyan is for when subjects are looking at their emails. Finally, the QQ plot for the residual error of *r* is shown.(TIF)

S12 FigLatent state estimations for experimental data with interruptions for subject 4.The panel shows the experimental data with interruptions. From top, the binary variables *n*_1_ and *n*_2_ derived from deconvolved EDA data and typing data respectively, the continuous variable *r* denoting the RR intervals derived from heart rate (red line) and $\tilde{r}$ estimated from latent variables *x*_1_ and *x*_2_ (purple line), *x*_1_ and *x*_2_ in order from top indicating cognitive arousal state and expressive typing state respectively. *p*_1_ and *p*_2_ show the estimated probabilities. Patches of green, red, and cyan indicate what application the subject was using at the time of measurement. Green indicates applications for information search like internet explorer, red is for typing like Microsoft word and PowerPoint and cyan is for when subjects are looking at their emails. The Blue vertical line indicates the time email notifications were sent. Finally, the QQ plot for the residual error of *r* is shown.(TIF)

S13 FigLatent state estimations for experimental data with no stressors for subject 5.The panel shows the experimental data for no stressor sessions. From top, the binary variables *n*_1_ and *n*_2_ derived from deconvolved EDA data and typing data respectively, the continuous variable *r* denoting the RR intervals derived from heart rate (red line) and $\tilde{r}$ estimated from latent variables *x*_1_ and *x*_2_ (purple line), *x*_1_ and *x*_2_ in order from top indicating cognitive arousal state and expressive typing state respectively. *p*_1_ and *p*_2_ show the estimated probabilities. Patches of green, red, and cyan indicate what application the subject was using at the time of measurement. Green indicates applications for information search like internet explorer, red is for typing like Microsoft word and PowerPoint and cyan is for when subjects are looking at their emails. Finally, the QQ plot for the residual error of *r* is shown.(TIF)

S14 FigLatent state estimations for experimental data with time limit for subject 5.The panel shows the experimental data with time limit. From top, the binary variables *n*_1_ and *n*_2_ derived from deconvolved EDA data and typing data respectively, the continuous variable *r* denoting the RR intervals derived from heart rate (red line) and $\tilde{r}$ estimated from latent variables *x*_1_ and *x*_2_ (purple line), *x*_1_ and *x*_2_ in order from top indicating cognitive arousal state and expressive typing state respectively. *p*_1_ and *p*_2_ show the estimated probabilities. Patches of green, red, and cyan indicate what application the subject was using at the time of measurement. Green indicates applications for information search like internet explorer, red is for typing like Microsoft word and PowerPoint and cyan is for when subjects are looking at their emails. Finally, the QQ plot for the residual error of *r* is shown.(TIF)

S15 FigLatent state estimations for experimental data with interruptions for subject 5.The panel shows the experimental data with interruptions. From top, the binary variables *n*_1_ and *n*_2_ derived from deconvolved EDA data and typing data respectively, the continuous variable *r* denoting the RR intervals derived from heart rate (red line) and $\tilde{r}$ estimated from latent variables *x*_1_ and *x*_2_ (purple line), *x*_1_ and *x*_2_ in order from top indicating cognitive arousal state and expressive typing state respectively. *p*_1_ and *p*_2_ show the estimated probabilities. Patches of green, red, and cyan indicate what application the subject was using at the time of measurement. Green indicates applications for information search like internet explorer, red is for typing like Microsoft word and PowerPoint and cyan is for when subjects are looking at their emails. The Blue vertical line indicates the time email notifications were sent. Finally, the QQ plot for the residual error of *r* is shown.(TIF)

S16 FigLatent state estimations for experimental data with no stressors for subject 6.The panel shows the experimental data for no stressor sessions. From top, the binary variables *n*_1_ and *n*_2_ derived from deconvolved EDA data and typing data respectively, the continuous variable *r* denoting the RR intervals derived from heart rate (red line) and $\tilde{r}$ estimated from latent variables *x*_1_ and *x*_2_ (purple line), *x*_1_ and *x*_2_ in order from top indicating cognitive arousal state and expressive typing state respectively. *p*_1_ and *p*_2_ show the estimated probabilities. Patches of green, red, and cyan indicate what application the subject was using at the time of measurement. Green indicates applications for information search like internet explorer, red is for typing like Microsoft word and PowerPoint and cyan is for when subjects are looking at their emails. Finally, the QQ plot for the residual error of *r* is shown.(TIF)

S17 FigLatent state estimations for experimental data with time limit for subject 6.The panel shows the experimental data with time limit. From top, the binary variables *n*_1_ and *n*_2_ derived from deconvolved EDA data and typing data respectively, the continuous variable *r* denoting the RR intervals derived from heart rate (red line) and $\tilde{r}$ estimated from latent variables *x*_1_ and *x*_2_ (purple line), *x*_1_ and *x*_2_ in order from top indicating cognitive arousal state and expressive typing state respectively. *p*_1_ and *p*_2_ show the estimated probabilities. Patches of green, red, and cyan indicate what application the subject was using at the time of measurement. Green indicates applications for information search like internet explorer, red is for typing like Microsoft word and PowerPoint and cyan is for when subjects are looking at their emails. Finally, the QQ plot for the residual error of *r* is shown.(TIF)

S18 FigLatent state estimations for experimental data with interruptions for subject 6.The panel shows the experimental data with interruptions. From top, the binary variables *n*_1_ and *n*_2_ derived from deconvolved EDA data and typing data respectively, the continuous variable *r* denoting the RR intervals derived from heart rate (red line) and $\tilde{r}$ estimated from latent variables *x*_1_ and *x*_2_ (purple line), *x*_1_ and *x*_2_ in order from top indicating cognitive arousal state and expressive typing state respectively. *p*_1_ and *p*_2_ show the estimated probabilities. Patches of green, red, and cyan indicate what application the subject was using at the time of measurement. Green indicates applications for information search like internet explorer, red is for typing like Microsoft word and PowerPoint and cyan is for when subjects are looking at their emails. The Blue vertical line indicates the time email notifications were sent. Finally, the QQ plot for the residual error of *r* is shown.(TIF)

S19 FigLatent state estimations for experimental data with no stressors for subject 7.The panel shows the experimental data for no stressor sessions. From top, the binary variables *n*_1_ and *n*_2_ derived from deconvolved EDA data and typing data respectively, the continuous variable *r* denoting the RR intervals derived from heart rate (red line) and $\tilde{r}$ estimated from latent variables *x*_1_ and *x*_2_ (purple line), *x*_1_ and *x*_2_ in order from top indicating cognitive arousal state and expressive typing state respectively. *p*_1_ and *p*_2_ show the estimated probabilities. Patches of green, red, and cyan indicate what application the subject was using at the time of measurement. Green indicates applications for information search like internet explorer, red is for typing like Microsoft word and PowerPoint and cyan is for when subjects are looking at their emails. Finally, the QQ plot for the residual error of *r* is shown.(TIF)

S20 FigLatent state estimations for experimental data with time limit for subject 7.The panel shows the experimental data with time limit. From top, the binary variables *n*_1_ and *n*_2_ derived from deconvolved EDA data and typing data respectively, the continuous variable *r* denoting the RR intervals derived from heart rate (red line) and $\tilde{r}$ estimated from latent variables *x*_1_ and *x*_2_ (purple line), *x*_1_ and *x*_2_ in order from top indicating cognitive arousal state and expressive typing state respectively. *p*_1_ and *p*_2_ show the estimated probabilities. Patches of green, red, and cyan indicate what application the subject was using at the time of measurement. Green indicates applications for information search like internet explorer, red is for typing like Microsoft word and PowerPoint and cyan is for when subjects are looking at their emails. Finally, the QQ plot for the residual error of *r* is shown.(TIF)

S21 FigLatent state estimations for experimental data with interruptions for subject 7.The panel shows the experimental data with interruptions. From top, the binary variables *n*_1_ and *n*_2_ derived from deconvolved EDA data and typing data respectively, the continuous variable *r* denoting the RR intervals derived from heart rate (red line) and $\tilde{r}$ estimated from latent variables *x*_1_ and *x*_2_ (purple line), *x*_1_ and *x*_2_ in order from top indicating cognitive arousal state and expressive typing state respectively. *p*_1_ and *p*_2_ show the estimated probabilities. Patches of green, red, and cyan indicate what application the subject was using at the time of measurement. Green indicates applications for information search like internet explorer, red is for typing like Microsoft word and PowerPoint and cyan is for when subjects are looking at their emails. The Blue vertical line indicates the time email notifications were sent. Finally, the QQ plot for the residual error of *r* is shown.(TIF)

S22 FigLatent state estimations for experimental data with no stressors for subject 8.The panel shows the experimental data for no stressor sessions. From top, the binary variables *n*_1_ and *n*_2_ derived from deconvolved EDA data and typing data respectively, the continuous variable *r* denoting the RR intervals derived from heart rate (red line) and $\tilde{r}$ estimated from latent variables *x*_1_ and *x*_2_ (purple line), *x*_1_ and *x*_2_ in order from top indicating cognitive arousal state and expressive typing state respectively. *p*_1_ and *p*_2_ show the estimated probabilities. Patches of green, red, and cyan indicate what application the subject was using at the time of measurement. Green indicates applications for information search like internet explorer, red is for typing like Microsoft word and PowerPoint and cyan is for when subjects are looking at their emails. Finally, the QQ plot for the residual error of *r* is shown.(TIF)

S23 FigLatent state estimations for experimental data with time limit for subject 8.The panel shows the experimental data with time limit. From top, the binary variables *n*_1_ and *n*_2_ derived from deconvolved EDA data and typing data respectively, the continuous variable *r* denoting the RR intervals derived from heart rate (red line) and $\tilde{r}$ estimated from latent variables *x*_1_ and *x*_2_ (purple line), *x*_1_ and *x*_2_ in order from top indicating cognitive arousal state and expressive typing state respectively. *p*_1_ and *p*_2_ show the estimated probabilities. Patches of green, red, and cyan indicate what application the subject was using at the time of measurement. Green indicates applications for information search like internet explorer, red is for typing like Microsoft word and PowerPoint and cyan is for when subjects are looking at their emails. Finally, the QQ plot for the residual error of *r* is shown.(TIF)

S24 FigLatent state estimations for experimental data with interruptions for subject 8.The panel shows the experimental data with interruptions. From top, the binary variables *n*_1_ and *n*_2_ derived from deconvolved EDA data and typing data respectively, the continuous variable *r* denoting the RR intervals derived from heart rate (red line) and $\tilde{r}$ estimated from latent variables *x*_1_ and *x*_2_ (purple line), *x*_1_ and *x*_2_ in order from top indicating cognitive arousal state and expressive typing state respectively. *p*_1_ and *p*_2_ show the estimated probabilities. Patches of green, red, and cyan indicate what application the subject was using at the time of measurement. Green indicates applications for information search like internet explorer, red is for typing like Microsoft word and PowerPoint and cyan is for when subjects are looking at their emails. The Blue vertical line indicates the time email notifications were sent. Finally, the QQ plot for the residual error of *r* is shown.(TIF)

S25 FigLatent state estimations for experimental data with no stressors for subject 9.The panel shows the experimental data for no stressor sessions. From top, the binary variables *n*_1_ and *n*_2_ derived from deconvolved EDA data and typing data respectively, the continuous variable *r* denoting the RR intervals derived from heart rate (red line) and $\tilde{r}$ estimated from latent variables *x*_1_ and *x*_2_ (purple line), *x*_1_ and *x*_2_ in order from top indicating cognitive arousal state and expressive typing state respectively. *p*_1_ and *p*_2_ show the estimated probabilities. Patches of green, red, and cyan indicate what application the subject was using at the time of measurement. Green indicates applications for information search like internet explorer, red is for typing like Microsoft word and PowerPoint and cyan is for when subjects are looking at their emails. Finally, the QQ plot for the residual error of *r* is shown.(TIF)

S26 FigLatent state estimations for experimental data with time limit for subject 9.The panel shows the experimental data with time limit. From top, the binary variables *n*_1_ and *n*_2_ derived from deconvolved EDA data and typing data respectively, the continuous variable *r* denoting the RR intervals derived from heart rate (red line) and $\tilde{r}$ estimated from latent variables *x*_1_ and *x*_2_ (purple line), *x*_1_ and *x*_2_ in order from top indicating cognitive arousal state and expressive typing state respectively. *p*_1_ and *p*_2_ show the estimated probabilities. Patches of green, red, and cyan indicate what application the subject was using at the time of measurement. Green indicates applications for information search like internet explorer, red is for typing like Microsoft word and PowerPoint and cyan is for when subjects are looking at their emails. Finally, the QQ plot for the residual error of *r* is shown.(TIF)

S27 FigLatent state estimations for experimental data with interruptions for subject 9.The panel shows the experimental data with interruptions. From top, the binary variables *n*_1_ and *n*_2_ derived from deconvolved EDA data and typing data respectively, the continuous variable *r* denoting the RR intervals derived from heart rate (red line) and $\tilde{r}$ estimated from latent variables *x*_1_ and *x*_2_ (purple line), *x*_1_ and *x*_2_ in order from top indicating cognitive arousal state and expressive typing state respectively. *p*_1_ and *p*_2_ show the estimated probabilities. Patches of green, red, and cyan indicate what application the subject was using at the time of measurement. Green indicates applications for information search like internet explorer, red is for typing like Microsoft word and PowerPoint and cyan is for when subjects are looking at their emails. The Blue vertical line indicates the time email notifications were sent. Finally, the QQ plot for the residual error of *r* is shown.(TIF)

S28 FigLatent state estimations for experimental data with no stressors for subject 10.The panel shows the experimental data for no stressor sessions. From top, the binary variables *n*_1_ and *n*_2_ derived from deconvolved EDA data and typing data respectively, the continuous variable *r* denoting the RR intervals derived from heart rate (red line) and $\tilde{r}$ estimated from latent variables *x*_1_ and *x*_2_ (purple line), *x*_1_ and *x*_2_ in order from top indicating cognitive arousal state and expressive typing state respectively. *p*_1_ and *p*_2_ show the estimated probabilities. Patches of green, red, and cyan indicate what application the subject was using at the time of measurement. Green indicates applications for information search like internet explorer, red is for typing like Microsoft word and PowerPoint and cyan is for when subjects are looking at their emails. Finally, the QQ plot for the residual error of *r* is shown.(TIF)

S29 FigLatent state estimations for experimental data with time limit for subject 10.The panel shows the experimental data with time limit. From top, the binary variables *n*_1_ and *n*_2_ derived from deconvolved EDA data and typing data respectively, the continuous variable *r* denoting the RR intervals derived from heart rate (red line) and $\tilde{r}$ estimated from latent variables *x*_1_ and *x*_2_ (purple line), *x*_1_ and *x*_2_ in order from top indicating cognitive arousal state and expressive typing state respectively. *p*_1_ and *p*_2_ show the estimated probabilities. Patches of green, red, and cyan indicate what application the subject was using at the time of measurement. Green indicates applications for information search like internet explorer, red is for typing like Microsoft word and PowerPoint and cyan is for when subjects are looking at their emails. Finally, the QQ plot for the residual error of *r* is shown.(TIF)

S30 FigLatent state estimations for experimental data with interruptions for subject 10.The panel shows the experimental data with interruptions. From top, the binary variables *n*_1_ and *n*_2_ derived from deconvolved EDA data and typing data respectively, the continuous variable *r* denoting the RR intervals derived from heart rate (red line) and $\tilde{r}$ estimated from latent variables *x*_1_ and *x*_2_ (purple line), *x*_1_ and *x*_2_ in order from top indicating cognitive arousal state and expressive typing state respectively. *p*_1_ and *p*_2_ show the estimated probabilities. Patches of green, red, and cyan indicate what application the subject was using at the time of measurement. Green indicates applications for information search like internet explorer, red is for typing like Microsoft word and PowerPoint and cyan is for when subjects are looking at their emails. The Blue vertical line indicates the time email notifications were sent. Finally, the QQ plot for the residual error of *r* is shown.(TIF)

S31 FigLatent state estimations for experimental data with no stressors for subject 12.The panel shows the experimental data for no stressor sessions. From top, the binary variables *n*_1_ and *n*_2_ derived from deconvolved EDA data and typing data respectively, the continuous variable *r* denoting the RR intervals derived from heart rate (red line) and $\tilde{r}$ estimated from latent variables *x*_1_ and *x*_2_ (purple line), *x*_1_ and *x*_2_ in order from top indicating cognitive arousal state and expressive typing state respectively. *p*_1_ and *p*_2_ show the estimated probabilities. Patches of green, red, and cyan indicate what application the subject was using at the time of measurement. Green indicates applications for information search like internet explorer, red is for typing like Microsoft word and PowerPoint and cyan is for when subjects are looking at their emails. Finally, the QQ plot for the residual error of *r* is shown.(TIF)

S32 FigLatent state estimations for experimental data with time limit for subject 12.The panel shows the experimental data with time limit. From top, the binary variables *n*_1_ and *n*_2_ derived from deconvolved EDA data and typing data respectively, the continuous variable *r* denoting the RR intervals derived from heart rate (red line) and $\tilde{r}$ estimated from latent variables *x*_1_ and *x*_2_ (purple line), *x*_1_ and *x*_2_ in order from top indicating cognitive arousal state and expressive typing state respectively. *p*_1_ and *p*_2_ show the estimated probabilities. Patches of green, red, and cyan indicate what application the subject was using at the time of measurement. Green indicates applications for information search like internet explorer, red is for typing like Microsoft word and PowerPoint and cyan is for when subjects are looking at their emails. Finally, the QQ plot for the residual error of *r* is shown.(TIF)

S33 FigLatent state estimations for experimental data with interruptions for subject 12.The panel shows the experimental data with interruptions. From top, the binary variables *n*_1_ and *n*_2_ derived from deconvolved EDA data and typing data respectively, the continuous variable *r* denoting the RR intervals derived from heart rate (red line) and $\tilde{r}$ estimated from latent variables *x*_1_ and *x*_2_ (purple line), *x*_1_ and *x*_2_ in order from top indicating cognitive arousal state and expressive typing state respectively. *p*_1_ and *p*_2_ show the estimated probabilities. Patches of green, red, and cyan indicate what application the subject was using at the time of measurement. Green indicates applications for information search like internet explorer, red is for typing like Microsoft word and PowerPoint and cyan is for when subjects are looking at their emails. The Blue vertical line indicates the time email notifications were sent. Finally, the QQ plot for the residual error of *r* is shown.(TIF)

S34 FigLatent state estimations for experimental data with no stressors for subject 13.The panel shows the experimental data for no stressor sessions. From top, the binary variables *n*_1_ and *n*_2_ derived from deconvolved EDA data and typing data respectively, the continuous variable *r* denoting the RR intervals derived from heart rate (red line) and $\tilde{r}$ estimated from latent variables *x*_1_ and *x*_2_ (purple line), *x*_1_ and *x*_2_ in order from top indicating cognitive arousal state and expressive typing state respectively. *p*_1_ and *p*_2_ show the estimated probabilities. Patches of green, red, and cyan indicate what application the subject was using at the time of measurement. Green indicates applications for information search like internet explorer, red is for typing like Microsoft word and PowerPoint and cyan is for when subjects are looking at their emails. Finally, the QQ plot for the residual error of *r* is shown.(TIF)

S35 FigLatent state estimations for experimental data with time limit for subject 13.The panel shows the experimental data with time limit. From top, the binary variables *n*_1_ and *n*_2_ derived from deconvolved EDA data and typing data respectively, the continuous variable *r* denoting the RR intervals derived from heart rate (red line) and $\tilde{r}$ estimated from latent variables *x*_1_ and *x*_2_ (purple line), *x*_1_ and *x*_2_ in order from top indicating cognitive arousal state and expressive typing state respectively. *p*_1_ and *p*_2_ show the estimated probabilities. Patches of green, red, and cyan indicate what application the subject was using at the time of measurement. Green indicates applications for information search like internet explorer, red is for typing like Microsoft word and PowerPoint and cyan is for when subjects are looking at their emails. Finally, the QQ plot for the residual error of *r* is shown.(TIF)

S36 FigLatent state estimations for experimental data with interruptions for subject 13.The panel shows the experimental data with interruptions. From top, the binary variables *n*_1_ and *n*_2_ derived from deconvolved EDA data and typing data respectively, the continuous variable *r* denoting the RR intervals derived from heart rate (red line) and $\tilde{r}$ estimated from latent variables *x*_1_ and *x*_2_ (purple line), *x*_1_ and *x*_2_ in order from top indicating cognitive arousal state and expressive typing state respectively. *p*_1_ and *p*_2_ show the estimated probabilities. Patches of green, red, and cyan indicate what application the subject was using at the time of measurement. Green indicates applications for information search like internet explorer, red is for typing like Microsoft word and PowerPoint and cyan is for when subjects are looking at their emails. The Blue vertical line indicates the time email notifications were sent. Finally, the QQ plot for the residual error of *r* is shown.(TIF)

S37 FigLatent state estimations for experimental data with no stressors for subject 15.The panel shows the experimental data for no stressor sessions. From top, the binary variables *n*_1_ and *n*_2_ derived from deconvolved EDA data and typing data respectively, the continuous variable *r* denoting the RR intervals derived from heart rate (red line) and $\tilde{r}$ estimated from latent variables *x*_1_ and *x*_2_ (purple line), *x*_1_ and *x*_2_ in order from top indicating cognitive arousal state and expressive typing state respectively. *p*_1_ and *p*_2_ show the estimated probabilities. Patches of green, red, and cyan indicate what application the subject was using at the time of measurement. Green indicates applications for information search like internet explorer, red is for typing like Microsoft word and PowerPoint and cyan is for when subjects are looking at their emails. Finally, the QQ plot for the residual error of *r* is shown.(TIF)

S38 FigLatent state estimations for experimental data with time limit for subject 15.The panel shows the experimental data with time limit. From top, the binary variables *n*_1_ and *n*_2_ derived from deconvolved EDA data and typing data respectively, the continuous variable *r* denoting the RR intervals derived from heart rate (red line) and $\tilde{r}$ estimated from latent variables *x*_1_ and *x*_2_ (purple line), *x*_1_ and *x*_2_ in order from top indicating cognitive arousal state and expressive typing state respectively. *p*_1_ and *p*_2_ show the estimated probabilities. Patches of green, red, and cyan indicate what application the subject was using at the time of measurement. Green indicates applications for information search like internet explorer, red is for typing like Microsoft word and PowerPoint and cyan is for when subjects are looking at their emails. Finally, the QQ plot for the residual error of *r* is shown.(TIF)

S39 FigLatent state estimations for experimental data with interruptions for subject 15.The panel shows the experimental data with interruptions. From top, the binary variables *n*_1_ and *n*_2_ derived from deconvolved EDA data and typing data respectively, the continuous variable *r* denoting the RR intervals derived from heart rate (red line) and $\tilde{r}$ estimated from latent variables *x*_1_ and *x*_2_ (purple line), *x*_1_ and *x*_2_ in order from top indicating cognitive arousal state and expressive typing state respectively. *p*_1_ and *p*_2_ show the estimated probabilities. Patches of green, red, and cyan indicate what application the subject was using at the time of measurement. Green indicates applications for information search like internet explorer, red is for typing like Microsoft word and PowerPoint and cyan is for when subjects are looking at their emails. The Blue vertical line indicates the time email notifications were sent. Finally, the QQ plot for the residual error of *r* is shown.(TIF)

S40 FigLatent state estimations for experimental data with no stressors for subject 16.The panel shows the experimental data for no stressor sessions. From top, the binary variables *n*_1_ and *n*_2_ derived from deconvolved EDA data and typing data respectively, the continuous variable *r* denoting the RR intervals derived from heart rate (red line) and $\tilde{r}$ estimated from latent variables *x*_1_ and *x*_2_ (purple line), *x*_1_ and *x*_2_ in order from top indicating cognitive arousal state and expressive typing state respectively. *p*_1_ and *p*_2_ show the estimated probabilities. Patches of green, red, and cyan indicate what application the subject was using at the time of measurement. Green indicates applications for information search like internet explorer, red is for typing like Microsoft word and PowerPoint and cyan is for when subjects are looking at their emails. Finally, the QQ plot for the residual error of *r* is shown.(TIF)

S41 FigLatent state estimations for experimental data with time limit for subject 16.The panel shows the experimental data with time limit. From top, the binary variables *n*_1_ and *n*_2_ derived from deconvolved EDA data and typing data respectively, the continuous variable *r* denoting the RR intervals derived from heart rate (red line) and $\tilde{r}$ estimated from latent variables *x*_1_ and *x*_2_ (purple line), *x*_1_ and *x*_2_ in order from top indicating cognitive arousal state and expressive typing state respectively. *p*_1_ and *p*_2_ show the estimated probabilities. Patches of green, red, and cyan indicate what application the subject was using at the time of measurement. Green indicates applications for information search like internet explorer, red is for typing like Microsoft word and PowerPoint and cyan is for when subjects are looking at their emails. Finally, the QQ plot for the residual error of *r* is shown.(TIF)

S42 FigLatent state estimations for experimental data with interruptions for subject 16.The panel shows the experimental data with interruptions. From top, the binary variables *n*_1_ and *n*_2_ derived from deconvolved EDA data and typing data respectively, the continuous variable *r* denoting the RR intervals derived from heart rate (red line) and $\tilde{r}$ estimated from latent variables *x*_1_ and *x*_2_ (purple line), *x*_1_ and *x*_2_ in order from top indicating cognitive arousal state and expressive typing state respectively. *p*_1_ and *p*_2_ show the estimated probabilities. Patches of green, red, and cyan indicate what application the subject was using at the time of measurement. Green indicates applications for information search like internet explorer, red is for typing like Microsoft word and PowerPoint and cyan is for when subjects are looking at their emails. The Blue vertical line indicates the time email notifications were sent. Finally, the QQ plot for the residual error of *r* is shown.(TIF)

S43 FigLatent state estimations for experimental data with no stressors for subject 17.The panel shows the experimental data for no stressor sessions. From top, the binary variables *n*_1_ and *n*_2_ derived from deconvolved EDA data and typing data respectively, the continuous variable *r* denoting the RR intervals derived from heart rate (red line) and $\tilde{r}$ estimated from latent variables *x*_1_ and *x*_2_ (purple line), *x*_1_ and *x*_2_ in order from top indicating cognitive arousal state and expressive typing state respectively. *p*_1_ and *p*_2_ show the estimated probabilities. Patches of green, red, and cyan indicate what application the subject was using at the time of measurement. Green indicates applications for information search like internet explorer, red is for typing like Microsoft word and PowerPoint and cyan is for when subjects are looking at their emails. Finally, the QQ plot for the residual error of *r* is shown.(TIF)

S44 FigLatent state estimations for experimental data with time limit for subject 17.The panel shows the experimental data with time limit. From top, the binary variables *n*_1_ and *n*_2_ derived from deconvolved EDA data and typing data respectively, the continuous variable *r* denoting the RR intervals derived from heart rate (red line) and $\tilde{r}$ estimated from latent variables *x*_1_ and *x*_2_ (purple line), *x*_1_ and *x*_2_ in order from top indicating cognitive arousal state and expressive typing state respectively. *p*_1_ and *p*_2_ show the estimated probabilities. Patches of green, red, and cyan indicate what application the subject was using at the time of measurement. Green indicates applications for information search like internet explorer, red is for typing like Microsoft word and PowerPoint and cyan is for when subjects are looking at their emails. Finally, the QQ plot for the residual error of *r* is shown.(TIF)

S45 FigLatent state estimations for experimental data with interruptions for subject 17.The panel shows the experimental data with interruptions. From top, the binary variables *n*_1_ and *n*_2_ derived from deconvolved EDA data and typing data respectively, the continuous variable *r* denoting the RR intervals derived from heart rate (red line) and $\tilde{r}$ estimated from latent variables *x*_1_ and *x*_2_ (purple line), *x*_1_ and *x*_2_ in order from top indicating cognitive arousal state and expressive typing state respectively. *p*_1_ and *p*_2_ show the estimated probabilities. Patches of green, red, and cyan indicate what application the subject was using at the time of measurement. Green indicates applications for information search like internet explorer, red is for typing like Microsoft word and PowerPoint and cyan is for when subjects are looking at their emails. The Blue vertical line indicates the time email notifications were sent. Finally, the QQ plot for the residual error of *r* is shown.(TIF)

S46 FigLatent state estimations for experimental data with no stressors for subject 18.The panel shows the experimental data for no stressor sessions. From top, the binary variables *n*_1_ and *n*_2_ derived from deconvolved EDA data and typing data respectively, the continuous variable *r* denoting the RR intervals derived from heart rate (red line) and $\tilde{r}$ estimated from latent variables *x*_1_ and *x*_2_ (purple line), *x*_1_ and *x*_2_ in order from top indicating cognitive arousal state and expressive typing state respectively. *p*_1_ and *p*_2_ show the estimated probabilities. Patches of green, red, and cyan indicate what application the subject was using at the time of measurement. Green indicates applications for information search like internet explorer, red is for typing like Microsoft word and PowerPoint and cyan is for when subjects are looking at their emails. Finally, the QQ plot for the residual error of *r* is shown.(TIF)

S47 FigLatent state estimations for experimental data with time limit for subject 18.The panel shows the experimental data with time limit. From top, the binary variables *n*_1_ and *n*_2_ derived from deconvolved EDA data and typing data respectively, the continuous variable *r* denoting the RR intervals derived from heart rate (red line) and $\tilde{r}$ estimated from latent variables *x*_1_ and *x*_2_ (purple line), *x*_1_ and *x*_2_ in order from top indicating cognitive arousal state and expressive typing state respectively. *p*_1_ and *p*_2_ show the estimated probabilities. Patches of green, red, and cyan indicate what application the subject was using at the time of measurement. Green indicates applications for information search like internet explorer, red is for typing like Microsoft word and PowerPoint and cyan is for when subjects are looking at their emails. Finally, the QQ plot for the residual error of *r* is shown.(TIF)

S48 FigLatent state estimations for experimental data with interruptions for subject 18.The panel shows the experimental data with interruptions. From top, the binary variables *n*_1_ and *n*_2_ derived from deconvolved EDA data and typing data respectively, the continuous variable *r* denoting the RR intervals derived from heart rate (red line) and $\tilde{r}$ estimated from latent variables *x*_1_ and *x*_2_ (purple line), *x*_1_ and *x*_2_ in order from top indicating cognitive arousal state and expressive typing state respectively. *p*_1_ and *p*_2_ show the estimated probabilities. Patches of green, red, and cyan indicate what application the subject was using at the time of measurement. Green indicates applications for information search like internet explorer, red is for typing like Microsoft word and PowerPoint and cyan is for when subjects are looking at their emails. The Blue vertical line indicates the time email notifications were sent. Finally, the QQ plot for the residual error of *r* is shown.(TIF)

S49 FigLatent state estimations for experimental data with no stressors for subject 19.The panel shows the experimental data for no stressor sessions. From top, the binary variables *n*_1_ and *n*_2_ derived from deconvolved EDA data and typing data respectively, the continuous variable *r* denoting the RR intervals derived from heart rate (red line) and $\tilde{r}$ estimated from latent variables *x*_1_ and *x*_2_ (purple line), *x*_1_ and *x*_2_ in order from top indicating cognitive arousal state and expressive typing state respectively. *p*_1_ and *p*_2_ show the estimated probabilities. Patches of green, red, and cyan indicate what application the subject was using at the time of measurement. Green indicates applications for information search like internet explorer, red is for typing like Microsoft word and PowerPoint and cyan is for when subjects are looking at their emails. Finally, the QQ plot for the residual error of *r* is shown.(TIF)

S50 FigLatent state estimations for experimental data with time limit for subject 19.The panel shows the experimental data with time limit. From top, the binary variables *n*_1_ and *n*_2_ derived from deconvolved EDA data and typing data respectively, the continuous variable *r* denoting the RR intervals derived from heart rate (red line) and $\tilde{r}$ estimated from latent variables *x*_1_ and *x*_2_ (purple line), *x*_1_ and *x*_2_ in order from top indicating cognitive arousal state and expressive typing state respectively. *p*_1_ and *p*_2_ show the estimated probabilities. Patches of green, red, and cyan indicate what application the subject was using at the time of measurement. Green indicates applications for information search like internet explorer, red is for typing like Microsoft word and PowerPoint and cyan is for when subjects are looking at their emails. Finally, the QQ plot for the residual error of *r* is shown.(TIF)

S51 FigLatent state estimations for experimental data with interruptions for subject 19.The panel shows the experimental data with interruptions. From top, the binary variables *n*_1_ and *n*_2_ derived from deconvolved EDA data and typing data respectively, the continuous variable *r* denoting the RR intervals derived from heart rate (red line) and $\tilde{r}$ estimated from latent variables *x*_1_ and *x*_2_ (purple line), *x*_1_ and *x*_2_ in order from top indicating cognitive arousal state and expressive typing state respectively. *p*_1_ and *p*_2_ show the estimated probabilities. Patches of green, red, and cyan indicate what application the subject was using at the time of measurement. Green indicates applications for information search like internet explorer, red is for typing like Microsoft word and PowerPoint and cyan is for when subjects are looking at their emails. The Blue vertical line indicates the time email notifications were sent. Finally, the QQ plot for the residual error of *r* is shown.(TIF)

S52 FigLatent state estimations for experimental data with no stressors for subject 20.The panel shows the experimental data for no stressor sessions. From top, the binary variables *n*_1_ and *n*_2_ derived from deconvolved EDA data and typing data respectively, the continuous variable *r* denoting the RR intervals derived from heart rate (red line) and $\tilde{r}$ estimated from latent variables *x*_1_ and *x*_2_ (purple line), *x*_1_ and *x*_2_ in order from top indicating cognitive arousal state and expressive typing state respectively. *p*_1_ and *p*_2_ show the estimated probabilities. Patches of green, red, and cyan indicate what application the subject was using at the time of measurement. Green indicates applications for information search like internet explorer, red is for typing like Microsoft word and PowerPoint and cyan is for when subjects are looking at their emails. Finally, the QQ plot for the residual error of *r* is shown.(TIF)

S53 FigLatent state estimations for experimental data with time limit for subject 20.The panel shows the experimental data with time limit. From top, the binary variables *n*_1_ and *n*_2_ derived from deconvolved EDA data and typing data respectively, the continuous variable *r* denoting the RR intervals derived from heart rate (red line) and $\tilde{r}$ estimated from latent variables *x*_1_ and *x*_2_ (purple line), *x*_1_ and *x*_2_ in order from top indicating cognitive arousal state and expressive typing state respectively. *p*_1_ and *p*_2_ show the estimated probabilities. Patches of green, red, and cyan indicate what application the subject was using at the time of measurement. Green indicates applications for information search like internet explorer, red is for typing like Microsoft word and PowerPoint and cyan is for when subjects are looking at their emails. Finally, the QQ plot for the residual error of *r* is shown.(TIF)

S54 FigLatent state estimations for experimental data with interruptions for subject 20.The panel shows the experimental data with interruptions. From top, the binary variables *n*_1_ and *n*_2_ derived from deconvolved EDA data and typing data respectively, the continuous variable *r* denoting the RR intervals derived from heart rate (red line) and $\tilde{r}$ estimated from latent variables *x*_1_ and *x*_2_ (purple line), *x*_1_ and *x*_2_ in order from top indicating cognitive arousal state and expressive typing state respectively. *p*_1_ and *p*_2_ show the estimated probabilities. Patches of green, red, and cyan indicate what application the subject was using at the time of measurement. Green indicates applications for information search like internet explorer, red is for typing like Microsoft word and PowerPoint and cyan is for when subjects are looking at their emails. The Blue vertical line indicates the time email notifications were sent. Finally, the QQ plot for the residual error of *r* is shown.(TIF)

S55 FigLatent state estimations for experimental data with no stressors for subject 21.The panel shows the experimental data for no stressor sessions. From top, the binary variables *n*_1_ and *n*_2_ derived from deconvolved EDA data and typing data respectively, the continuous variable *r* denoting the RR intervals derived from heart rate (red line) and $\tilde{r}$ estimated from latent variables *x*_1_ and *x*_2_ (purple line), *x*_1_ and *x*_2_ in order from top indicating cognitive arousal state and expressive typing state respectively. *p*_1_ and *p*_2_ show the estimated probabilities. Patches of green, red, and cyan indicate what application the subject was using at the time of measurement. Green indicates applications for information search like internet explorer, red is for typing like Microsoft word and PowerPoint and cyan is for when subjects are looking at their emails. Finally, the QQ plot for the residual error of *r* is shown.(TIF)

S56 FigLatent state estimations for experimental data with time limit for subject 21.The panel shows the experimental data with time limit. From top, the binary variables *n*_1_ and *n*_2_ derived from deconvolved EDA data and typing data respectively, the continuous variable *r* denoting the RR intervals derived from heart rate (red line) and $\tilde{r}$ estimated from latent variables *x*_1_ and *x*_2_ (purple line), *x*_1_ and *x*_2_ in order from top indicating cognitive arousal state and expressive typing state respectively. *p*_1_ and *p*_2_ show the estimated probabilities. Patches of green, red, and cyan indicate what application the subject was using at the time of measurement. Green indicates applications for information search like internet explorer, red is for typing like Microsoft word and PowerPoint and cyan is for when subjects are looking at their emails. Finally, the QQ plot for the residual error of *r* is shown.(TIF)

S57 FigLatent state estimations for experimental data with interruptions for subject 21.The panel shows the experimental data with interruptions. From top, the binary variables *n*_1_ and *n*_2_ derived from deconvolved EDA data and typing data respectively, the continuous variable *r* denoting the RR intervals derived from heart rate (red line) and $\tilde{r}$ estimated from latent variables *x*_1_ and *x*_2_ (purple line), *x*_1_ and *x*_2_ in order from top indicating cognitive arousal state and expressive typing state respectively. *p*_1_ and *p*_2_ show the estimated probabilities. Patches of green, red, and cyan indicate what application the subject was using at the time of measurement. Green indicates applications for information search like internet explorer, red is for typing like Microsoft word and PowerPoint and cyan is for when subjects are looking at their emails. The Blue vertical line indicates the time email notifications were sent. Finally, the QQ plot for the residual error of *r* is shown.(TIF)

S58 FigLatent state estimations for experimental data with no stressors for subject 22.The panel shows the experimental data for no stressor sessions. From top, the binary variables *n*_1_ and *n*_2_ derived from deconvolved EDA data and typing data respectively, the continuous variable *r* denoting the RR intervals derived from heart rate (red line) and $\tilde{r}$ estimated from latent variables *x*_1_ and *x*_2_ (purple line), *x*_1_ and *x*_2_ in order from top indicating cognitive arousal state and expressive typing state respectively. *p*_1_ and *p*_2_ show the estimated probabilities. Patches of green, red, and cyan indicate what application the subject was using at the time of measurement. Green indicates applications for information search like internet explorer, red is for typing like Microsoft word and PowerPoint and cyan is for when subjects are looking at their emails. Finally, the QQ plot for the residual error of *r* is shown.(TIF)

S59 FigLatent state estimations for experimental data with time limit for subject 22.The panel shows the experimental data with time limit. From top, the binary variables *n*_1_ and *n*_2_ derived from deconvolved EDA data and typing data respectively, the continuous variable *r* denoting the RR intervals derived from heart rate (red line) and $\tilde{r}$ estimated from latent variables *x*_1_ and *x*_2_ (purple line), *x*_1_ and *x*_2_ in order from top indicating cognitive arousal state and expressive typing state respectively. *p*_1_ and *p*_2_ show the estimated probabilities. Patches of green, red, and cyan indicate what application the subject was using at the time of measurement. Green indicates applications for information search like internet explorer, red is for typing like Microsoft word and PowerPoint and cyan is for when subjects are looking at their emails. Finally, the QQ plot for the residual error of *r* is shown.(TIF)

S60 FigLatent state estimations for experimental data with interruptions for subject 22.The panel shows the experimental data with interruptions. From top, the binary variables *n*_1_ and *n*_2_ derived from deconvolved EDA data and typing data respectively, the continuous variable *r* denoting the RR intervals derived from heart rate (red line) and $\tilde{r}$ estimated from latent variables *x*_1_ and *x*_2_ (purple line), *x*_1_ and *x*_2_ in order from top indicating cognitive arousal state and expressive typing state respectively. *p*_1_ and *p*_2_ show the estimated probabilities. Patches of green, red, and cyan indicate what application the subject was using at the time of measurement. Green indicates applications for information search like internet explorer, red is for typing like Microsoft word and PowerPoint and cyan is for when subjects are looking at their emails. The Blue vertical line indicates the time email notifications were sent. Finally, the QQ plot for the residual error of *r* is shown.(TIF)
