# Supplementary material for: Unveiling productivity: The interplay of cognitive arousal and expressive typing in remote work
Source: PLoS One. 2024 May 15;19(5):e0300786. doi: 10.1371/journal.pone.0300786 (PMC11095729; doi:10.1371/journal.pone.0300786)
Supplement: S1 Appendix — Derivations for different steps of the multi-state filter. (PDF) [file pone.0300786.s001.pdf]

Summary Derivation of E-Step Updates: To derive the filter step equations, we approximate the posterior density as a Gaussian distribution. Therefore, we have

$$
\begin{aligned}
e^{-\frac{1}{2}(\boldsymbol{x}_k-\boldsymbol{x}_{k|k})^T \boldsymbol{W}_{k|k}^{-1}(\boldsymbol{x}_k-\boldsymbol{x}_{k|k})}& \\
&\propto e^{n_{k,1}\log p_{k,1}+(1-n_{k,1})\log(1-p_{k,1})} \\
&\times e^{n_{k,2}\log p_{k,2}+(1-n_{k,2})\log(1-p_{k,2})} \\
&\times e^{-\frac{\left(r_k-\gamma_0-\gamma_1 x_{k,1}-\gamma_2 x_{k,2}\right)^2}{2\sigma_v^2}} \\
&\times e^{-\frac{1}{2}(\boldsymbol{x}_k-\boldsymbol{x}_{k|k-1})^T \boldsymbol{W}_{k|k-1}^{-1}(\boldsymbol{x}_k-\boldsymbol{x}_{k|k-1})}. \qquad (1)
\end{aligned}
$$

Now taking the logarithm of Eq (1) and ignoring the constant of proportionality, we obtain

$$
\begin{aligned}
-\frac{1}{2}&\left(\boldsymbol{x}_k-\boldsymbol{x}_{k|k}\right)^T \boldsymbol{W}_{k|k}^{-1}\left(\boldsymbol{x}_k-\boldsymbol{x}_{k|k}\right) \\
&= n_{k,1}\log p_{k,1}+(1-n_{k,1})\log(1-p_{k,1}) \\
&+ n_{k,2}\log p_{k,2}+(1-n_{k,2})\log(1-p_{k,2}) \\
&- \frac{\left(r_k-\gamma_0-\gamma_1 x_{1,k}-\gamma_2 x_{2,k}\right)^2}{2\sigma_v^2} \\
&- \frac{1}{2}\left(\boldsymbol{x}_k-\boldsymbol{x}_{k|k-1}\right)^T \boldsymbol{W}_{k|k-1}^{-1}\left(\boldsymbol{x}_k-\boldsymbol{x}_{k|k-1}\right)+\log C, \qquad (2)
\end{aligned}
$$

where C is the constant of proportionality. Eq (2) simplifies to

$$
\begin{aligned}
-\frac{1}{2}&(\boldsymbol{x}_k-\boldsymbol{x}_{k|k})^T \boldsymbol{W}_{k|k}^{-1}(\boldsymbol{x}_k-\boldsymbol{x}_{k|k}) \\
&= f(\boldsymbol{x}_k)-\frac{1}{2}(\boldsymbol{x}_k-\boldsymbol{x}_{k|k-1})^T \boldsymbol{W}_{k|k-1}^{-1}(\boldsymbol{x}_k-\boldsymbol{x}_{k|k-1}), \qquad (3)
\end{aligned}
$$

where

$$
\begin{aligned}
f(\boldsymbol{x}_k) &= n_{k,1}\log p_{k,1}+(1-n_{k,1})\log(1-p_{k,1}) \\
&+ n_{k,2}\log(p_{k,2})+(1-n_{k,2})\log(1-p_{k,2}) \\
&- \frac{(r_k-\gamma_0-\gamma_1 x_{1,k}-\gamma_2 x_{2,k})^2}{2\sigma_v^2}+\log C. \qquad (4)
\end{aligned}
$$

We follow previous methods to estimate our $\boldsymbol{x}_{k|k}$ and $\boldsymbol{W}_{k|k}$. Here, differentiating Eq (3) we get,

$$
\boldsymbol{W}_{k|k}^{-1}(\boldsymbol{x}_k-\boldsymbol{x}_{k|k}) = \boldsymbol{W}_{k|k-1}^{-1}(\boldsymbol{x}_k-\boldsymbol{x}_{k|k-1}) - \frac{\partial f(\boldsymbol{x}_k)}{\partial \boldsymbol{x}_k}. \qquad (5)
$$

From Eq (5), we can obtain $\boldsymbol{x}_{k|k}$ by setting $x_k = x_{k|k}$ which makes the RHS equal to 0. Then solving we get,

$$
\boldsymbol{x}_{k|k} = \boldsymbol{x}_{k|k-1} + \boldsymbol{W}_{k|k-1}\frac{\partial f(\boldsymbol{x}_k)}{\partial \boldsymbol{x}_k}\Big|_{x_{k|k}}, \qquad (6)
$$

where

$$
\frac{\partial f(\boldsymbol{x}_k)}{\partial \boldsymbol{x}_k} = \begin{bmatrix} n_{k,1}-p_{k,1}-\frac{\gamma_1(r_k-\gamma_0-\gamma_1 x_{1,k}-\gamma_2 x_{2,k}}{\sigma_v^2}) \\ n_{k,2}-p_{k,2}-\frac{\gamma_2(r_k-\gamma_0-\gamma_1 x_{1,k}-\gamma_2 x_{2,k}}{\sigma_v^2}). \end{bmatrix}. \qquad (7)
$$

Equation (6) can be solved using the Newton-Rapshon method. Differentiating (6) again we have the update for $\boldsymbol{W}_{k|k}$ as

$$\boldsymbol{W}_{k|k} = \left(\boldsymbol{W}_{k|k-1}^{-1} - \left. \frac{\partial^2 f(\boldsymbol{x}_k)}{\partial \boldsymbol{x}_k^2} \right|_{\boldsymbol{x}_{k|k}} \right)^{-1}, \tag{8}$$

where

$$\frac{\partial^2 f(\boldsymbol{x}_k)}{\partial \boldsymbol{x}_k^2} = \begin{bmatrix} -p_{k,1}(1-p_{k,1}) - \frac{\gamma_1^2}{\sigma_v^2} & \frac{\gamma_1\gamma_2}{\sigma_v^2} \\ \frac{\gamma_1\gamma_2}{\sigma_v^2} & -p_{k,2}(1-p_{k,2}) - \frac{\gamma_2^2}{\sigma_v^2} \end{bmatrix}. \tag{9}$$

After proceeding in the forward direction, we obtain a smoother estimate in the backward direction, thereby making use of all the observations to determine improved state space estimates at each point. The backward smoother equations are:

$$\boldsymbol{A}_k = \boldsymbol{W}_{k|k}\boldsymbol{W}_{k+1|k}^{-1} \tag{10}$$

$$\boldsymbol{x}_{k|K} = \boldsymbol{x}_{k|k} + \boldsymbol{A}_k \left(\boldsymbol{x}_{k+1|K} - \boldsymbol{x}_{k+1|k} \right) \tag{11}$$

$$\boldsymbol{W}_{k|K} = \boldsymbol{W}_{k|k} + \boldsymbol{A}_k^2 \left(\boldsymbol{W}_{k+1|K} - \boldsymbol{W}_{k+1|k} \right). \tag{12}$$

Summary Derivation of M-step derivations Letting $\boldsymbol{X}^K = \{x_1, x_2, ...x_K\}$ and considering that the latent variables are independent random variables, the complete data likelihood conditioned on the model parameters Θ is given by,

$$\begin{aligned} P(\boldsymbol{Y}^K, \boldsymbol{X}^K|\Theta) = & \prod_{k=1}^{K} p_{k,1}^{n_{k,1}} (1-p_{k,1})^{1-n_{k,1}} \\ & \times \prod_{k=1}^{K} p_{k,2}^{m_k} (1-p_{k=k,2})^{1-n_{k,2}} \\ & \times \prod_{k=1}^{J} \frac{1}{\sqrt{2\pi\sigma_v^2}} e^{-\frac{(r_k-\gamma_0-\gamma_1 x_{k,1}-\gamma_2 x_{k,2})^2}{2\sigma_v^2}} \\ & \times \prod_{k=1}^{J} \frac{1}{\sqrt{2\pi\sigma_{e_1}\sigma_{e_2}}} e^{-\frac{1}{2}\left(\left(\frac{x_{k,1}-x_{k-1,1}}{\sigma_{e_1}}\right)^2 + \left(\frac{x_{k,2}-x_{k-1,2}}{\sigma_{e_2}}\right)^2\right)}. \end{aligned} \tag{13}$$

The expected log-likelihood is

$$
\begin{aligned}
Q = &\sum_{k=1}^{J} \mathbb{E}\left[n_{k,1}(\beta_1 + x_{k,1}) - \log\left(1 + e^{\beta_1 + x_{k,1}}\right)\right] \\
&+ \sum_{k=1}^{J} \mathbb{E}\left[m_k(\beta_2 + x_{k,2}) - \log\left(1 + e^{\beta_2 + x_{k,2}}\right)\right] \\
&- \frac{K}{2}\log\left(2\pi\sigma_v^2\right) \\
&- \sum_{k=1}^{J} \frac{1}{2\sigma_v^2}\,\mathbb{E}\left[\left(r_k - \gamma_0 - \gamma_1 x_{k,1} - \gamma_2 x_{k,2}\right)^2\right] \\
&- \frac{K}{2}\log\left(2\pi\sigma_{\epsilon,1}\sigma_{\epsilon,2}\right) - \\
&\sum_{k=1}^{J} \frac{\mathbb{E}\left[\left(x_{k,1} - x_{k-1,1}\right)^2\right]}{2\sigma_{\epsilon,1}^2} + \frac{\mathbb{E}\left[\left(x_{k,2} - x_{k-1,2}\right)^2\right]}{2\sigma_{\epsilon,2}^2}. \qquad (14)
\end{aligned}
$$

M-step Updates for γ_0, γ_1, and γ_2

Assuming $x_{k|K,1}$ and $x_{k|K,2}$ are independent with zero co-variance and the variance of $x_{k|K,1}$ and $x_{k|K,2}$ obtained from $\boldsymbol{W}_{k|K}$ is given by $\sigma^2_{k|K,1}$ and $\sigma^2_{k|K,2}$ respectively, we define:

$$
\mathbb{E}[x_{k|K,1}x_{k|K,2}] = x_{k|K,1}x_{k|K,2} \qquad (15)
$$

$$
\mathbb{E}[x^2_{k|K,1}] = u_{k|K,1} = x^2_{k|K,1} + \sigma^2_{k|K,1} \qquad (16)
$$

$$
\mathbb{E}[x^2_{k|K,2}] = u_{k|K,2} = x^2_{k|K,2} + \sigma^2_{k|K,2}. \qquad (17)
$$

Therefore, taking the partial derivatives with respect to γ_0, γ_1 and γ_2 and setting them to 0 yield

$$
\begin{aligned}
\sum_{k=1}^{K} r_k - K\gamma_0 - \gamma_1\sum_{k=1}^{K} x_{k|K,1} - \gamma_2\sum_{k=1}^{K} x_{k|K,2} &= 0 \\
\sum_{k=1}^{K} r_k x_{k|K,1} - \gamma_0 \sum_{k=1}^{K} x_{k|K,1} - \gamma_1\sum_{k=1}^{K} u_{k|K,1} & \\
-\gamma_2\sum_{k=1}^{K} x_{k|K,1}x_{k|K,2} &= 0 \\
\sum_{k=1}^{K} r_k x_{k|K,2} - \gamma_0 \sum_{k=1}^{K} x_{k|K,2} - \gamma_1\sum_{k=1}^{K} x_{k|K,1}x_{k|K,2} & \\
-\gamma_2\sum_{k=1}^{K} u_{k|K,2} &= 0. \qquad (18)
\end{aligned}
$$

M-step updates for σ_v Taking the partial derivative with respect to

σ_v^2 and setting it to 0 yields

$$\begin{aligned}\sigma_v^2 = \frac{1}{K}\Bigg\{&\sum_{k=1}^{K} r_k^2 + K\gamma_0^2 + \gamma_1^2\sum_{k=1}^{K} u_{k|K,1} + \gamma_2^2\sum_{k=1}^{K} u_{k|K,2}\\ &- 2\gamma_0\sum_{k=1}^{K} r_k - 2\sum_{k=1}^{K} r_k x_{k|K,1} - 2\sum_{k=1}^{K} r_k x_{k|K,2}\\ &+2\gamma_0\sum_{k=1}^{K} x_{k|K,1} + 2\gamma_0\sum_{k=1}^{K} x_{k|K,2} + \sum_{k=1}^{K} x_{k|K,1}x_{k|K,2}\Bigg\}. \end{aligned} \tag{19}$$

M-step updates for $\sigma_{\epsilon,1}$ and $\sigma_{\epsilon,2}$
Let a_k be the diagonal elements of A_k. We define:

$$\begin{aligned}\mathbb{E}[x_{k|K,1}x_{k+1|K,1}] &= v_{k|K,1}\\ &= x_{k|K,1}x_{k+1|K,1} + a_{k,1}\sigma^2_{k|K,1} \end{aligned}\tag{20}$$

$$\begin{aligned}\mathbb{E}[x_{k|K,2}x_{k+1|K,2}] &= v_{k|K,2}\\ &= x_{k|K,2}x_{k+1|K,2} + a_{k,2}\sigma^2_{k|K,2}. \end{aligned}\tag{21}$$

Taking the partial derivative with respect to $\sigma^2_{\epsilon,1}$ and $\sigma^2_{\epsilon,2}$ and setting it to 0 yields

$$\begin{aligned}\sigma^2_{\epsilon,1} &= \frac{2}{K}\left\{\sum_{k=1}^{K} u_{k|K,1} - 2\sum_{k=1}^{K} v_{k|K,1} + \sum_{k=1}^{K} u_{k-1|K,1}\right\}\\ \sigma^2_{\epsilon,2} &= \frac{2}{K}\left\{\sum_{k=1}^{K} u_{k|K,2} - 2\sum_{k=1}^{K} v_{k|K,2} + \sum_{k=1}^{K} u_{k-1|K,2}\right\}. \end{aligned}\tag{22}$$

M-step Expected Log-Likelihood. Letting $\boldsymbol{X}^J = \{x_1, x_2, ...x_J\}$, the complete data likelihood conditioned on the model parameters Θ is given by,

$$\begin{aligned}P(\boldsymbol{Y}^J, \boldsymbol{X}^J|\Theta) = &\prod_{j=1}^{J} p_j^{n_j}(1-p_j)^{1-n_j} \times \prod_{j=1}^{J} q_j^{m_j}(1-q_j)^{1-q_j} \times\\ &\prod_{j=1}^{J}\frac{1}{\sqrt{2\pi\sigma_v^2}} e^{\frac{(r_j-\gamma_0-\gamma_1 x_{j,1}-\gamma_2 x_{j,2})^2}{2\sigma_v^2}} \times\\ &\prod_{j=1}^{J}\frac{1}{\sqrt{2\pi\sigma_{e_1}\sigma_{e_2}}} e^{\frac{1}{2}\left(\left(\frac{x_{j,1}-x_{j-1,1}}{\sigma_{e_1}}\right)^2+\left(\frac{x_{j,2}-x_{j-1,2}}{\sigma_{e_2}}\right)^2\right)} \end{aligned}\tag{23}$$

The expected log-likelihood is

$$
\begin{aligned}
Q = &\sum_{j=1}^{J} \mathbb{E}\left[n_j(\beta_1 + x_{j,1}) - \log\left(1 + e^{\beta_1 + x_{j,1}}\right)\right] \\
&+ \sum_{j=1}^{J} \mathbb{E}\left[m_j(\beta_2 + x_{j,2}) - \log\left(1 + e^{\beta_2 + x_{j,2}}\right)\right] \\
&- \frac{J}{2} log\left(2\pi\sigma_v^2\right) - \sum_{j=1}^{J} \frac{1}{2\sigma_v^2} \mathbb{E}\left[\left(r_j - \gamma_0 - \gamma_1 x_{j,1} - \gamma_2 x_{j,2}\right)^2\right] \\
&- \frac{J}{2} log\left(2\pi\sigma_{\epsilon,1}\sigma_{\epsilon,2}\right) - \sum_{j=1}^{J} \frac{\mathbb{E}\left[x_{j,1} - x_{j-1,1}\right]^2}{2\sigma_{\epsilon,1}^2} + \frac{\mathbb{E}\left[x_{j,2} - x_{j-1,2}\right]^2}{2\sigma_{\epsilon,2}^2}
\end{aligned} \tag{24}
$$

M-step Updates for γ_0, γ_1, and γ_2. Taking the partial derivatives with respect to γ_0, γ_1 and γ_2 and setting them to 0 yield

$$
\frac{\partial Q}{\partial \gamma_0} = \sum_{j=1}^{J} 2\,\mathbb{E}\left[r_j - \gamma_0 - \gamma_1 x_{j,1} - \gamma_2 x_{j,2}\right]
$$

$$
\implies \sum_{j=1}^{J} r_j - J\gamma_0 - \gamma_1 \sum_{j=1}^{J} x_{j|J,1} - \gamma_2 \sum_{j=1}^{J} x_{j|J,2} = 0 \tag{25}
$$

$$
\frac{\partial Q}{\partial \gamma_1} = \sum_{j=1}^{J} 2\,\mathbb{E}\left[x_{j,1}\left(r_j - \gamma_0 - \gamma_1 x_{j,1} - \gamma_2 x_{j,2}\right)\right]
$$

$$
\implies \sum_{j=1}^{J} r_j x_{j|J,1} - \gamma_0 \sum_{j=1}^{J} x_{j|J,1} - \gamma_1 \sum_{j=1}^{J} x_{j|J,1}^2 - \gamma_2 \sum_{j=1}^{J} x_{j|J,1} x_{j|J,2} = 0 \tag{26}
$$

$$
\frac{\partial Q}{\partial \gamma_2} = \sum_{j=1}^{J} 2\,\mathbb{E}\left[x_{j,2}\left(r_j - \gamma_0 - \gamma_1 x_{j,1} - \gamma_2 x_{j,2}\right)\right]
$$

$$
\implies \sum_{j=1}^{J} r_j x_{j|J,2} - \gamma_0 \sum_{j=1}^{J} x_{j|J,2} - \gamma_1 \sum_{j=1}^{J} x_{j|J,1} x_{j|J,2} - \gamma_2 \sum_{j=1}^{J} x_{j|J,2}^2 = 0 \tag{27}
$$

M-step updates for σ_v. Taking the partial derivative with respect to σ_v^2 and setting it to 0 yields

$$
\frac{\partial Q}{\partial \sigma_v^2} = -\frac{J}{2\sigma_v^2} + \frac{1}{2\sigma_v^4} \sum_{j=1}^{J} \mathbb{E}\left[\left(r_j - \gamma_0 - \gamma_1 x_{j,1} - \gamma_2 x_{j,2}\right)^2\right] = 0
$$

$$
\begin{aligned}
\implies \sigma_v^2 = \frac{1}{J} \Bigg\{ &\sum_{j=1}^{J} r_j^2 + J\gamma_0^2 + \gamma_1^2 \sum_{j=1}^{J} x_{j|J,1}^2 + \gamma_2^2 \sum_{j=1}^{J} x_{j|J,2}^2 \\
&- 2\gamma_0 \sum_{j=1}^{J} r_j - 2 \sum_{j=1}^{J} r_j x_{j|J,1} - 2 \sum_{j=1}^{J} r_j x_{j|J,2} \\
&+ 2\gamma_0 \sum_{j=1}^{J} x_{j|J,1} + 2\gamma_0 \sum_{j=1}^{J} x_{j|J,2} + \sum_{j=1}^{J} x_{j|J,1} x_{j|J,2} \Bigg\}
\end{aligned} \tag{28}
$$

M-step updates for $\sigma_{\epsilon,1}$ and $\sigma_{\epsilon,2}$. Taking the partial derivative with respect to $\sigma^2_{\epsilon,1}$ and $\sigma^2_{\epsilon,2}$ and setting it to 0 yields

$$\frac{\partial Q}{\partial \sigma^2_{\epsilon,1}} = -\frac{J}{4\sigma^2_{\epsilon,1}} + \frac{1}{2\sigma^4_{\epsilon,1}} \sum_{j=1}^{J} \mathbb{E}\left[x_{j,1} - x_{j-1,1}\right]^2 = 0$$

$$\implies \sigma^2_{\epsilon,1} = \frac{2}{J}\left\{\sum_{j=1}^{J} x^2_{j|J,1} - 2\sum_{j=1}^{J} x_{j|J,1}x_{j-1|J,1} \sum_{j=1}^{J} x^2_{j-1|J,1}\right\} \tag{29}$$

$$\frac{\partial Q}{\partial \sigma^2_{\epsilon,2}} = -\frac{J}{4\sigma^2_{\epsilon,2}} + \frac{1}{2\sigma^4_{\epsilon,2}} \sum_{j=1}^{J} \mathbb{E}\left[x_{j,2} - x_{j-1,2}\right]^2 = 0$$

$$\implies \sigma^2_{\epsilon,2} = \frac{2}{J}\left\{\sum_{j=1}^{J} x^2_{j|J,2} - 2\sum_{j=1}^{J} x_{j|J,2}x_{j-1|J,2} \sum_{j=1}^{J} x^2_{j-1|J,2}\right\} \tag{30}$$
