# Supplementary figures and images for: Unveiling productivity: The interplay of cognitive arousal and expressive typing in remote work
Source: PLoS One. 2024 May 15;19(5):e0300786. doi: 10.1371/journal.pone.0300786 (PMC11095729; doi:10.1371/journal.pone.0300786)

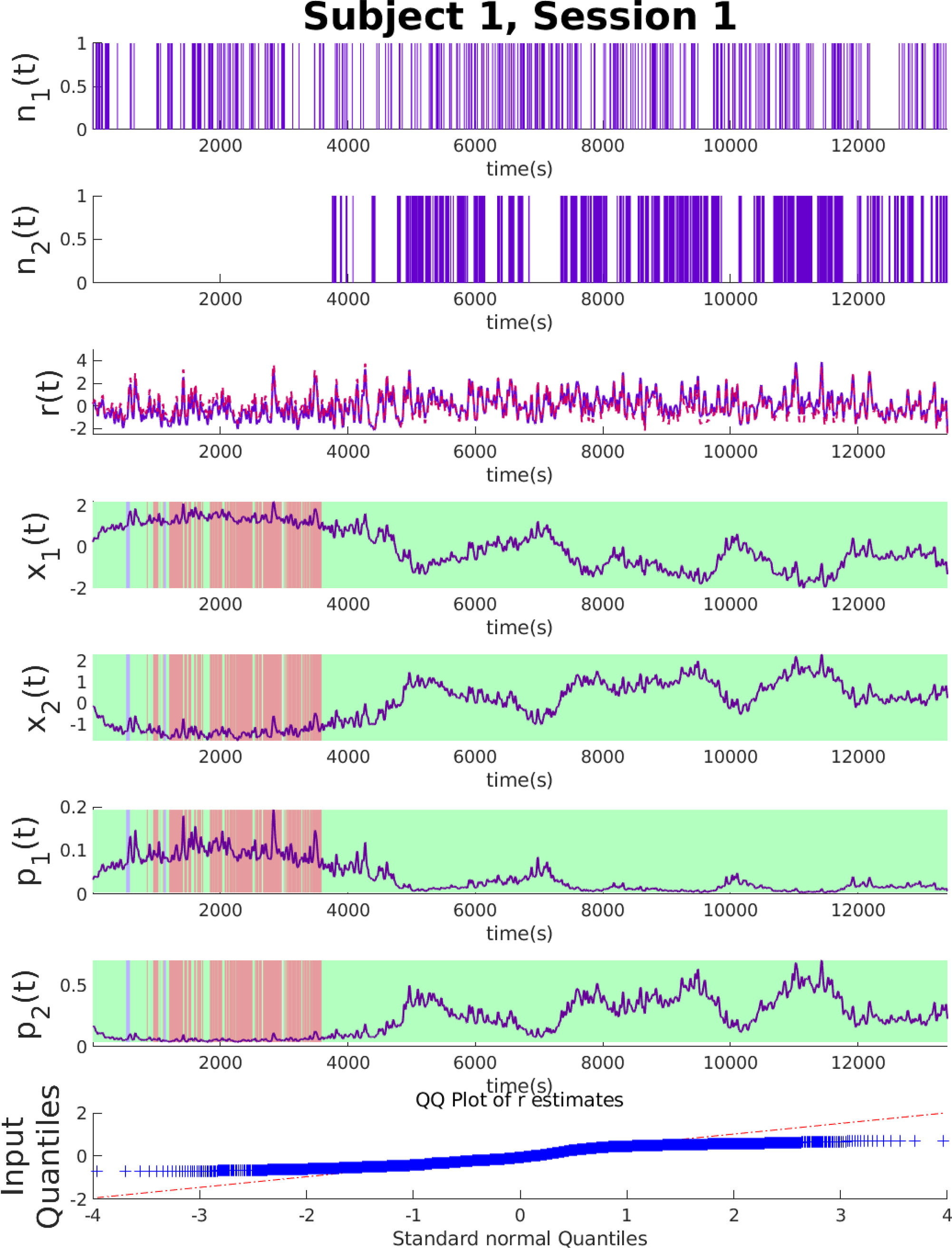

Supplement: S1 Fig — The panel shows the experimental data for no stressor sessions. From top, the binary variables n1 and n2 derived from deconvolved EDA data and typing data respectively, the continuous variable r denoting the RR intervals derived from heart rate (red line) and r˜ estimated from latent variables x1 and x2 (purple line), x1 and x2 in order from top indicating cognitive arousal state and expressive typing state respectively. p1 and p2 show the estimated probabilities. Patches of green, red, and cyan indicate what application the subject was using at the time of measurement. Green indicates applications for information search like internet explorer, red is for typing like Microsoft word and PowerPoint and cyan is for when subjects are looking at their emails. Finally, the QQ plot for the residual error of r is shown. (TIF) [file pone.0300786.s002.tif]

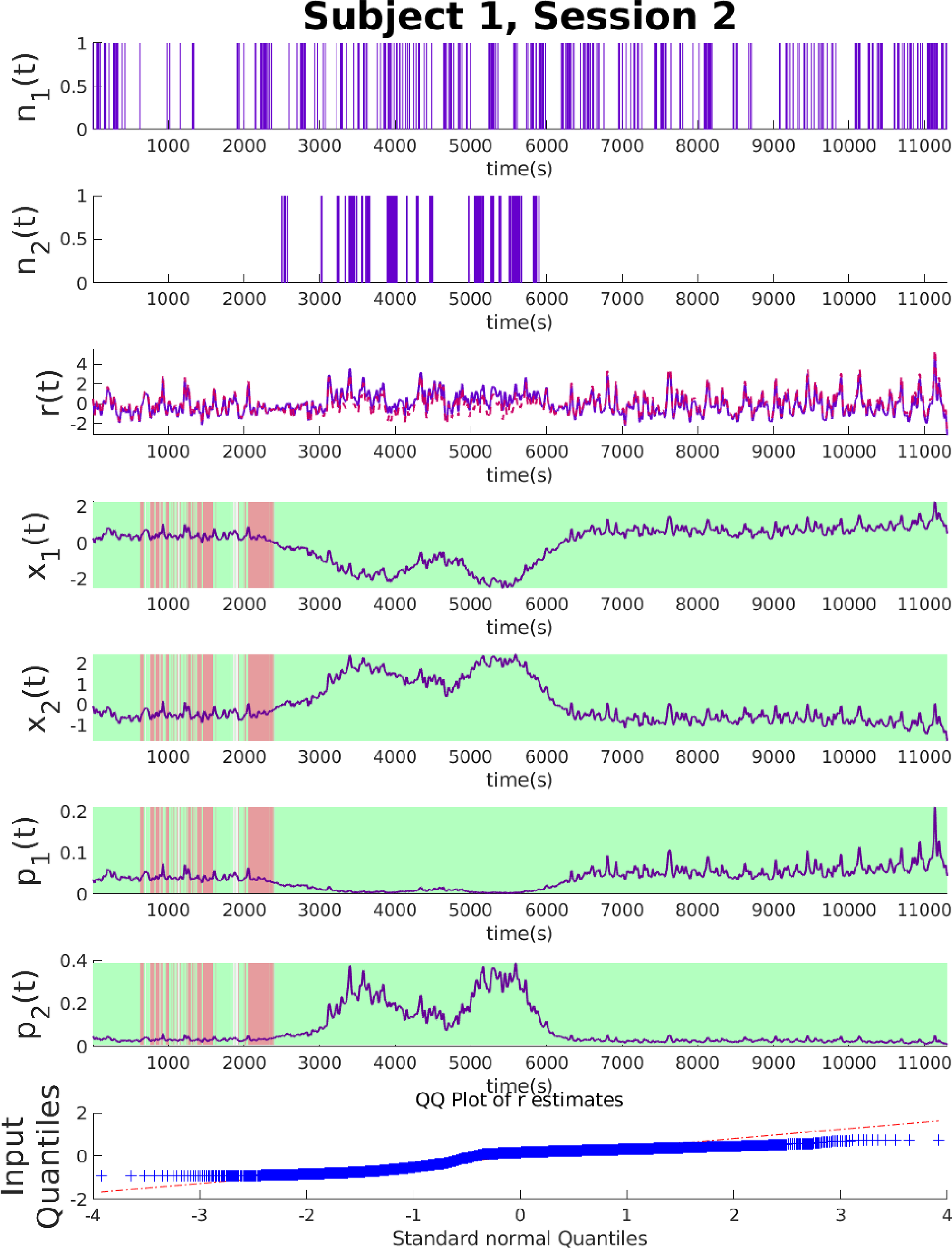

Supplement: S2 Fig — The panel shows the experimental data with time limit. From top, the binary variables n1 and n2 derived from deconvolved EDA data and typing data respectively, the continuous variable r denoting the RR intervals derived from heart rate (red line) and r˜ estimated from latent variables x1 and x2 (purple line), x1 and x2 in order from top indicating cognitive arousal state and expressive typing state respectively. p1 and p2 show the estimated probabilities. Patches of green, red, and cyan indicate what application the subject was using at the time of measurement. Green indicates applications for information search like internet explorer, red is for typing like Microsoft word and PowerPoint and cyan is for when subjects are looking at their emails. Finally, the QQ plot for the residual error of r is shown. (TIF) [file pone.0300786.s003.tif]

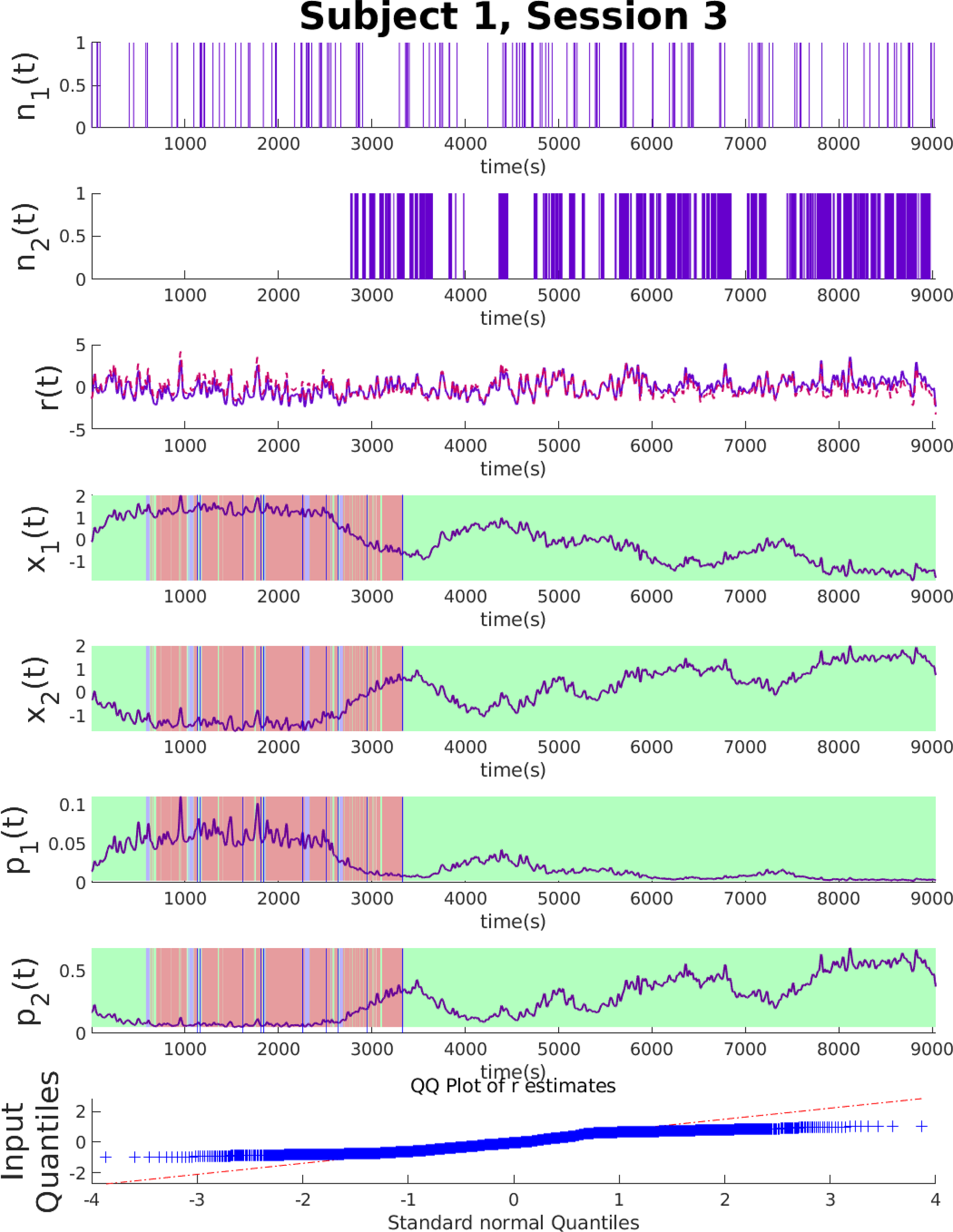

Supplement: S3 Fig — The panel shows the experimental data with interruptions. From top, the binary variables n1 and n2 derived from deconvolved EDA data and typing data respectively, the continuous variable r denoting the RR intervals derived from heart rate (red line) and r˜ estimated from latent variables x1 and x2 (purple line), x1 and x2 in order from top indicating cognitive arousal state and expressive typing state respectively. p1 and p2 show the estimated probabilities. Patches of green, red, and cyan indicate what application the subject was using at the time of measurement. Green indicates applications for information search like internet explorer, red is for typing like Microsoft word and PowerPoint and cyan is for when subjects are looking at their emails. The Blue vertical line indicates the time email notifications were sent. Finally, the QQ plot for the residual error of r is shown. (TIF) [file pone.0300786.s004.tif]

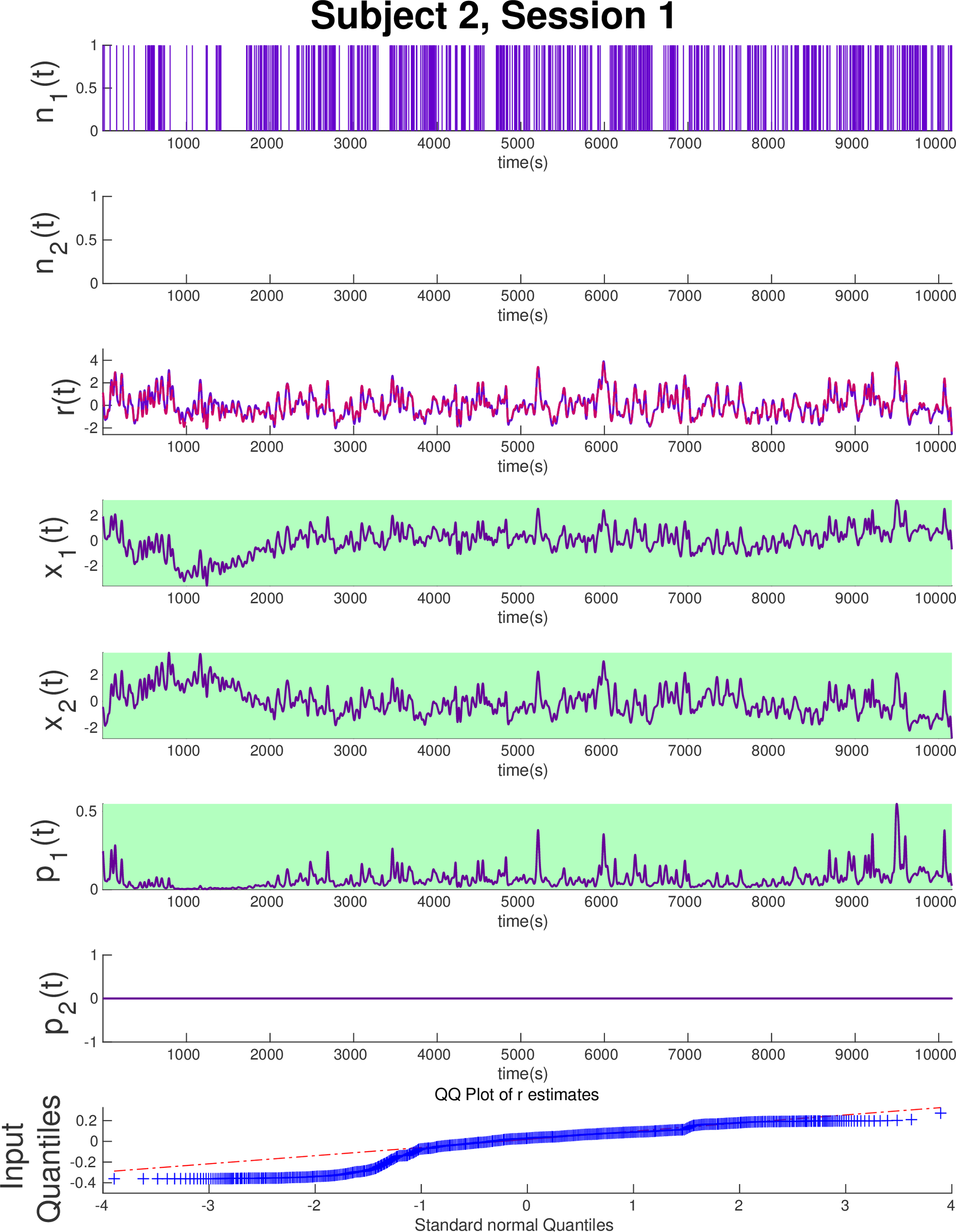

Supplement: S4 Fig — The panel shows the experimental data for no stressor sessions. From top, the binary variables n1 and n2 derived from deconvolved EDA data and typing data respectively, the continuous variable r denoting the RR intervals derived from heart rate (red line) and r˜ estimated from latent variables x1 and x2 (purple line), x1 and x2 in order from top indicating cognitive arousal state and expressive typing state respectively. p1 and p2 show the estimated probabilities. Patches of green, red, and cyan indicate what application the subject was using at the time of measurement. Green indicates applications for information search like internet explorer, red is for typing like Microsoft word and PowerPoint and cyan is for when subjects are looking at their emails. Finally, the QQ plot for the residual error of r is shown. (TIF) [file pone.0300786.s005.tif]

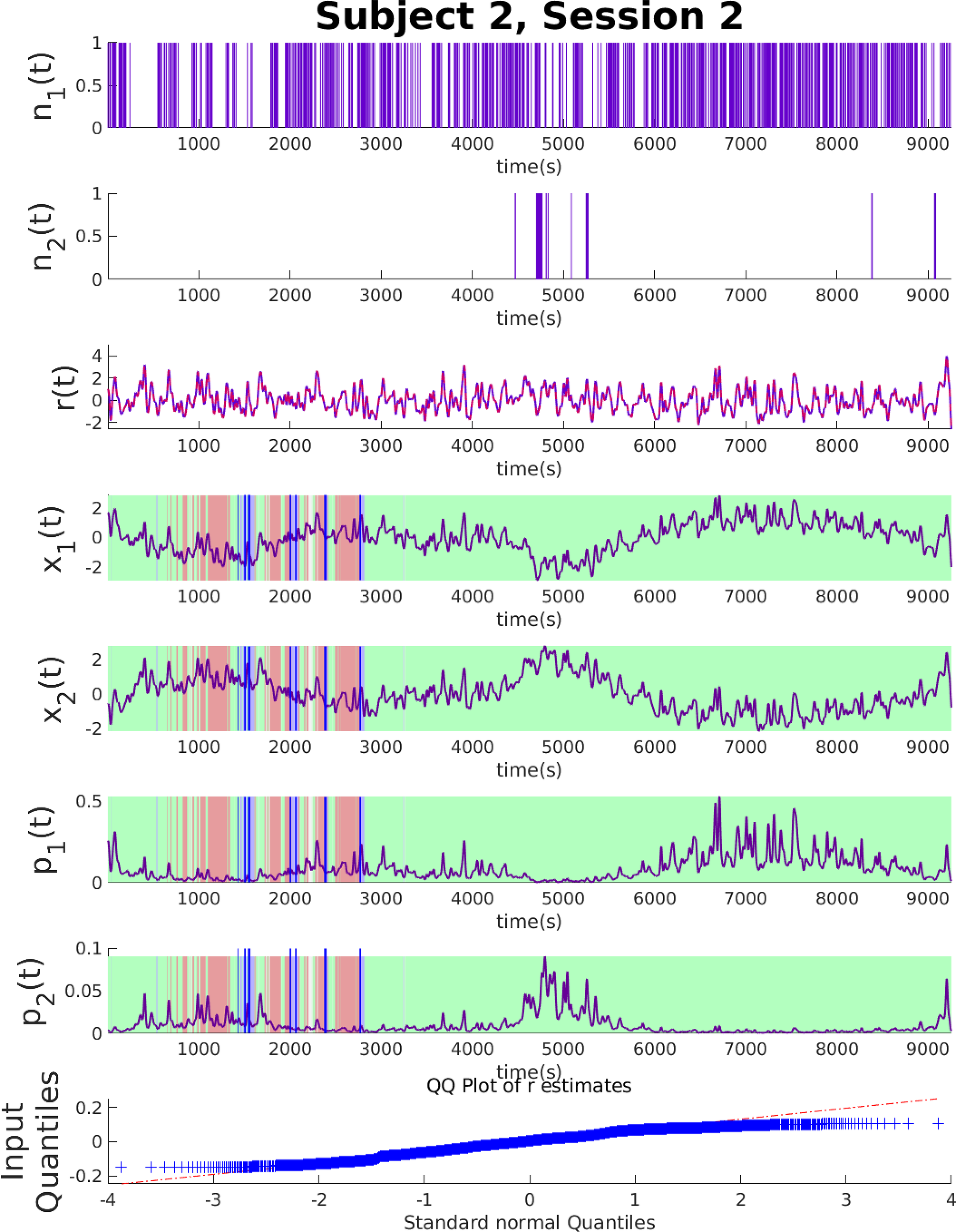

Supplement: S5 Fig — The panel shows the experimental data with time limit. From top, the binary variables n1 and n2 derived from deconvolved EDA data and typing data respectively, the continuous variable r denoting the RR intervals derived from heart rate (red line) and r˜ estimated from latent variables x1 and x2 (purple line), x1 and x2 in order from top indicating cognitive arousal state and expressive typing state respectively. p1 and p2 show the estimated probabilities. Patches of green, red, and cyan indicate what application the subject was using at the time of measurement. Green indicates applications for information search like internet explorer, red is for typing like Microsoft word and PowerPoint and cyan is for when subjects are looking at their emails. Finally, the QQ plot for the residual error of r is shown. (TIF) [file pone.0300786.s006.tif]

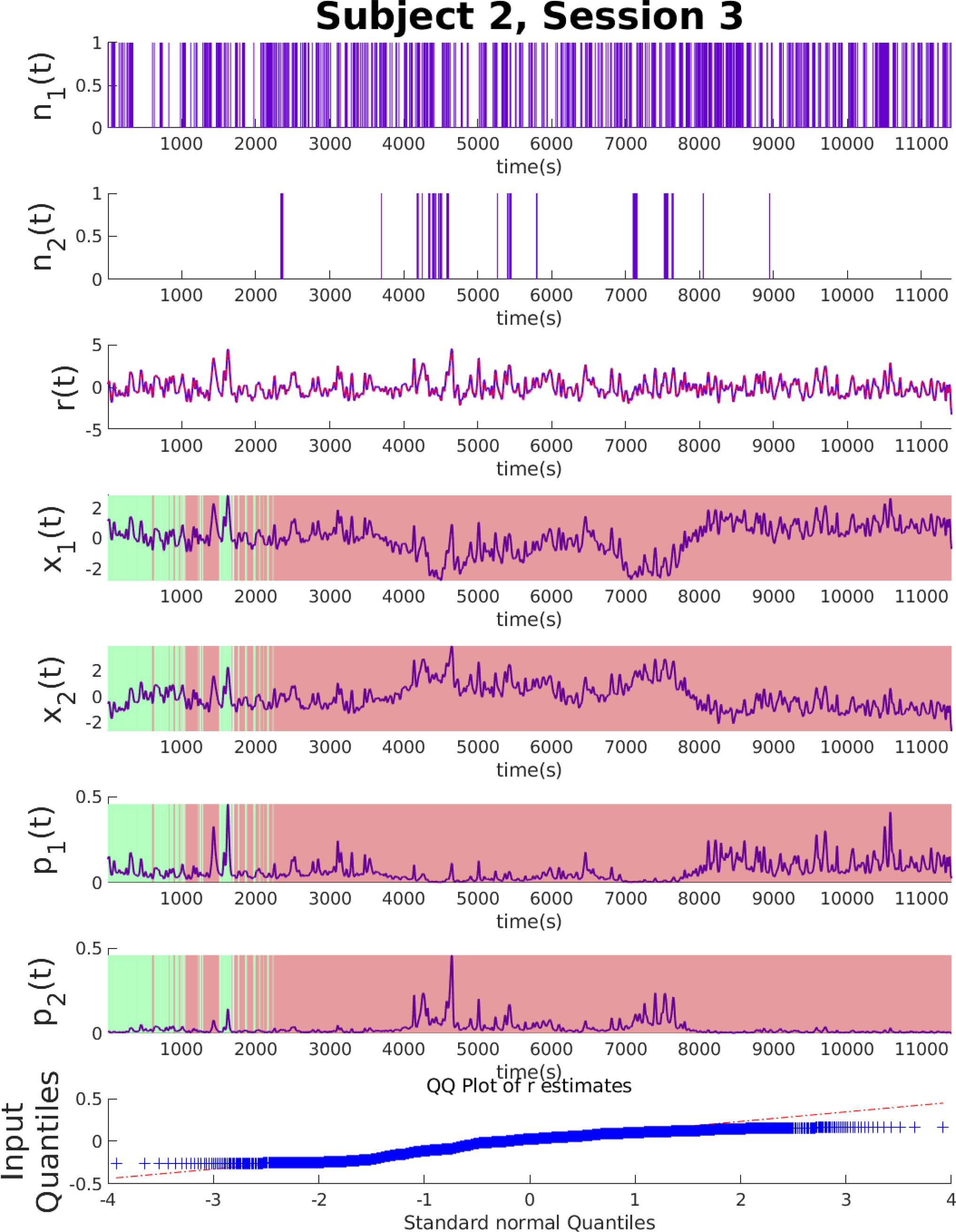

Supplement: S6 Fig — The panel shows the experimental data with interruptions. From top, the binary variables n1 and n2 derived from deconvolved EDA data and typing data respectively, the continuous variable r denoting the RR intervals derived from heart rate (red line) and r˜ estimated from latent variables x1 and x2 (purple line), x1 and x2 in order from top indicating cognitive arousal state and expressive typing state respectively. p1 and p2 show the estimated probabilities. Patches of green, red, and cyan indicate what application the subject was using at the time of measurement. Green indicates applications for information search like internet explorer, red is for typing like Microsoft word and PowerPoint and cyan is for when subjects are looking at their emails. The Blue vertical line indicates the time email notifications were sent. Finally, the QQ plot for the residual error of r is shown. (TIF) [file pone.0300786.s007.tif]

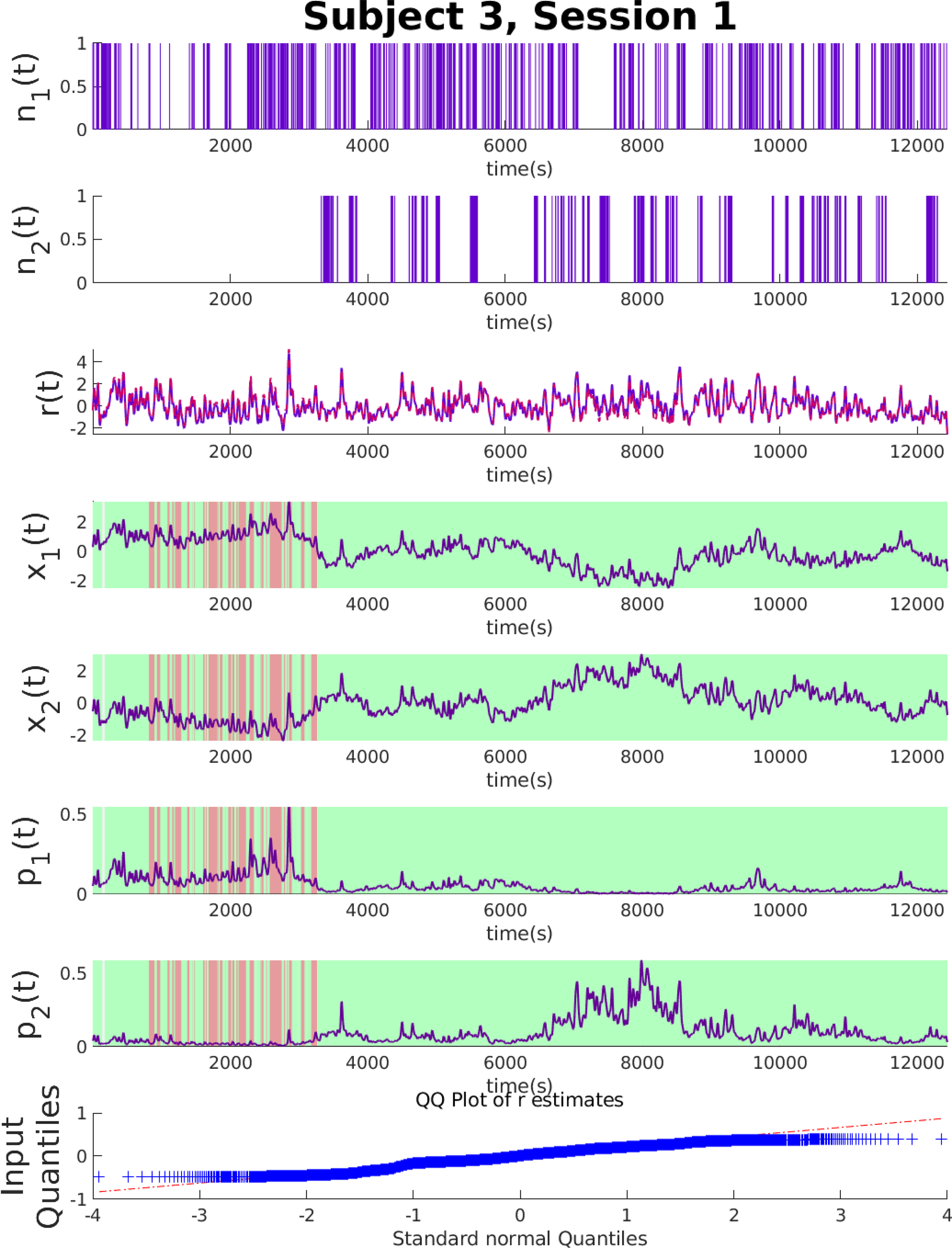

Supplement: S7 Fig — The panel shows the experimental data for no stressor sessions. From top, the binary variables n1 and n2 derived from deconvolved EDA data and typing data respectively, the continuous variable r denoting the RR intervals derived from heart rate (red line) and r˜ estimated from latent variables x1 and x2 (purple line), x1 and x2 in order from top indicating cognitive arousal state and expressive typing state respectively. p1 and p2 show the estimated probabilities. Patches of green, red, and cyan indicate what application the subject was using at the time of measurement. Green indicates applications for information search like internet explorer, red is for typing like Microsoft word and PowerPoint and cyan is for when subjects are looking at their emails. Finally, the QQ plot for the residual error of r is shown. (TIF) [file pone.0300786.s008.tif]

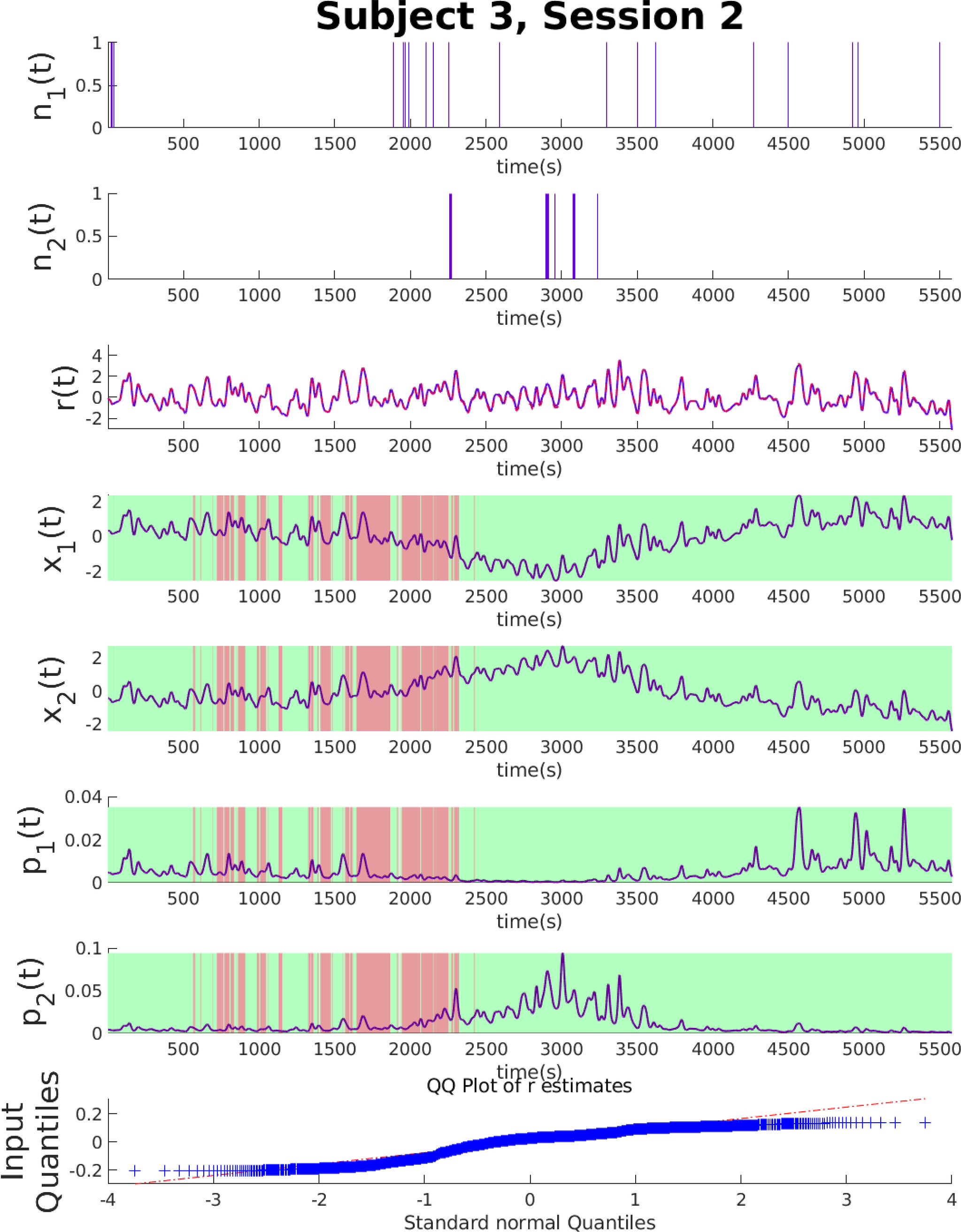

Supplement: S8 Fig — The panel shows the experimental data with time limit. From top, the binary variables n1 and n2 derived from deconvolved EDA data and typing data respectively, the continuous variable r denoting the RR intervals derived from heart rate (red line) and r˜ estimated from latent variables x1 and x2 (purple line), x1 and x2 in order from top indicating cognitive arousal state and expressive typing state respectively. p1 and p2 show the estimated probabilities. Patches of green, red, and cyan indicate what application the subject was using at the time of measurement. Green indicates applications for information search like internet explorer, red is for typing like Microsoft word and PowerPoint and cyan is for when subjects are looking at their emails. Finally, the QQ plot for the residual error of r is shown. (TIF) [file pone.0300786.s009.tif]

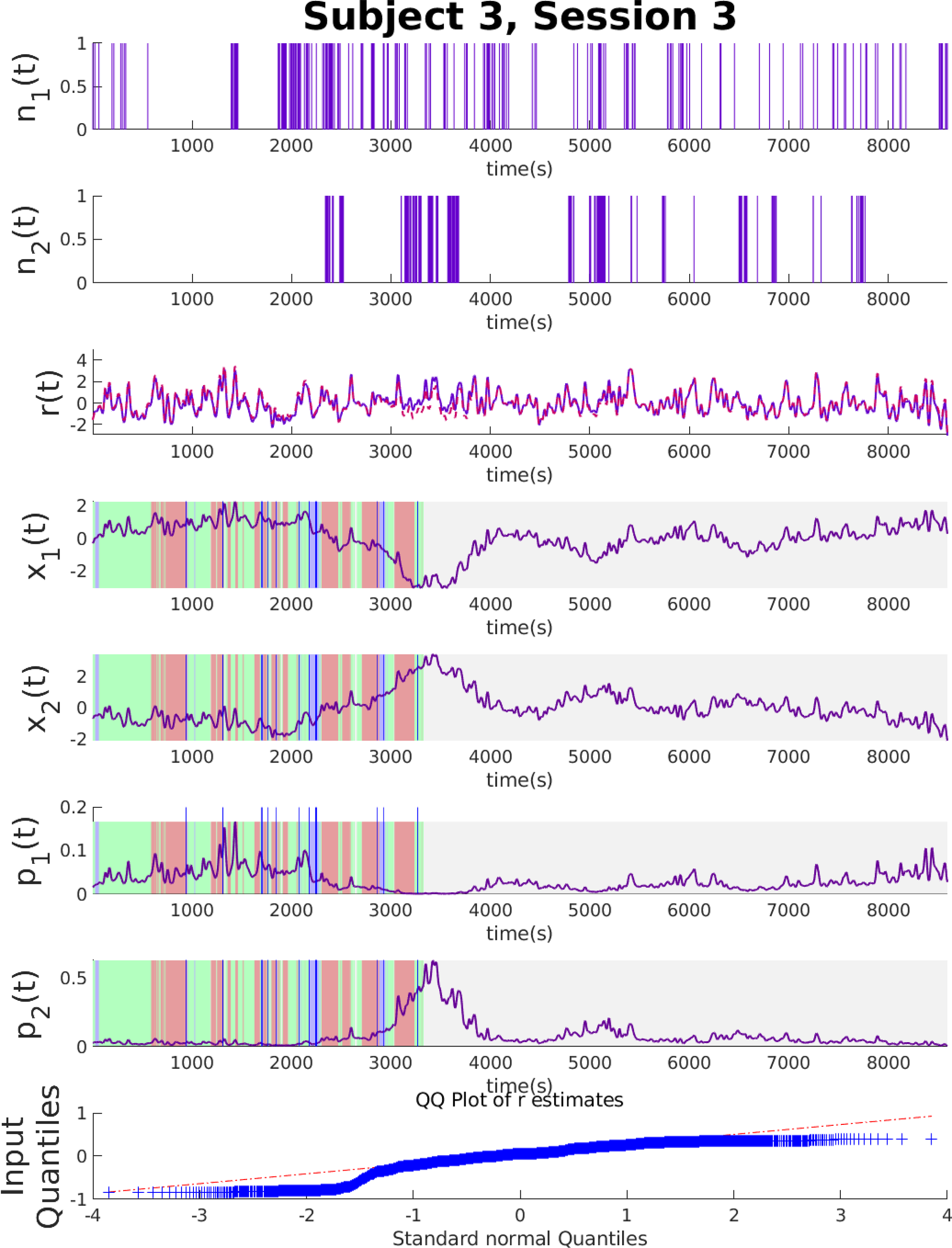

Supplement: S9 Fig — The panel shows the experimental data with interruptions. From top, the binary variables n1 and n2 derived from deconvolved EDA data and typing data respectively, the continuous variable r denoting the RR intervals derived from heart rate (red line) and r˜ estimated from latent variables x1 and x2 (purple line), x1 and x2 in order from top indicating cognitive arousal state and expressive typing state respectively. p1 and p2 show the estimated probabilities. Patches of green, red, and cyan indicate what application the subject was using at the time of measurement. Green indicates applications for information search like internet explorer, red is for typing like Microsoft word and PowerPoint and cyan is for when subjects are looking at their emails. The Blue vertical line indicates the time email notifications were sent. Finally, the QQ plot for the residual error of r is shown. (TIF) [file pone.0300786.s010.tif]

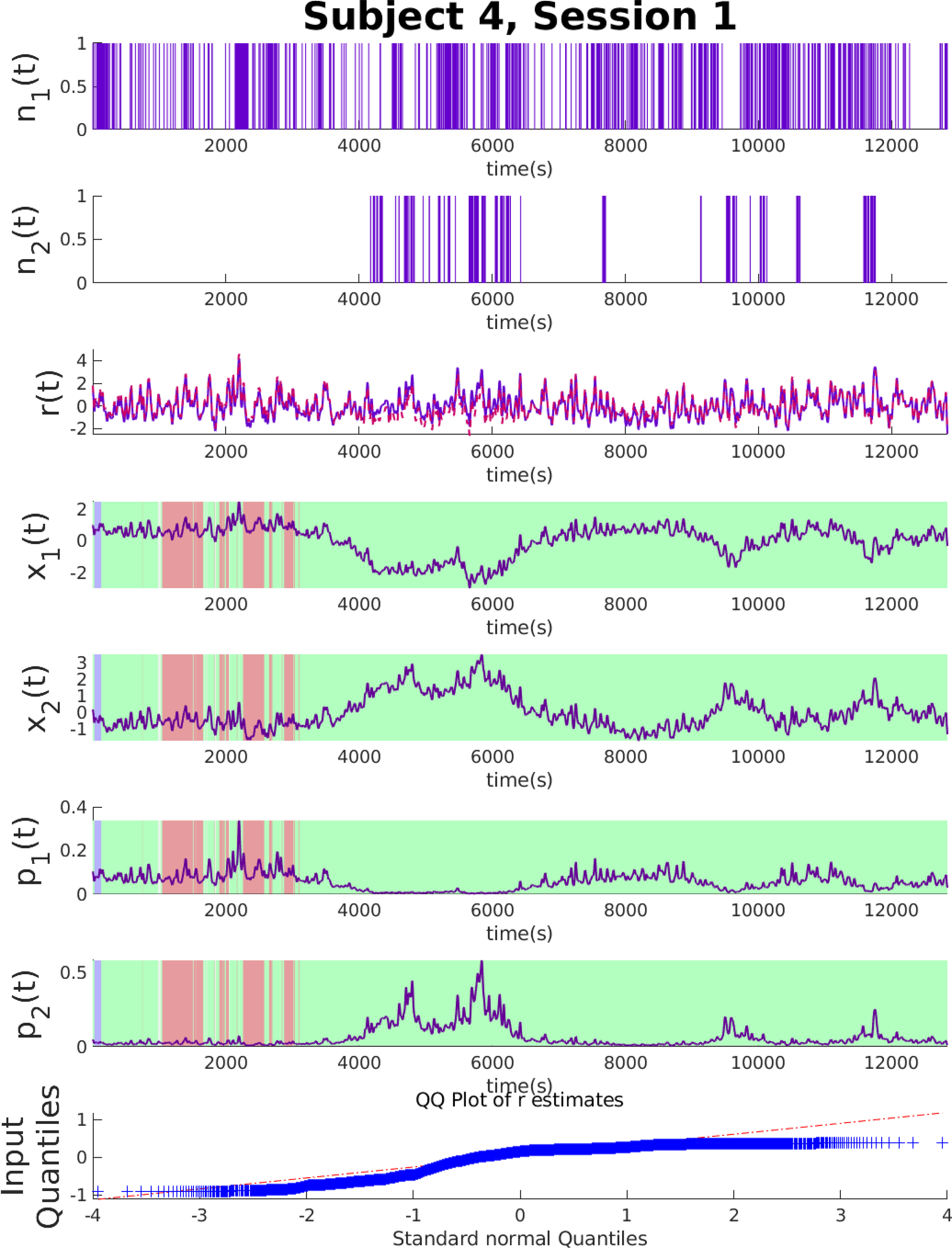

Supplement: S10 Fig — The panel shows the experimental data for no stressor sessions. From top, the binary variables n1 and n2 derived from deconvolved EDA data and typing data respectively, the continuous variable r denoting the RR intervals derived from heart rate (red line) and r˜ estimated from latent variables x1 and x2 (purple line), x1 and x2 in order from top indicating cognitive arousal state and expressive typing state respectively. p1 and p2 show the estimated probabilities. Patches of green, red, and cyan indicate what application the subject was using at the time of measurement. Green indicates applications for information search like internet explorer, red is for typing like Microsoft word and PowerPoint and cyan is for when subjects are looking at their emails. Finally, the QQ plot for the residual error of r is shown. (TIF) [file pone.0300786.s011.tif]

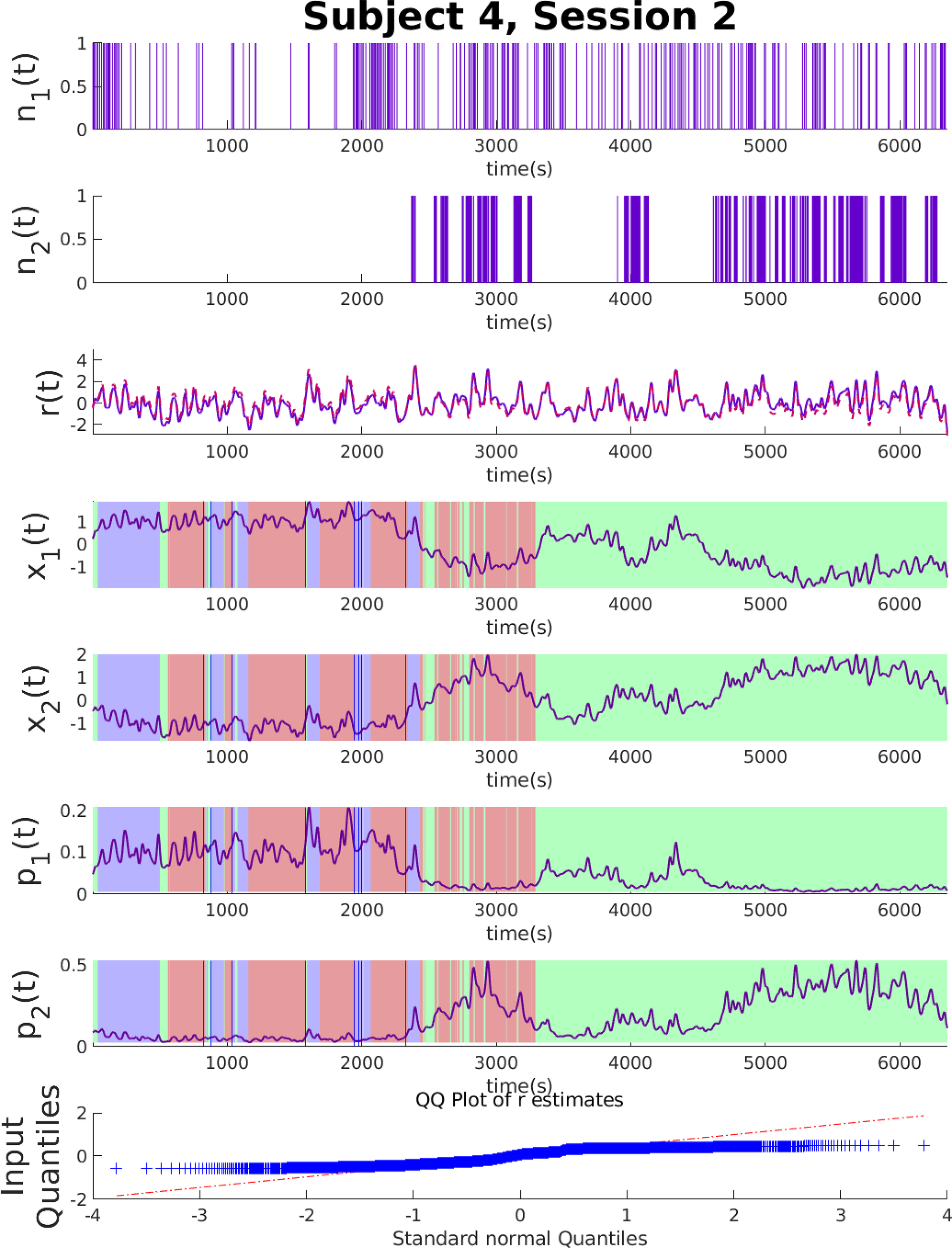

Supplement: S11 Fig — The panel shows the experimental data with time limit. From top, the binary variables n1 and n2 derived from deconvolved EDA data and typing data respectively, the continuous variable r denoting the RR intervals derived from heart rate (red line) and r˜ estimated from latent variables x1 and x2 (purple line), x1 and x2 in order from top indicating cognitive arousal state and expressive typing state respectively. p1 and p2 show the estimated probabilities. Patches of green, red, and cyan indicate what application the subject was using at the time of measurement. Green indicates applications for information search like internet explorer, red is for typing like Microsoft word and PowerPoint and cyan is for when subjects are looking at their emails. Finally, the QQ plot for the residual error of r is shown. (TIF) [file pone.0300786.s012.tif]

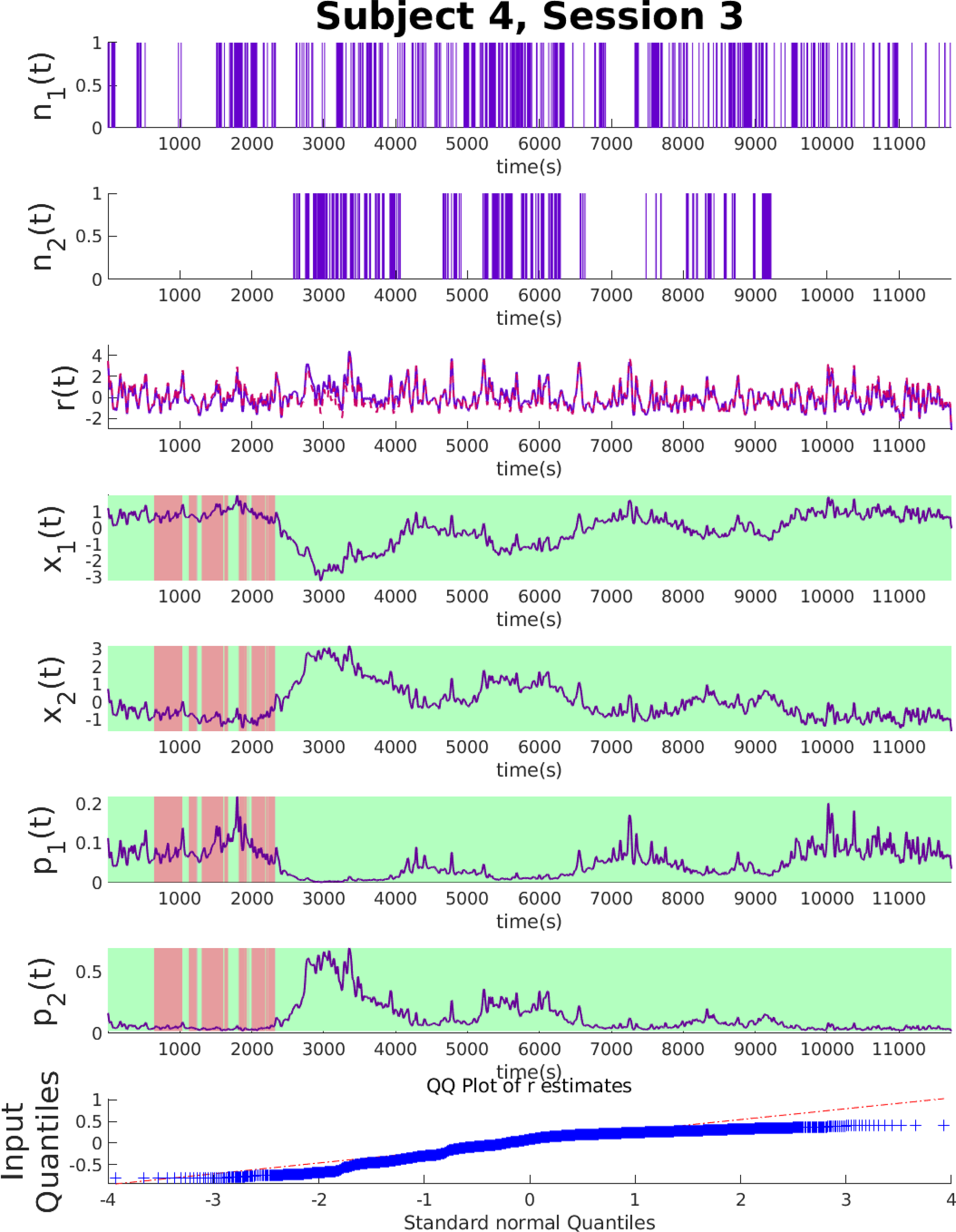

Supplement: S12 Fig — The panel shows the experimental data with interruptions. From top, the binary variables n1 and n2 derived from deconvolved EDA data and typing data respectively, the continuous variable r denoting the RR intervals derived from heart rate (red line) and r˜ estimated from latent variables x1 and x2 (purple line), x1 and x2 in order from top indicating cognitive arousal state and expressive typing state respectively. p1 and p2 show the estimated probabilities. Patches of green, red, and cyan indicate what application the subject was using at the time of measurement. Green indicates applications for information search like internet explorer, red is for typing like Microsoft word and PowerPoint and cyan is for when subjects are looking at their emails. The Blue vertical line indicates the time email notifications were sent. Finally, the QQ plot for the residual error of r is shown. (TIF) [file pone.0300786.s013.tif]

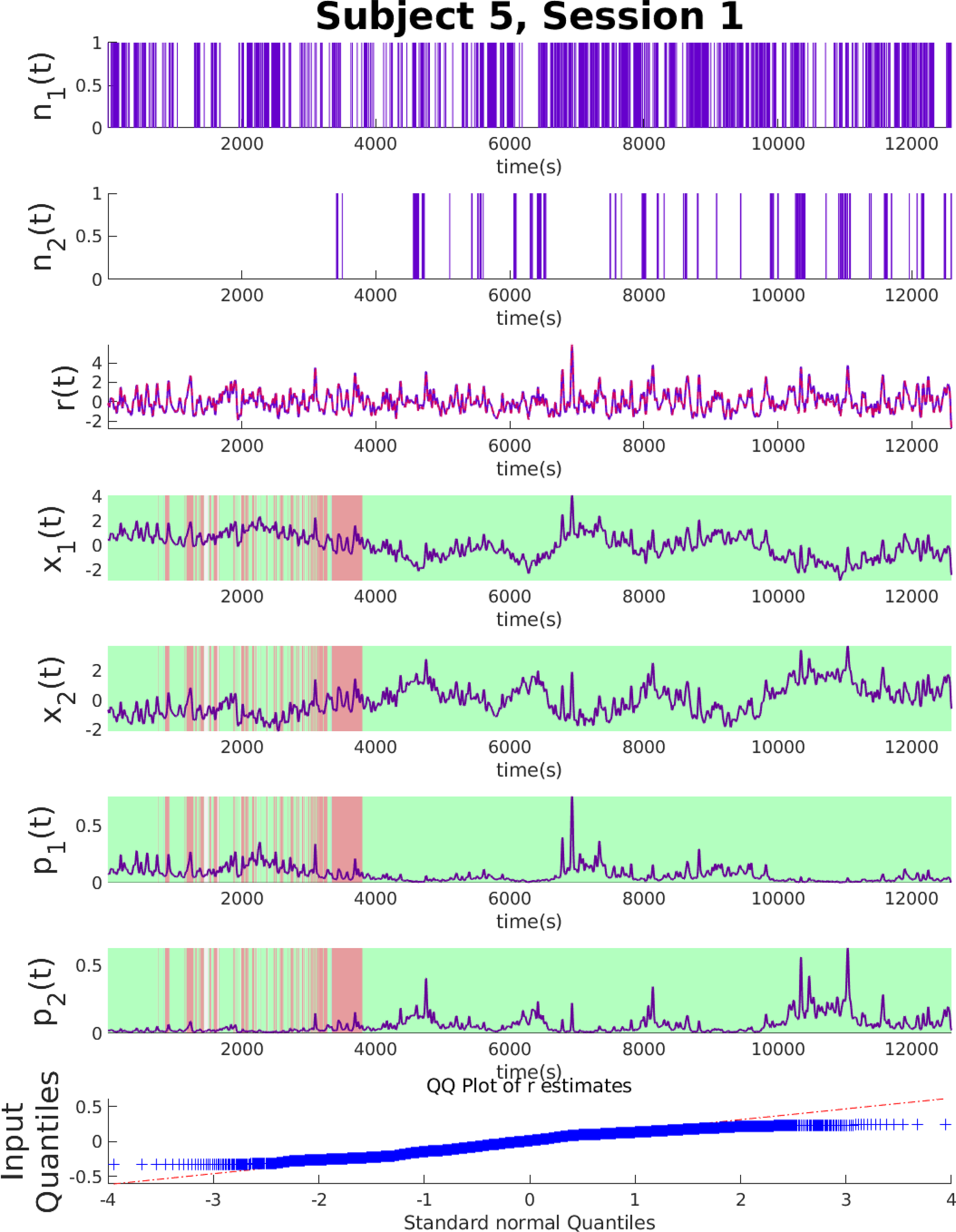

Supplement: S13 Fig — The panel shows the experimental data for no stressor sessions. From top, the binary variables n1 and n2 derived from deconvolved EDA data and typing data respectively, the continuous variable r denoting the RR intervals derived from heart rate (red line) and r˜ estimated from latent variables x1 and x2 (purple line), x1 and x2 in order from top indicating cognitive arousal state and expressive typing state respectively. p1 and p2 show the estimated probabilities. Patches of green, red, and cyan indicate what application the subject was using at the time of measurement. Green indicates applications for information search like internet explorer, red is for typing like Microsoft word and PowerPoint and cyan is for when subjects are looking at their emails. Finally, the QQ plot for the residual error of r is shown. (TIF) [file pone.0300786.s014.tif]

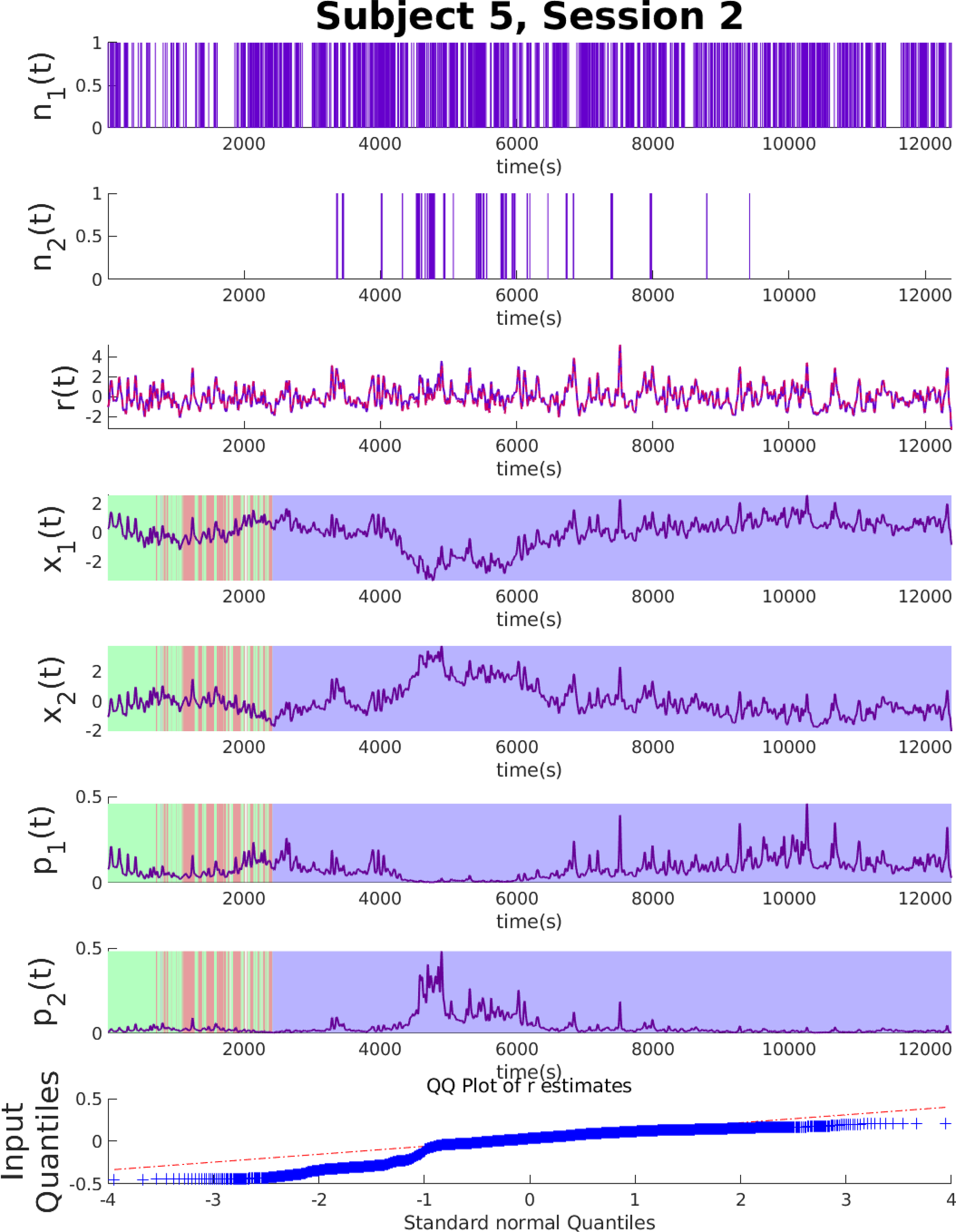

Supplement: S14 Fig — The panel shows the experimental data with time limit. From top, the binary variables n1 and n2 derived from deconvolved EDA data and typing data respectively, the continuous variable r denoting the RR intervals derived from heart rate (red line) and r˜ estimated from latent variables x1 and x2 (purple line), x1 and x2 in order from top indicating cognitive arousal state and expressive typing state respectively. p1 and p2 show the estimated probabilities. Patches of green, red, and cyan indicate what application the subject was using at the time of measurement. Green indicates applications for information search like internet explorer, red is for typing like Microsoft word and PowerPoint and cyan is for when subjects are looking at their emails. Finally, the QQ plot for the residual error of r is shown. (TIF) [file pone.0300786.s015.tif]

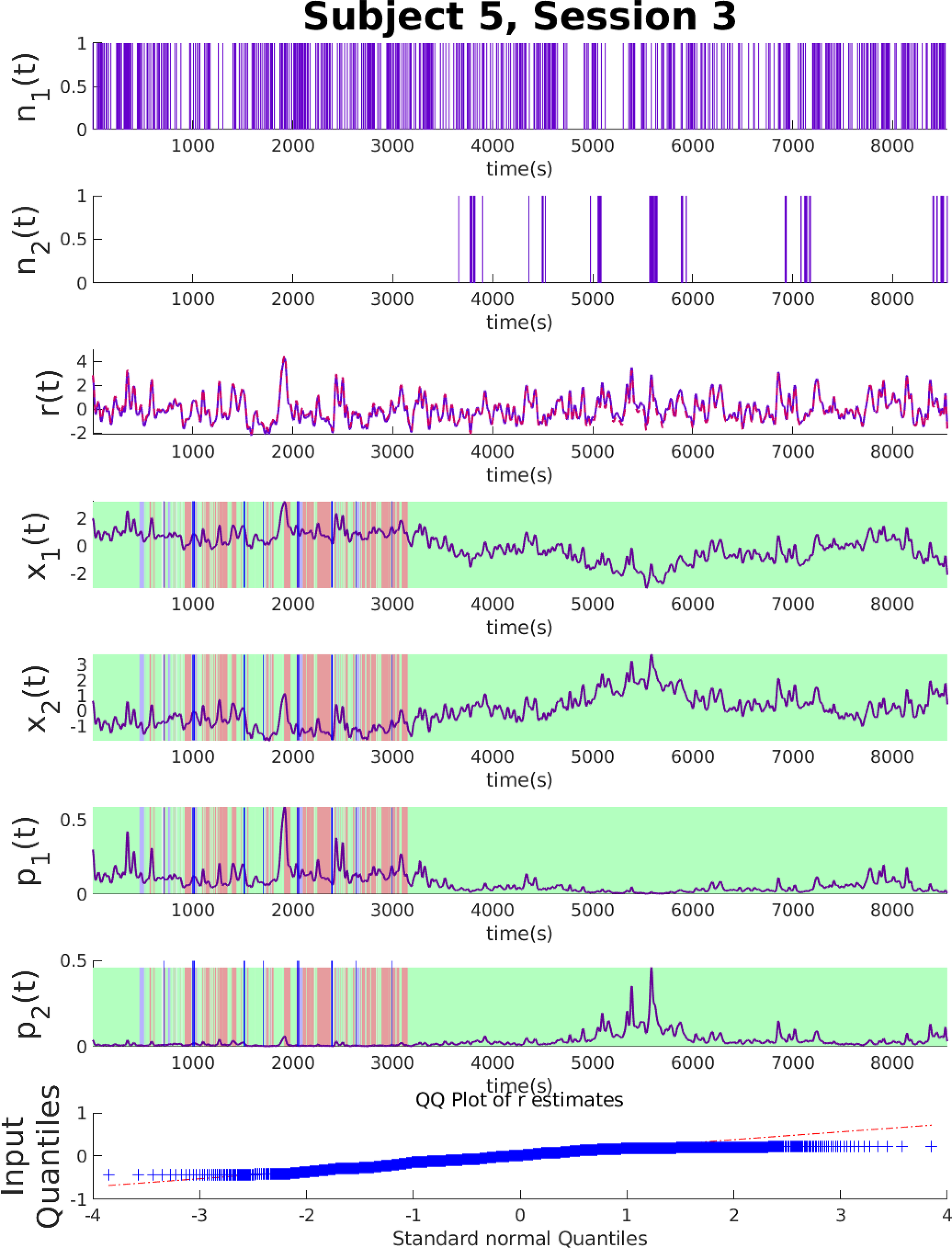

Supplement: S15 Fig — The panel shows the experimental data with interruptions. From top, the binary variables n1 and n2 derived from deconvolved EDA data and typing data respectively, the continuous variable r denoting the RR intervals derived from heart rate (red line) and r˜ estimated from latent variables x1 and x2 (purple line), x1 and x2 in order from top indicating cognitive arousal state and expressive typing state respectively. p1 and p2 show the estimated probabilities. Patches of green, red, and cyan indicate what application the subject was using at the time of measurement. Green indicates applications for information search like internet explorer, red is for typing like Microsoft word and PowerPoint and cyan is for when subjects are looking at their emails. The Blue vertical line indicates the time email notifications were sent. Finally, the QQ plot for the residual error of r is shown. (TIF) [file pone.0300786.s016.tif]

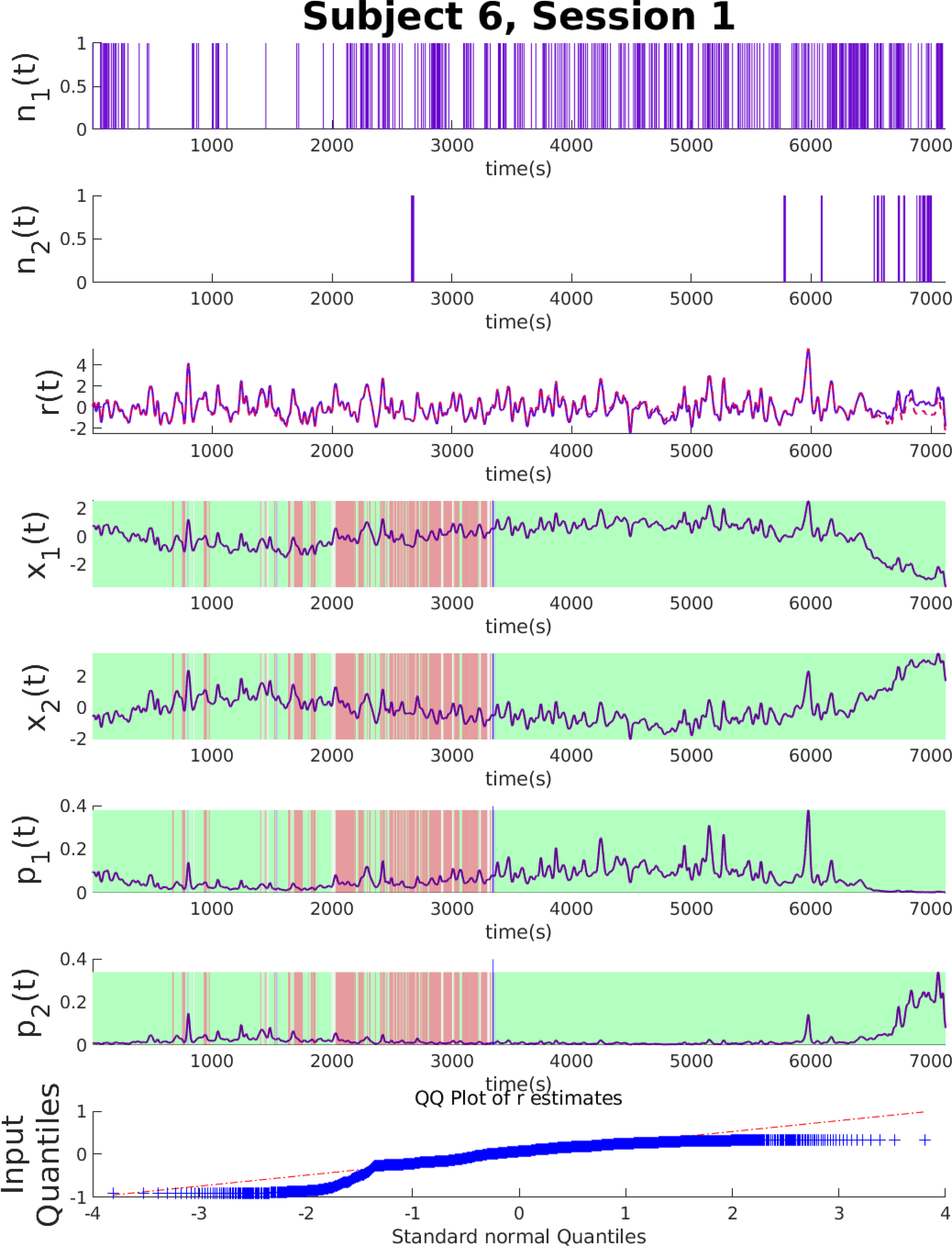

Supplement: S16 Fig — The panel shows the experimental data for no stressor sessions. From top, the binary variables n1 and n2 derived from deconvolved EDA data and typing data respectively, the continuous variable r denoting the RR intervals derived from heart rate (red line) and r˜ estimated from latent variables x1 and x2 (purple line), x1 and x2 in order from top indicating cognitive arousal state and expressive typing state respectively. p1 and p2 show the estimated probabilities. Patches of green, red, and cyan indicate what application the subject was using at the time of measurement. Green indicates applications for information search like internet explorer, red is for typing like Microsoft word and PowerPoint and cyan is for when subjects are looking at their emails. Finally, the QQ plot for the residual error of r is shown. (TIF) [file pone.0300786.s017.tif]

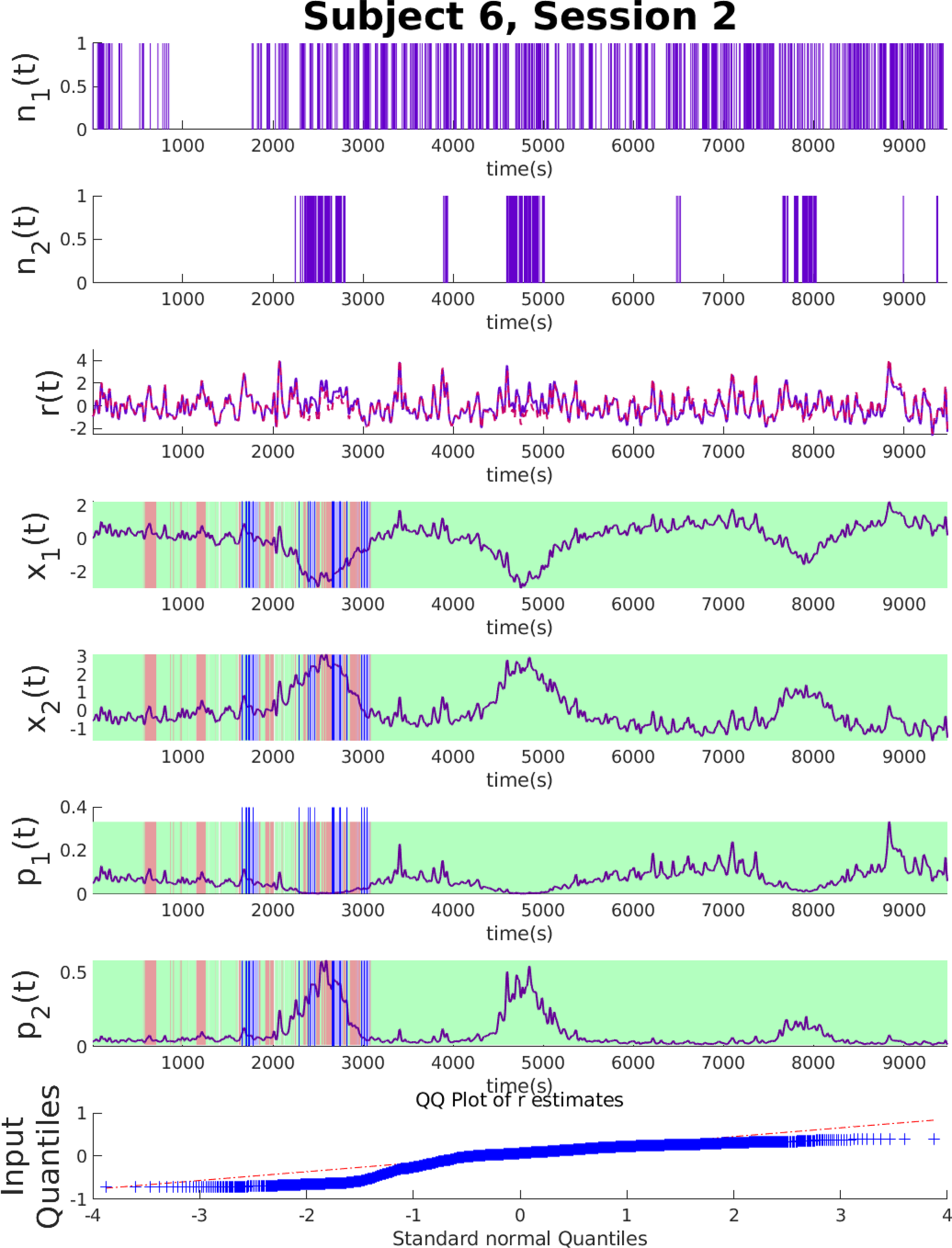

Supplement: S17 Fig — The panel shows the experimental data with time limit. From top, the binary variables n1 and n2 derived from deconvolved EDA data and typing data respectively, the continuous variable r denoting the RR intervals derived from heart rate (red line) and r˜ estimated from latent variables x1 and x2 (purple line), x1 and x2 in order from top indicating cognitive arousal state and expressive typing state respectively. p1 and p2 show the estimated probabilities. Patches of green, red, and cyan indicate what application the subject was using at the time of measurement. Green indicates applications for information search like internet explorer, red is for typing like Microsoft word and PowerPoint and cyan is for when subjects are looking at their emails. Finally, the QQ plot for the residual error of r is shown. (TIF) [file pone.0300786.s018.tif]

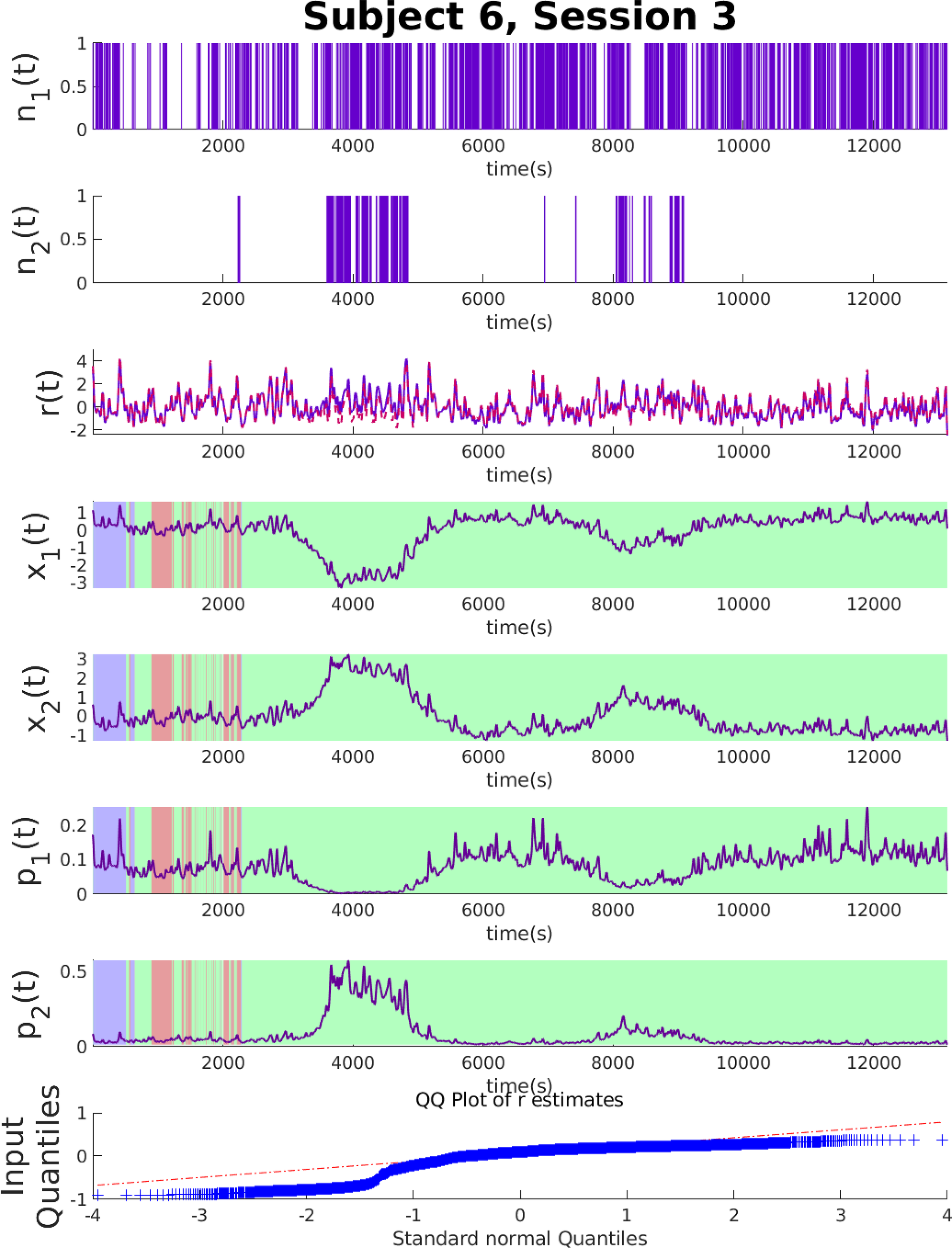

Supplement: S18 Fig — The panel shows the experimental data with interruptions. From top, the binary variables n1 and n2 derived from deconvolved EDA data and typing data respectively, the continuous variable r denoting the RR intervals derived from heart rate (red line) and r˜ estimated from latent variables x1 and x2 (purple line), x1 and x2 in order from top indicating cognitive arousal state and expressive typing state respectively. p1 and p2 show the estimated probabilities. Patches of green, red, and cyan indicate what application the subject was using at the time of measurement. Green indicates applications for information search like internet explorer, red is for typing like Microsoft word and PowerPoint and cyan is for when subjects are looking at their emails. The Blue vertical line indicates the time email notifications were sent. Finally, the QQ plot for the residual error of r is shown. (TIF) [file pone.0300786.s019.tif]

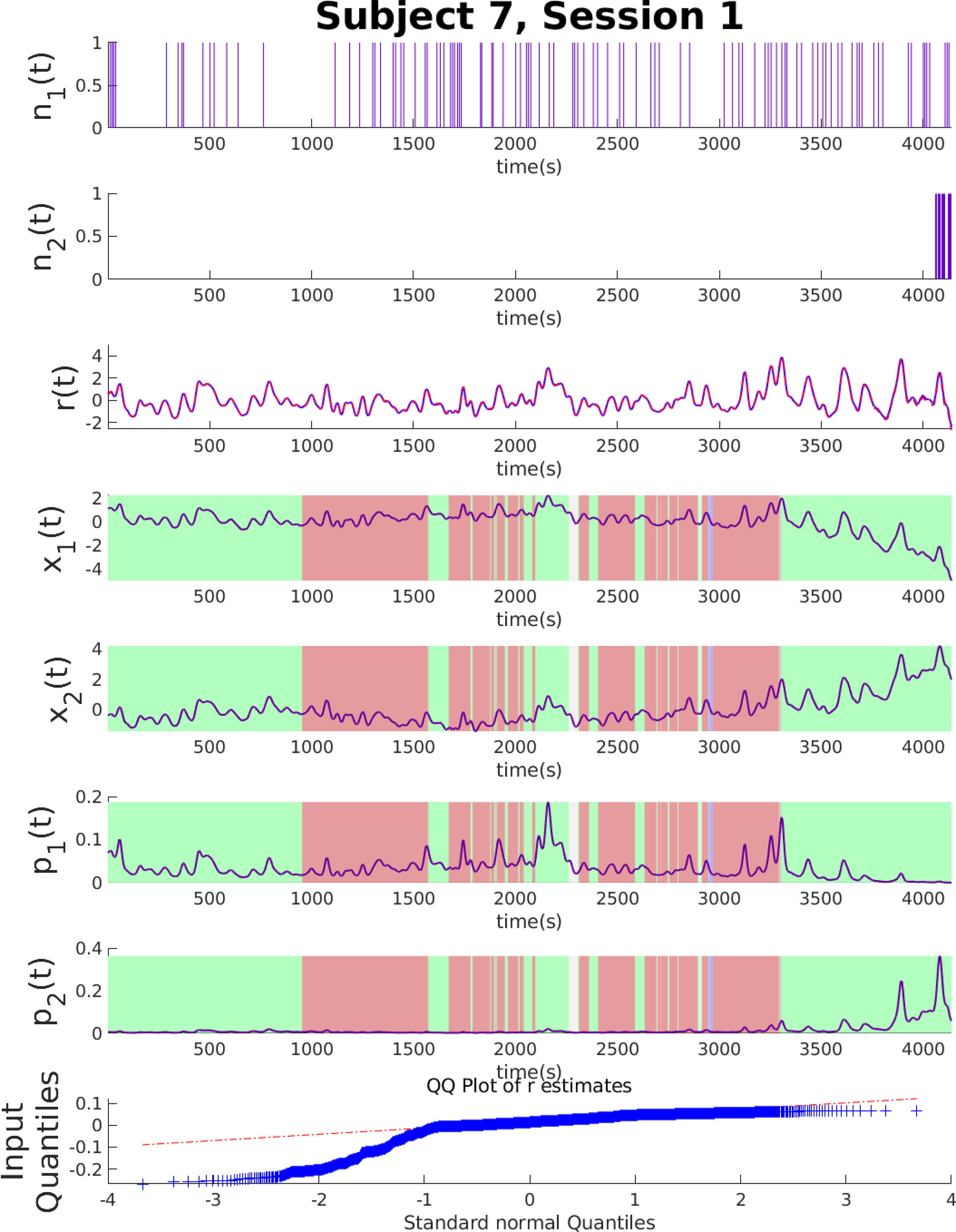

Supplement: S19 Fig — The panel shows the experimental data for no stressor sessions. From top, the binary variables n1 and n2 derived from deconvolved EDA data and typing data respectively, the continuous variable r denoting the RR intervals derived from heart rate (red line) and r˜ estimated from latent variables x1 and x2 (purple line), x1 and x2 in order from top indicating cognitive arousal state and expressive typing state respectively. p1 and p2 show the estimated probabilities. Patches of green, red, and cyan indicate what application the subject was using at the time of measurement. Green indicates applications for information search like internet explorer, red is for typing like Microsoft word and PowerPoint and cyan is for when subjects are looking at their emails. Finally, the QQ plot for the residual error of r is shown. (TIF) [file pone.0300786.s020.tif]

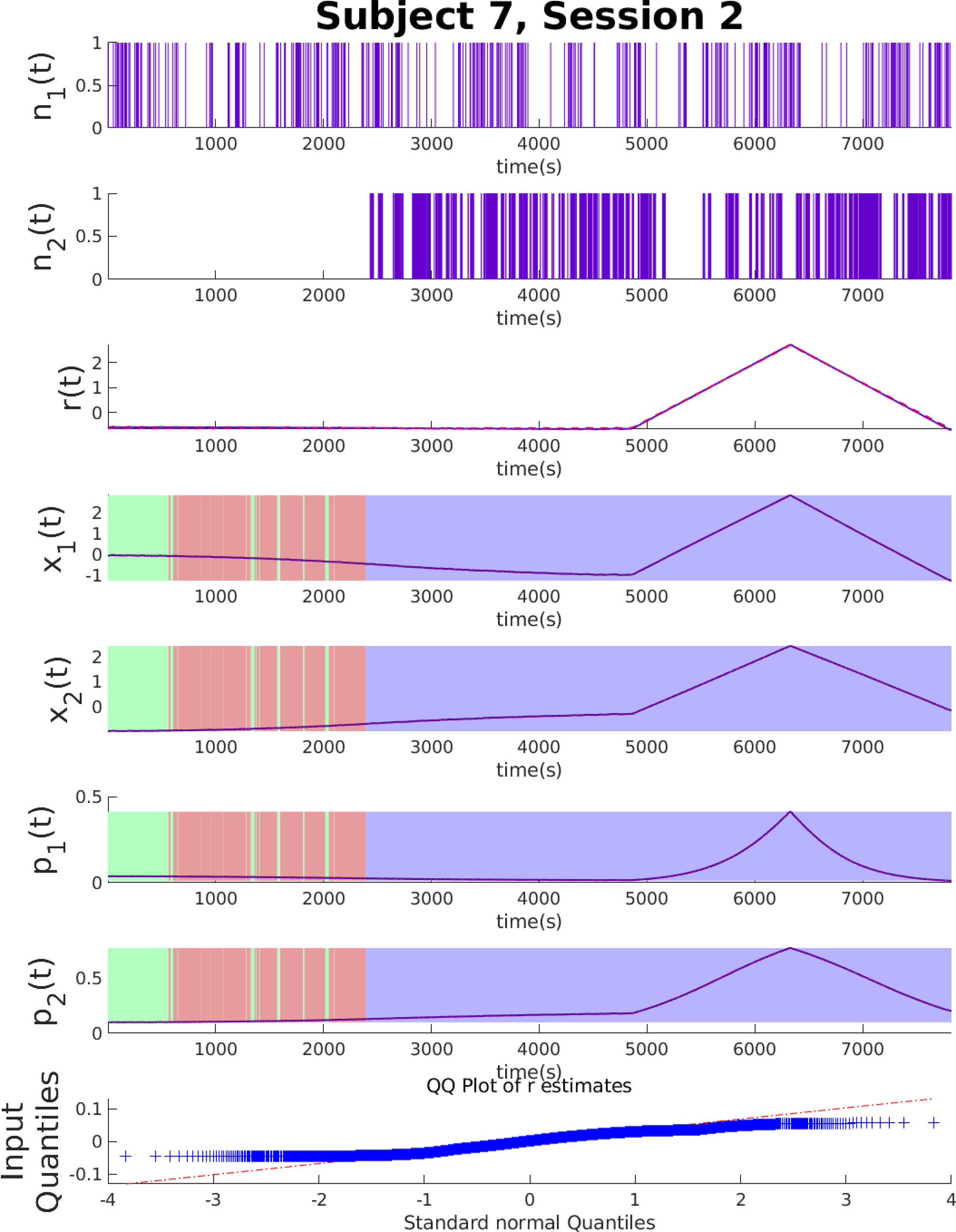

Supplement: S20 Fig — The panel shows the experimental data with time limit. From top, the binary variables n1 and n2 derived from deconvolved EDA data and typing data respectively, the continuous variable r denoting the RR intervals derived from heart rate (red line) and r˜ estimated from latent variables x1 and x2 (purple line), x1 and x2 in order from top indicating cognitive arousal state and expressive typing state respectively. p1 and p2 show the estimated probabilities. Patches of green, red, and cyan indicate what application the subject was using at the time of measurement. Green indicates applications for information search like internet explorer, red is for typing like Microsoft word and PowerPoint and cyan is for when subjects are looking at their emails. Finally, the QQ plot for the residual error of r is shown. (TIF) [file pone.0300786.s021.tif]

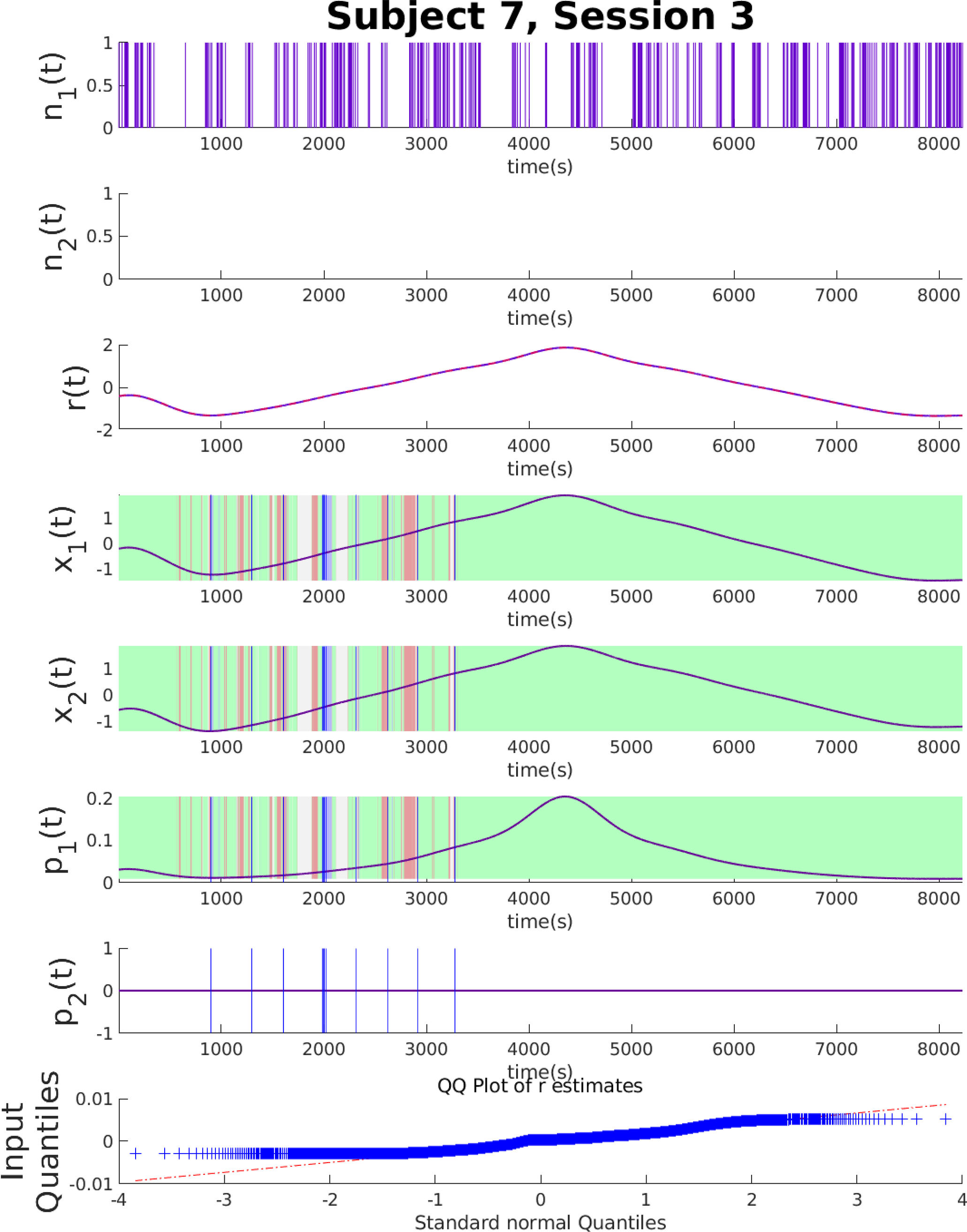

Supplement: S21 Fig — The panel shows the experimental data with interruptions. From top, the binary variables n1 and n2 derived from deconvolved EDA data and typing data respectively, the continuous variable r denoting the RR intervals derived from heart rate (red line) and r˜ estimated from latent variables x1 and x2 (purple line), x1 and x2 in order from top indicating cognitive arousal state and expressive typing state respectively. p1 and p2 show the estimated probabilities. Patches of green, red, and cyan indicate what application the subject was using at the time of measurement. Green indicates applications for information search like internet explorer, red is for typing like Microsoft word and PowerPoint and cyan is for when subjects are looking at their emails. The Blue vertical line indicates the time email notifications were sent. Finally, the QQ plot for the residual error of r is shown. (TIF) [file pone.0300786.s022.tif]

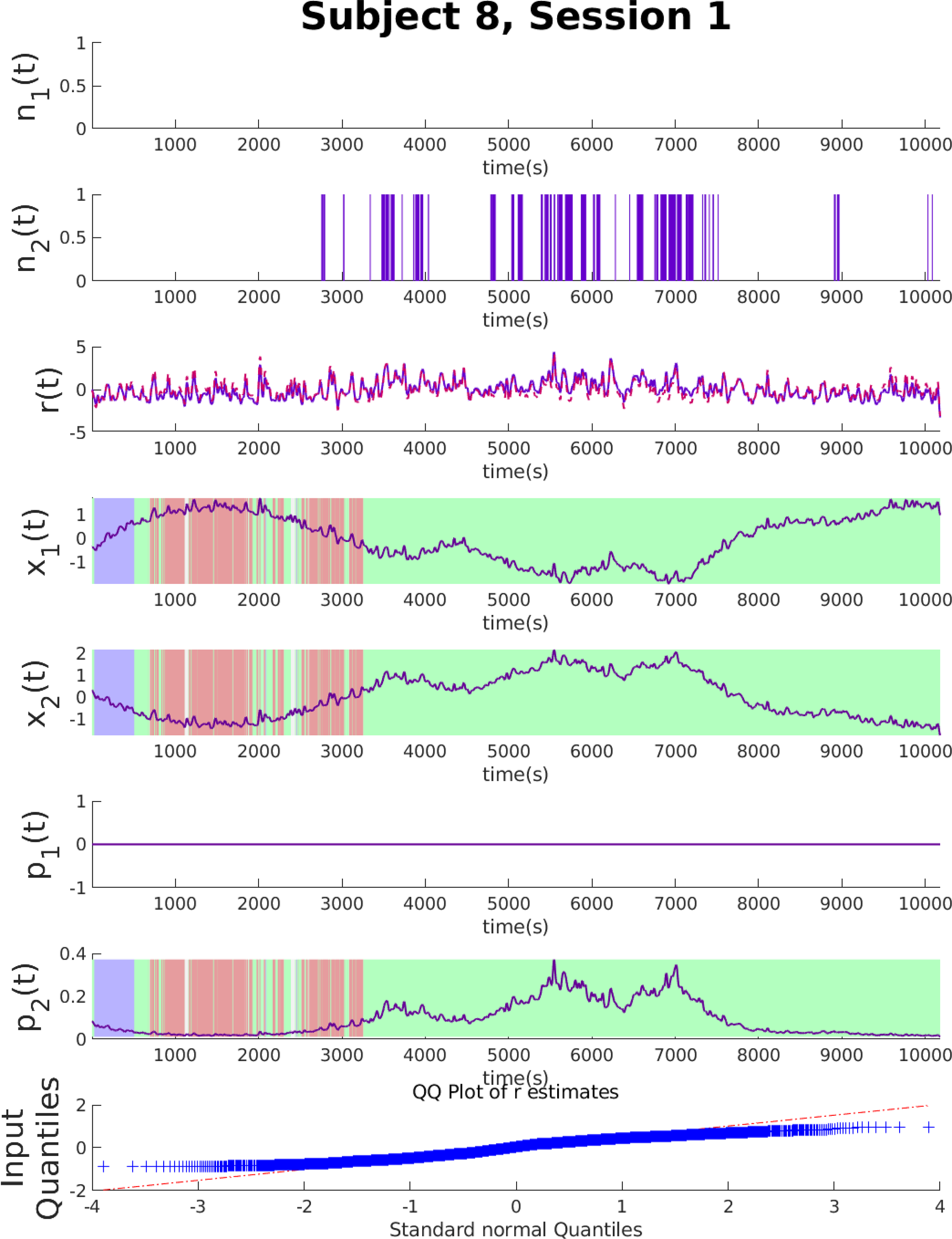

Supplement: S22 Fig — The panel shows the experimental data for no stressor sessions. From top, the binary variables n1 and n2 derived from deconvolved EDA data and typing data respectively, the continuous variable r denoting the RR intervals derived from heart rate (red line) and r˜ estimated from latent variables x1 and x2 (purple line), x1 and x2 in order from top indicating cognitive arousal state and expressive typing state respectively. p1 and p2 show the estimated probabilities. Patches of green, red, and cyan indicate what application the subject was using at the time of measurement. Green indicates applications for information search like internet explorer, red is for typing like Microsoft word and PowerPoint and cyan is for when subjects are looking at their emails. Finally, the QQ plot for the residual error of r is shown. (TIF) [file pone.0300786.s023.tif]

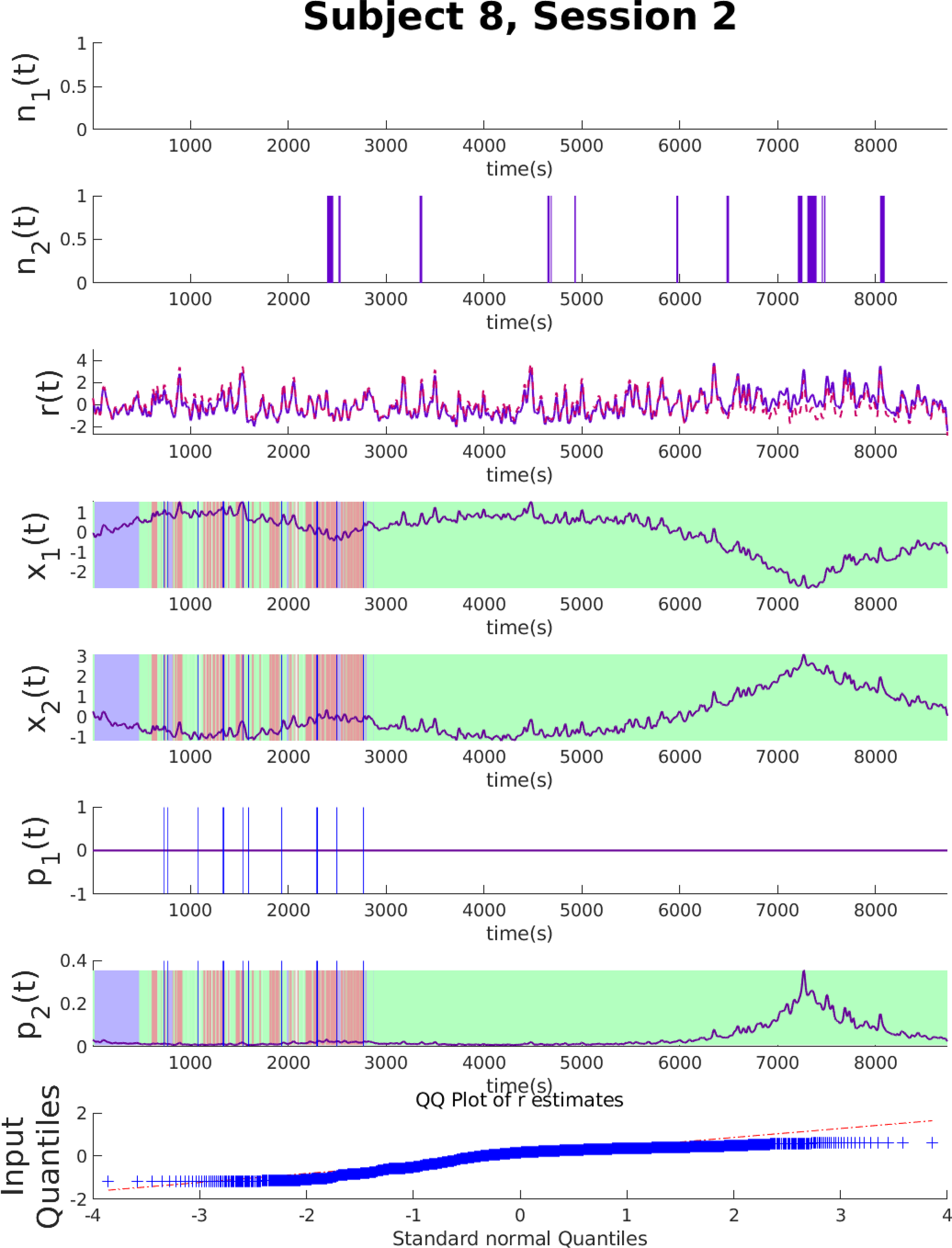

Supplement: S23 Fig — The panel shows the experimental data with time limit. From top, the binary variables n1 and n2 derived from deconvolved EDA data and typing data respectively, the continuous variable r denoting the RR intervals derived from heart rate (red line) and r˜ estimated from latent variables x1 and x2 (purple line), x1 and x2 in order from top indicating cognitive arousal state and expressive typing state respectively. p1 and p2 show the estimated probabilities. Patches of green, red, and cyan indicate what application the subject was using at the time of measurement. Green indicates applications for information search like internet explorer, red is for typing like Microsoft word and PowerPoint and cyan is for when subjects are looking at their emails. Finally, the QQ plot for the residual error of r is shown. (TIF) [file pone.0300786.s024.tif]

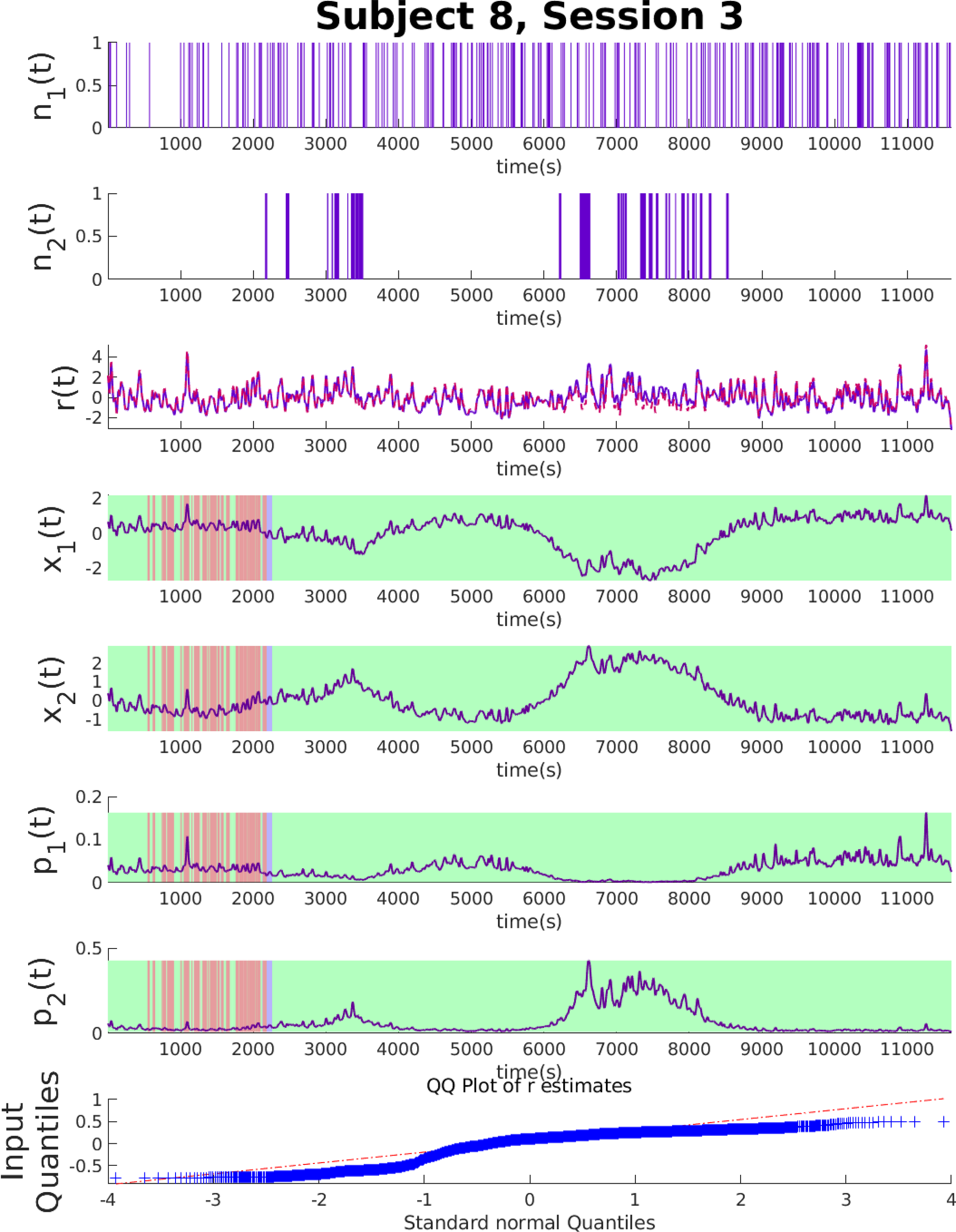

Supplement: S24 Fig — The panel shows the experimental data with interruptions. From top, the binary variables n1 and n2 derived from deconvolved EDA data and typing data respectively, the continuous variable r denoting the RR intervals derived from heart rate (red line) and r˜ estimated from latent variables x1 and x2 (purple line), x1 and x2 in order from top indicating cognitive arousal state and expressive typing state respectively. p1 and p2 show the estimated probabilities. Patches of green, red, and cyan indicate what application the subject was using at the time of measurement. Green indicates applications for information search like internet explorer, red is for typing like Microsoft word and PowerPoint and cyan is for when subjects are looking at their emails. The Blue vertical line indicates the time email notifications were sent. Finally, the QQ plot for the residual error of r is shown. (TIF) [file pone.0300786.s025.tif]

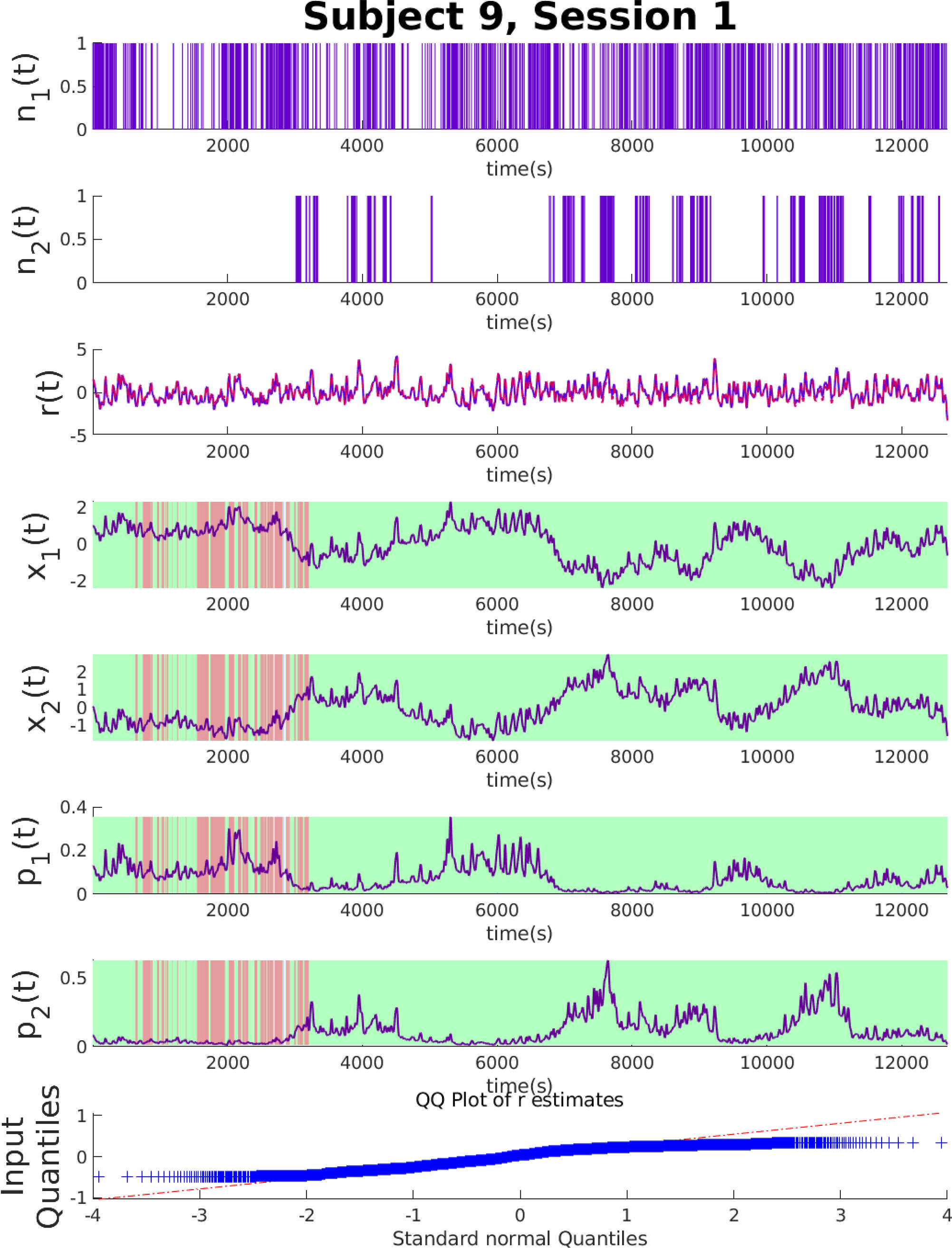

Supplement: S25 Fig — The panel shows the experimental data for no stressor sessions. From top, the binary variables n1 and n2 derived from deconvolved EDA data and typing data respectively, the continuous variable r denoting the RR intervals derived from heart rate (red line) and r˜ estimated from latent variables x1 and x2 (purple line), x1 and x2 in order from top indicating cognitive arousal state and expressive typing state respectively. p1 and p2 show the estimated probabilities. Patches of green, red, and cyan indicate what application the subject was using at the time of measurement. Green indicates applications for information search like internet explorer, red is for typing like Microsoft word and PowerPoint and cyan is for when subjects are looking at their emails. Finally, the QQ plot for the residual error of r is shown. (TIF) [file pone.0300786.s026.tif]

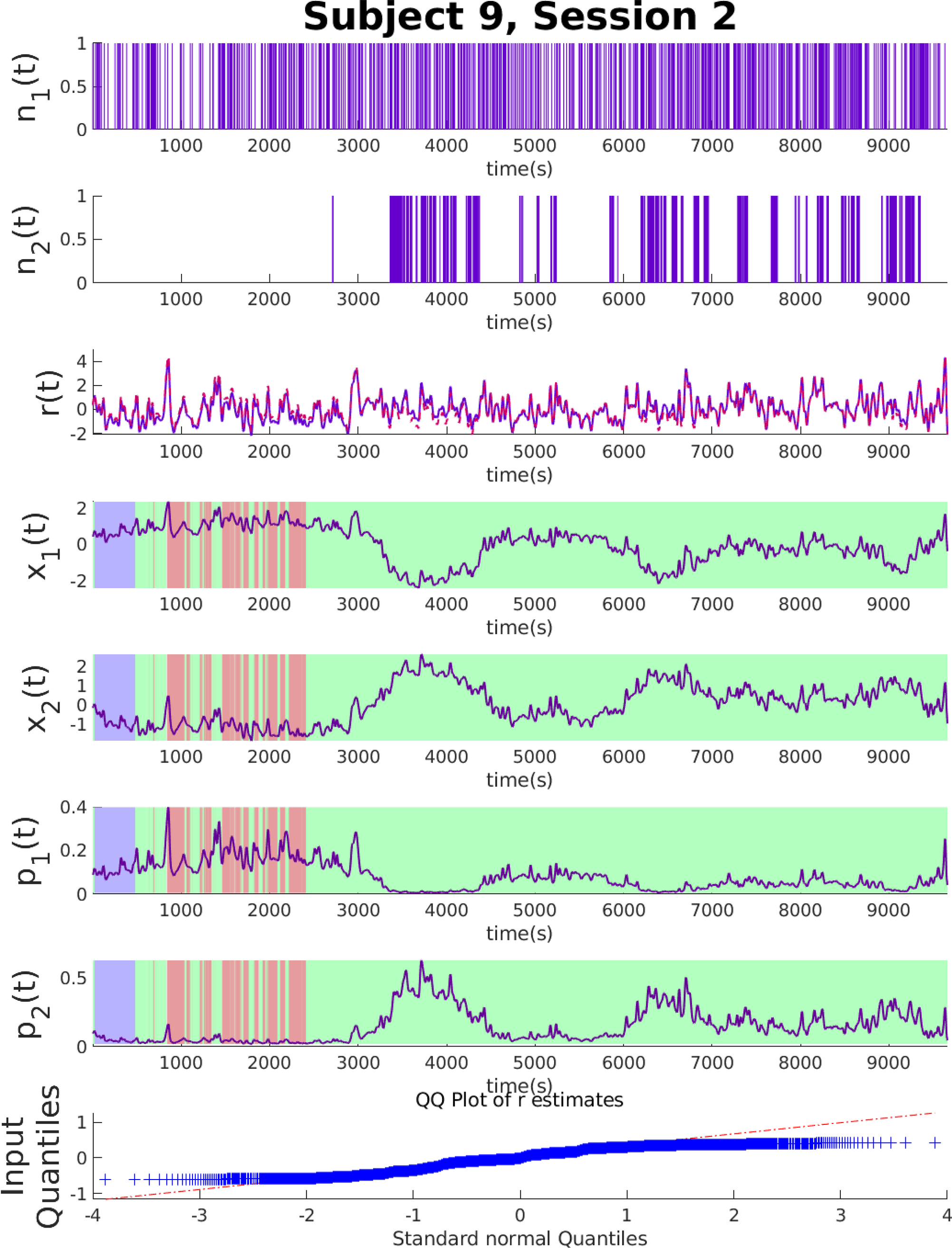

Supplement: S26 Fig — The panel shows the experimental data with time limit. From top, the binary variables n1 and n2 derived from deconvolved EDA data and typing data respectively, the continuous variable r denoting the RR intervals derived from heart rate (red line) and r˜ estimated from latent variables x1 and x2 (purple line), x1 and x2 in order from top indicating cognitive arousal state and expressive typing state respectively. p1 and p2 show the estimated probabilities. Patches of green, red, and cyan indicate what application the subject was using at the time of measurement. Green indicates applications for information search like internet explorer, red is for typing like Microsoft word and PowerPoint and cyan is for when subjects are looking at their emails. Finally, the QQ plot for the residual error of r is shown. (TIF) [file pone.0300786.s027.tif]

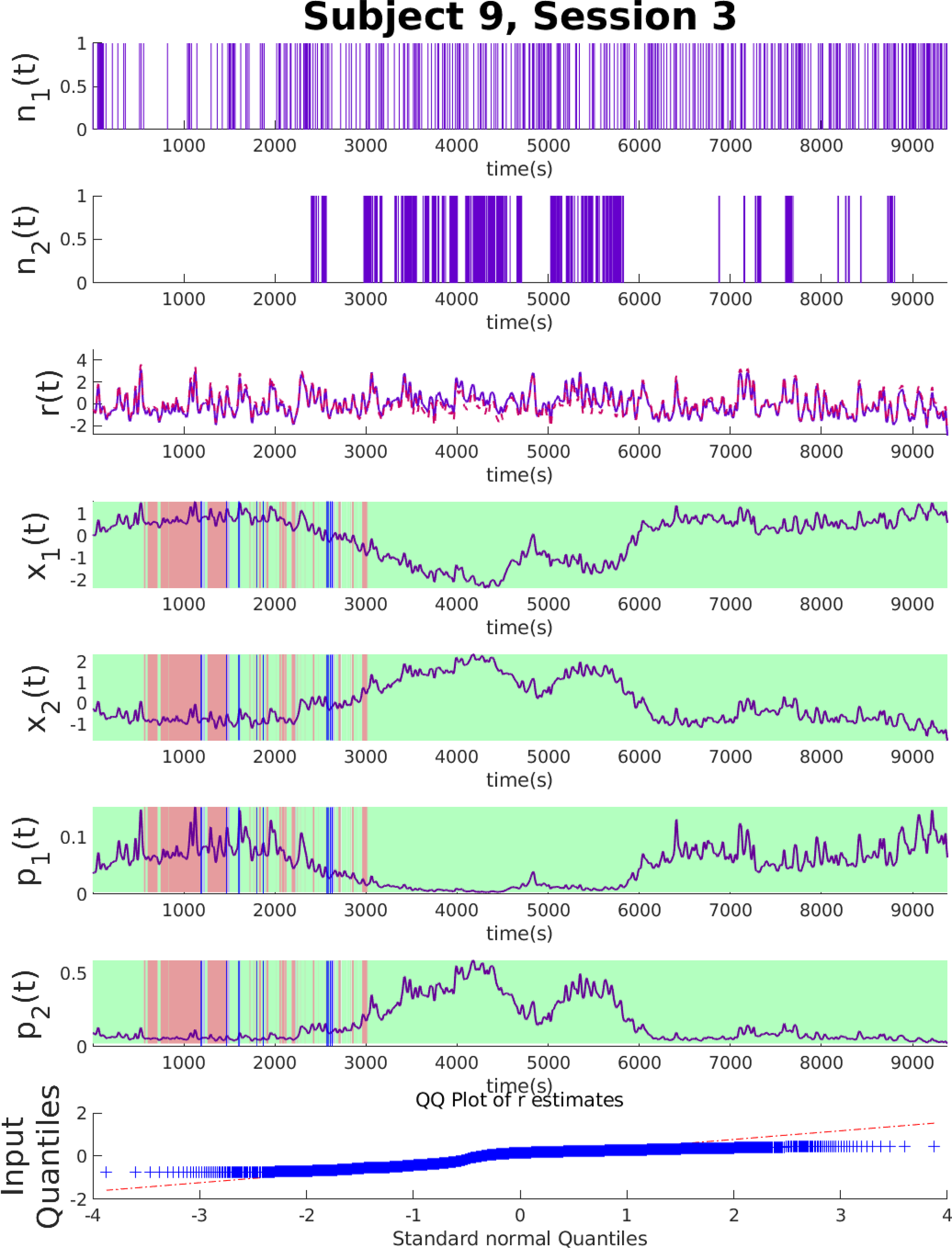

Supplement: S27 Fig — The panel shows the experimental data with interruptions. From top, the binary variables n1 and n2 derived from deconvolved EDA data and typing data respectively, the continuous variable r denoting the RR intervals derived from heart rate (red line) and r˜ estimated from latent variables x1 and x2 (purple line), x1 and x2 in order from top indicating cognitive arousal state and expressive typing state respectively. p1 and p2 show the estimated probabilities. Patches of green, red, and cyan indicate what application the subject was using at the time of measurement. Green indicates applications for information search like internet explorer, red is for typing like Microsoft word and PowerPoint and cyan is for when subjects are looking at their emails. The Blue vertical line indicates the time email notifications were sent. Finally, the QQ plot for the residual error of r is shown. (TIF) [file pone.0300786.s028.tif]

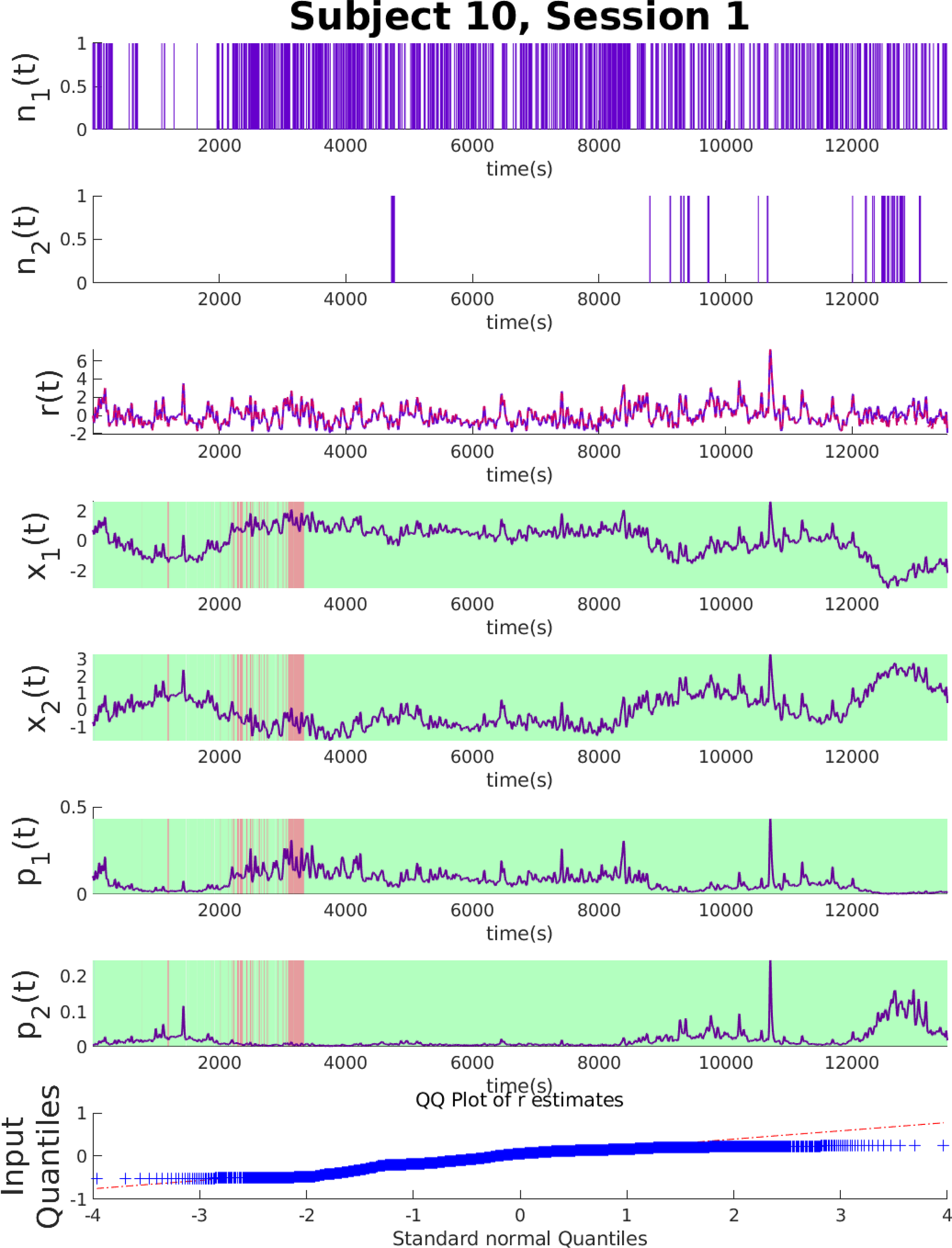

Supplement: S28 Fig — The panel shows the experimental data for no stressor sessions. From top, the binary variables n1 and n2 derived from deconvolved EDA data and typing data respectively, the continuous variable r denoting the RR intervals derived from heart rate (red line) and r˜ estimated from latent variables x1 and x2 (purple line), x1 and x2 in order from top indicating cognitive arousal state and expressive typing state respectively. p1 and p2 show the estimated probabilities. Patches of green, red, and cyan indicate what application the subject was using at the time of measurement. Green indicates applications for information search like internet explorer, red is for typing like Microsoft word and PowerPoint and cyan is for when subjects are looking at their emails. Finally, the QQ plot for the residual error of r is shown. (TIF) [file pone.0300786.s029.tif]

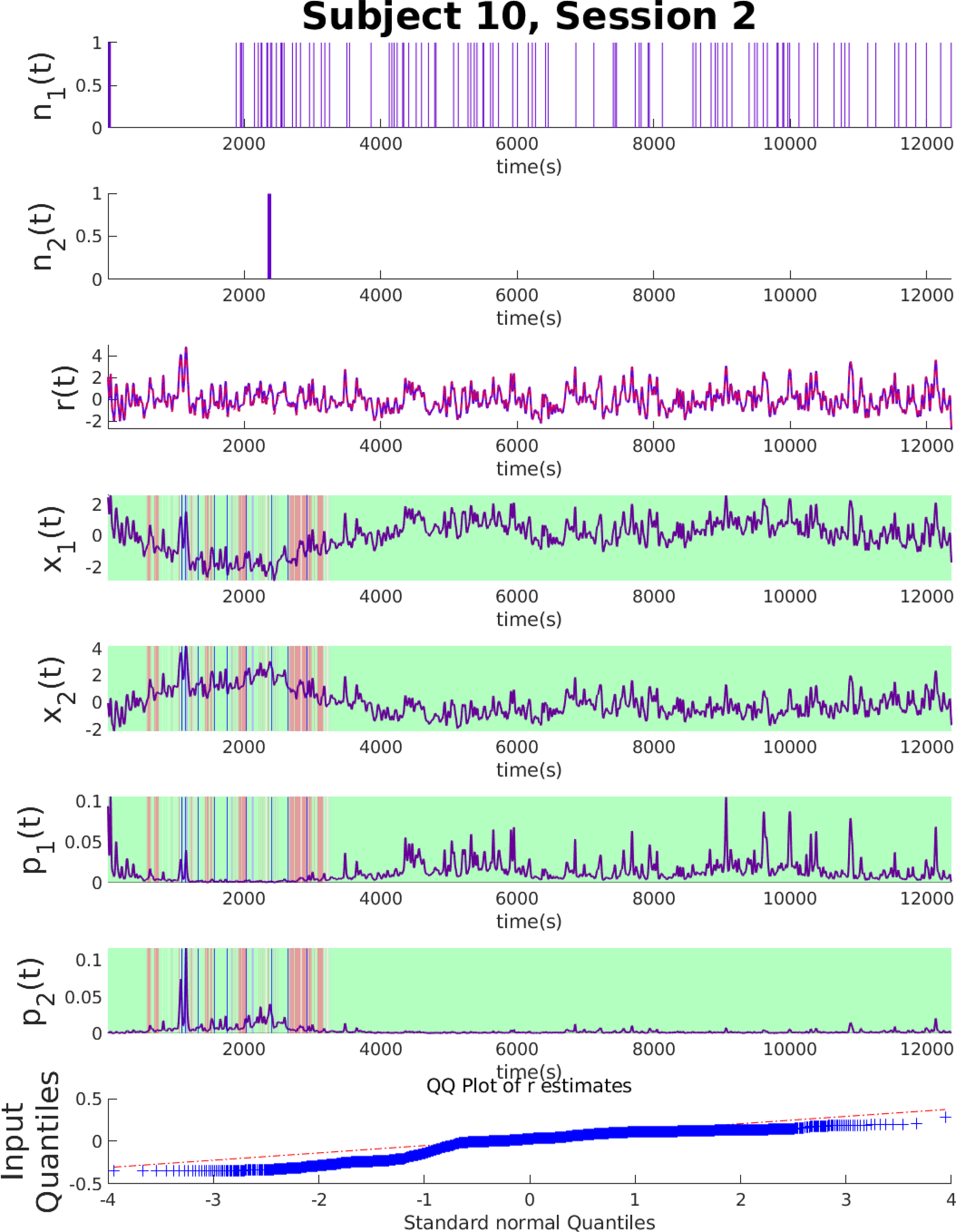

Supplement: S29 Fig — The panel shows the experimental data with time limit. From top, the binary variables n1 and n2 derived from deconvolved EDA data and typing data respectively, the continuous variable r denoting the RR intervals derived from heart rate (red line) and r˜ estimated from latent variables x1 and x2 (purple line), x1 and x2 in order from top indicating cognitive arousal state and expressive typing state respectively. p1 and p2 show the estimated probabilities. Patches of green, red, and cyan indicate what application the subject was using at the time of measurement. Green indicates applications for information search like internet explorer, red is for typing like Microsoft word and PowerPoint and cyan is for when subjects are looking at their emails. Finally, the QQ plot for the residual error of r is shown. (TIF) [file pone.0300786.s030.tif]

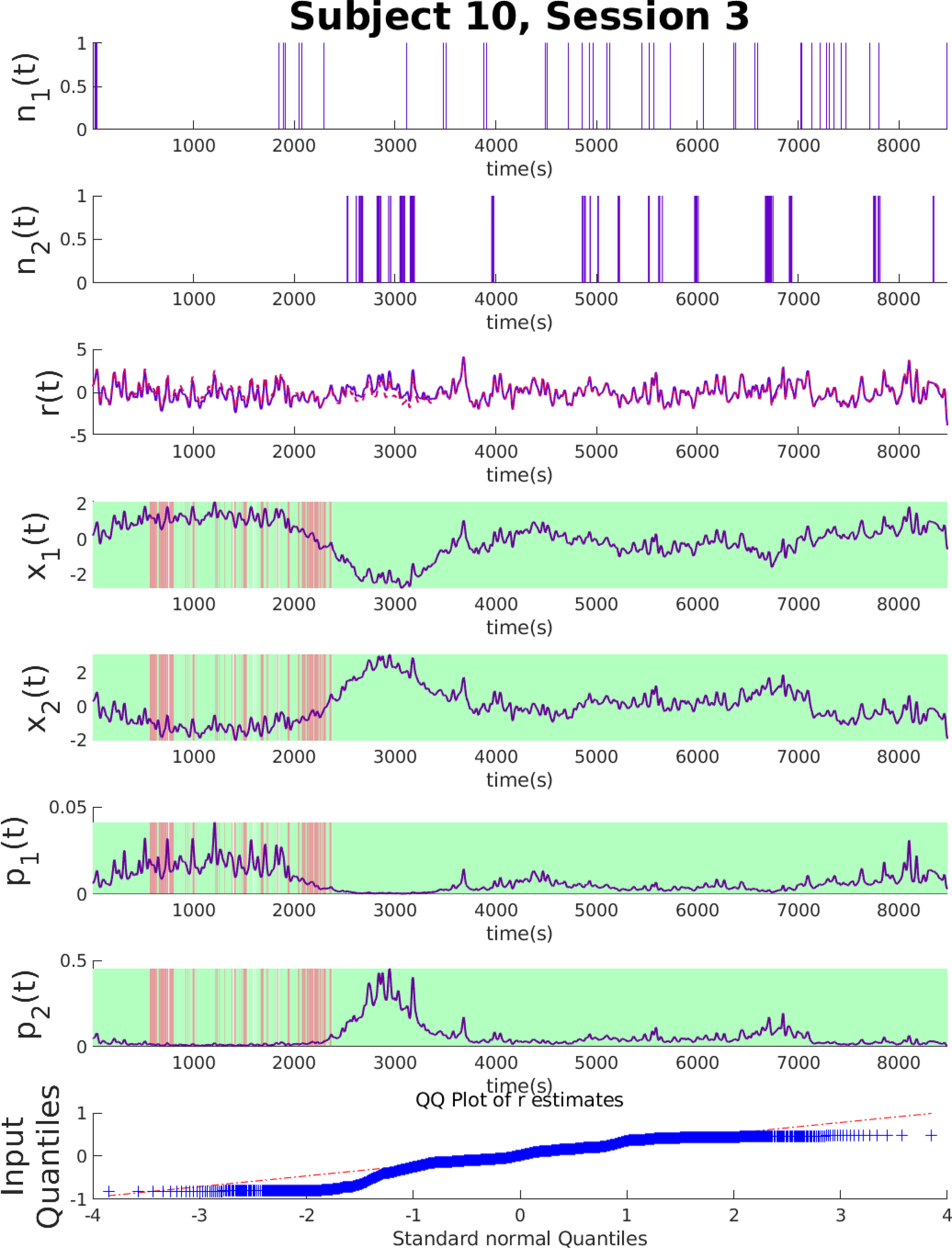

Supplement: S30 Fig — The panel shows the experimental data with interruptions. From top, the binary variables n1 and n2 derived from deconvolved EDA data and typing data respectively, the continuous variable r denoting the RR intervals derived from heart rate (red line) and r˜ estimated from latent variables x1 and x2 (purple line), x1 and x2 in order from top indicating cognitive arousal state and expressive typing state respectively. p1 and p2 show the estimated probabilities. Patches of green, red, and cyan indicate what application the subject was using at the time of measurement. Green indicates applications for information search like internet explorer, red is for typing like Microsoft word and PowerPoint and cyan is for when subjects are looking at their emails. The Blue vertical line indicates the time email notifications were sent. Finally, the QQ plot for the residual error of r is shown. (TIF) [file pone.0300786.s031.tif]

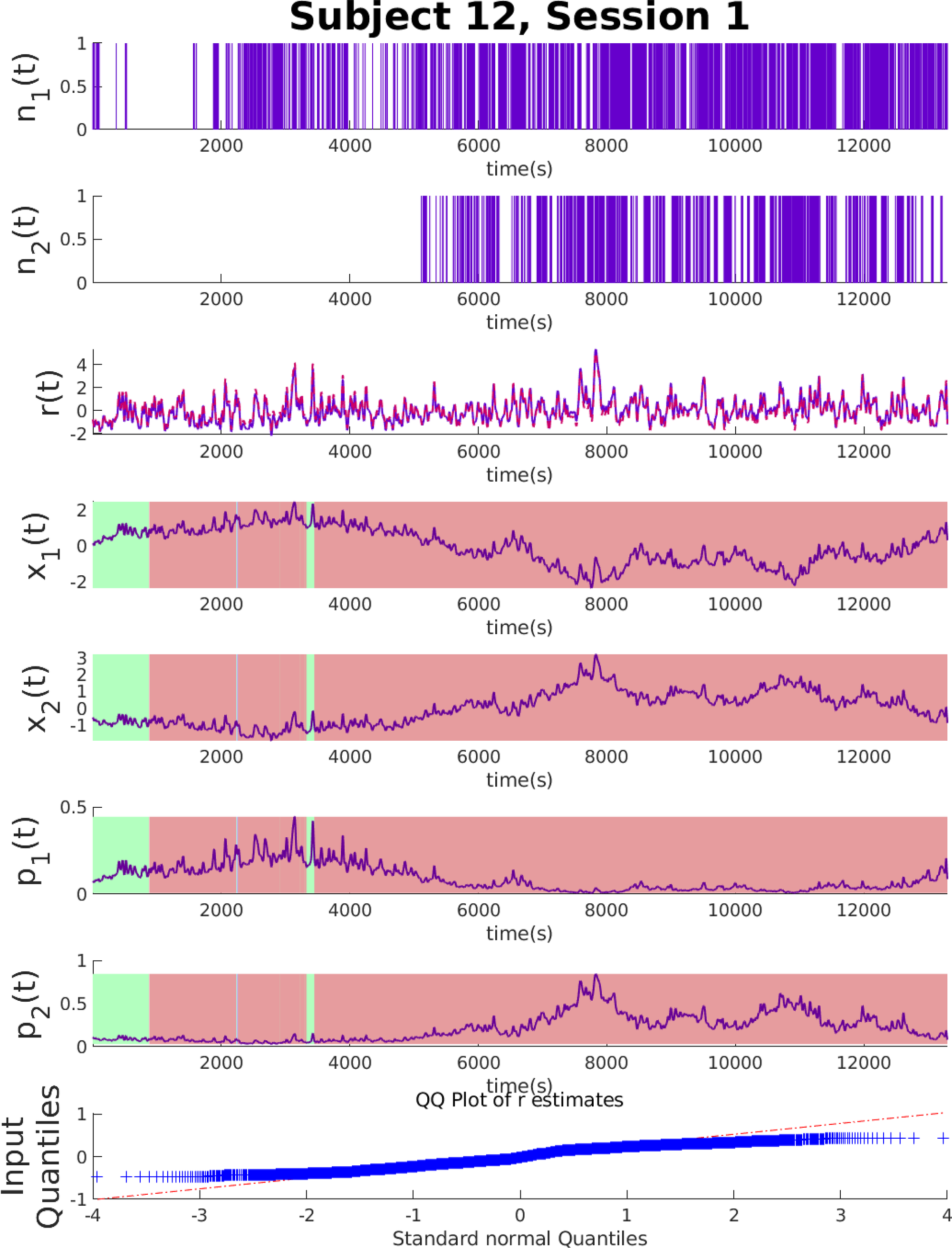

Supplement: S31 Fig — The panel shows the experimental data for no stressor sessions. From top, the binary variables n1 and n2 derived from deconvolved EDA data and typing data respectively, the continuous variable r denoting the RR intervals derived from heart rate (red line) and r˜ estimated from latent variables x1 and x2 (purple line), x1 and x2 in order from top indicating cognitive arousal state and expressive typing state respectively. p1 and p2 show the estimated probabilities. Patches of green, red, and cyan indicate what application the subject was using at the time of measurement. Green indicates applications for information search like internet explorer, red is for typing like Microsoft word and PowerPoint and cyan is for when subjects are looking at their emails. Finally, the QQ plot for the residual error of r is shown. (TIF) [file pone.0300786.s032.tif]

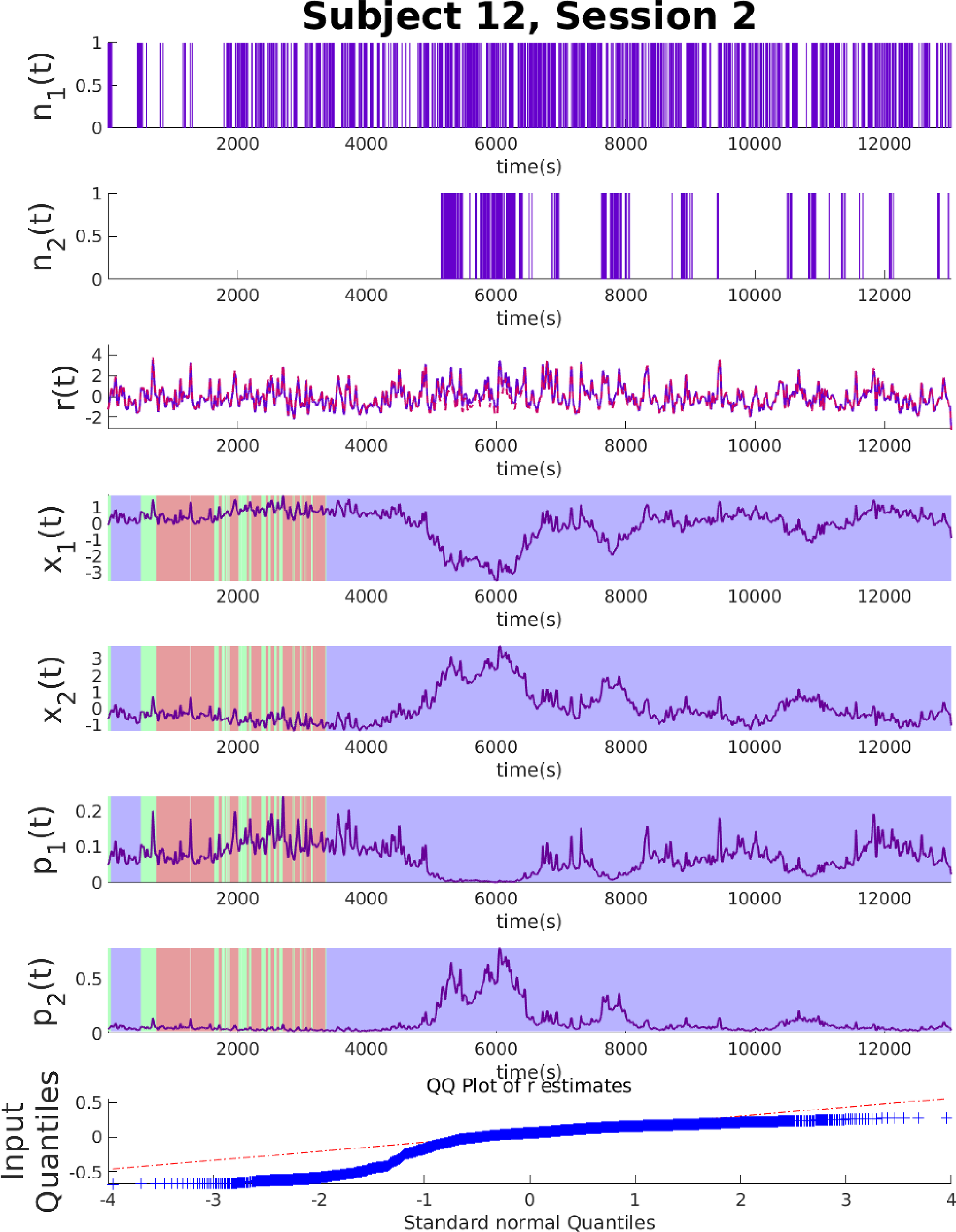

Supplement: S32 Fig — The panel shows the experimental data with time limit. From top, the binary variables n1 and n2 derived from deconvolved EDA data and typing data respectively, the continuous variable r denoting the RR intervals derived from heart rate (red line) and r˜ estimated from latent variables x1 and x2 (purple line), x1 and x2 in order from top indicating cognitive arousal state and expressive typing state respectively. p1 and p2 show the estimated probabilities. Patches of green, red, and cyan indicate what application the subject was using at the time of measurement. Green indicates applications for information search like internet explorer, red is for typing like Microsoft word and PowerPoint and cyan is for when subjects are looking at their emails. Finally, the QQ plot for the residual error of r is shown. (TIF) [file pone.0300786.s033.tif]

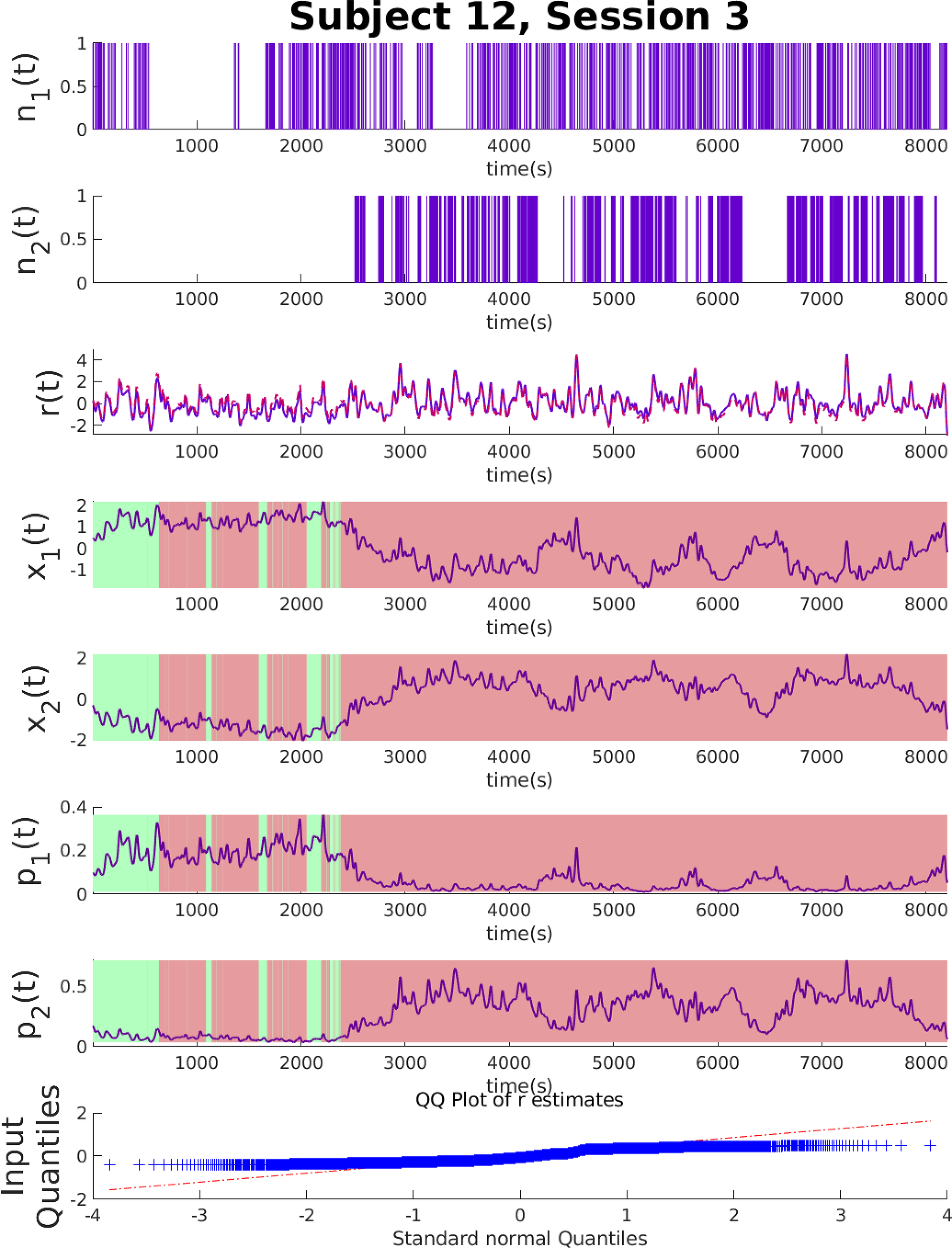

Supplement: S33 Fig — The panel shows the experimental data with interruptions. From top, the binary variables n1 and n2 derived from deconvolved EDA data and typing data respectively, the continuous variable r denoting the RR intervals derived from heart rate (red line) and r˜ estimated from latent variables x1 and x2 (purple line), x1 and x2 in order from top indicating cognitive arousal state and expressive typing state respectively. p1 and p2 show the estimated probabilities. Patches of green, red, and cyan indicate what application the subject was using at the time of measurement. Green indicates applications for information search like internet explorer, red is for typing like Microsoft word and PowerPoint and cyan is for when subjects are looking at their emails. The Blue vertical line indicates the time email notifications were sent. Finally, the QQ plot for the residual error of r is shown. (TIF) [file pone.0300786.s034.tif]

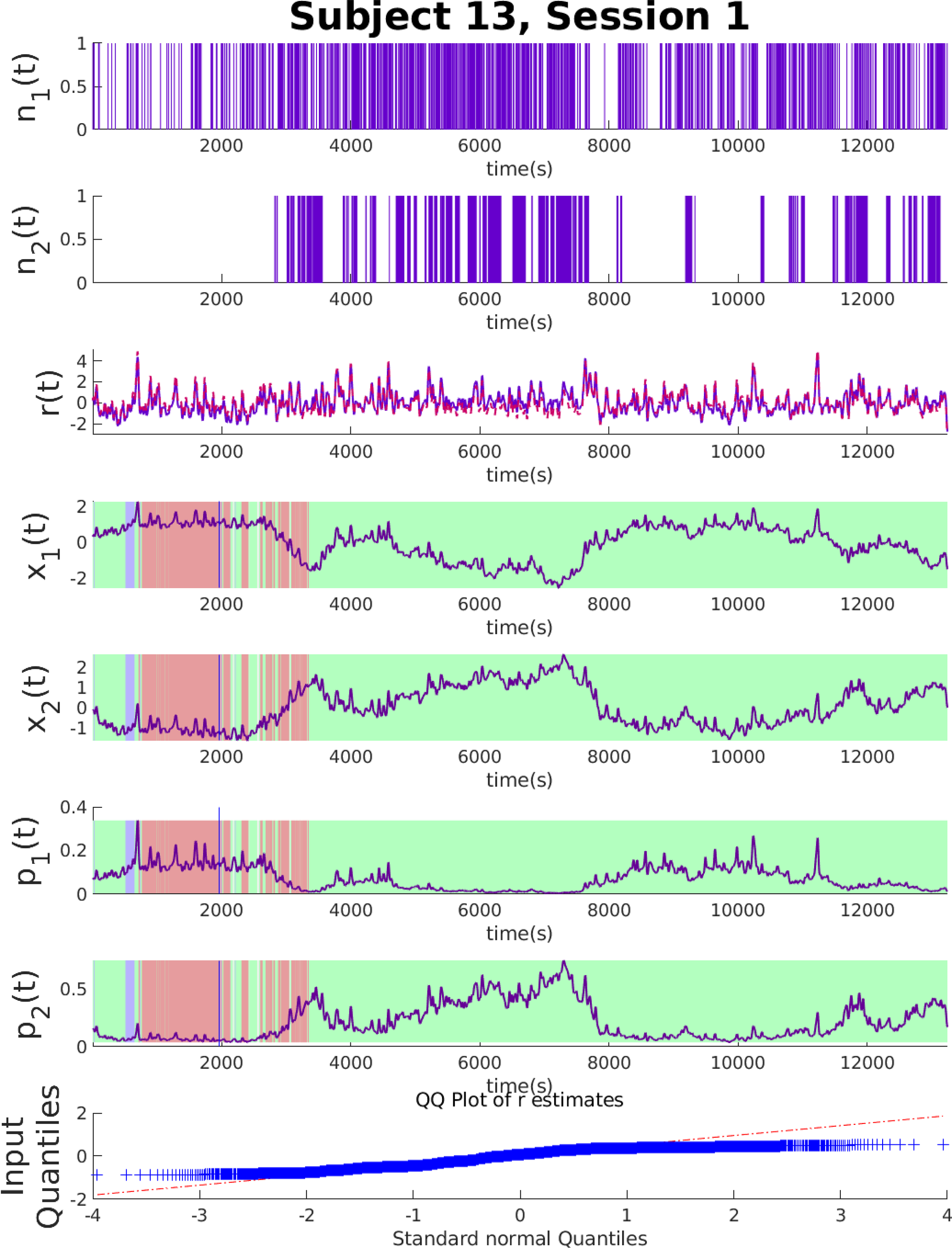

Supplement: S34 Fig — The panel shows the experimental data for no stressor sessions. From top, the binary variables n1 and n2 derived from deconvolved EDA data and typing data respectively, the continuous variable r denoting the RR intervals derived from heart rate (red line) and r˜ estimated from latent variables x1 and x2 (purple line), x1 and x2 in order from top indicating cognitive arousal state and expressive typing state respectively. p1 and p2 show the estimated probabilities. Patches of green, red, and cyan indicate what application the subject was using at the time of measurement. Green indicates applications for information search like internet explorer, red is for typing like Microsoft word and PowerPoint and cyan is for when subjects are looking at their emails. Finally, the QQ plot for the residual error of r is shown. (TIF) [file pone.0300786.s035.tif]

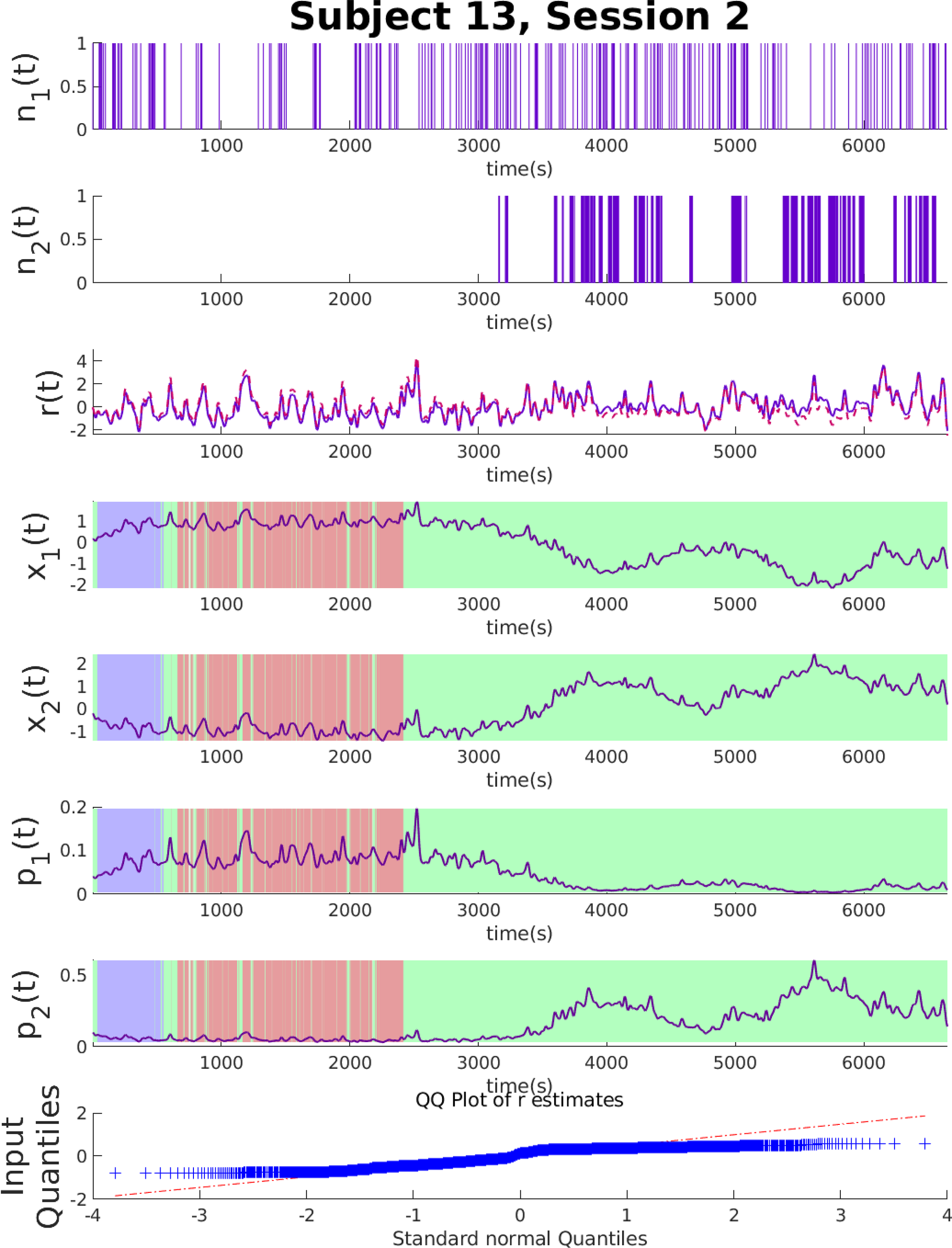

Supplement: S35 Fig — The panel shows the experimental data with time limit. From top, the binary variables n1 and n2 derived from deconvolved EDA data and typing data respectively, the continuous variable r denoting the RR intervals derived from heart rate (red line) and r˜ estimated from latent variables x1 and x2 (purple line), x1 and x2 in order from top indicating cognitive arousal state and expressive typing state respectively. p1 and p2 show the estimated probabilities. Patches of green, red, and cyan indicate what application the subject was using at the time of measurement. Green indicates applications for information search like internet explorer, red is for typing like Microsoft word and PowerPoint and cyan is for when subjects are looking at their emails. Finally, the QQ plot for the residual error of r is shown. (TIF) [file pone.0300786.s036.tif]

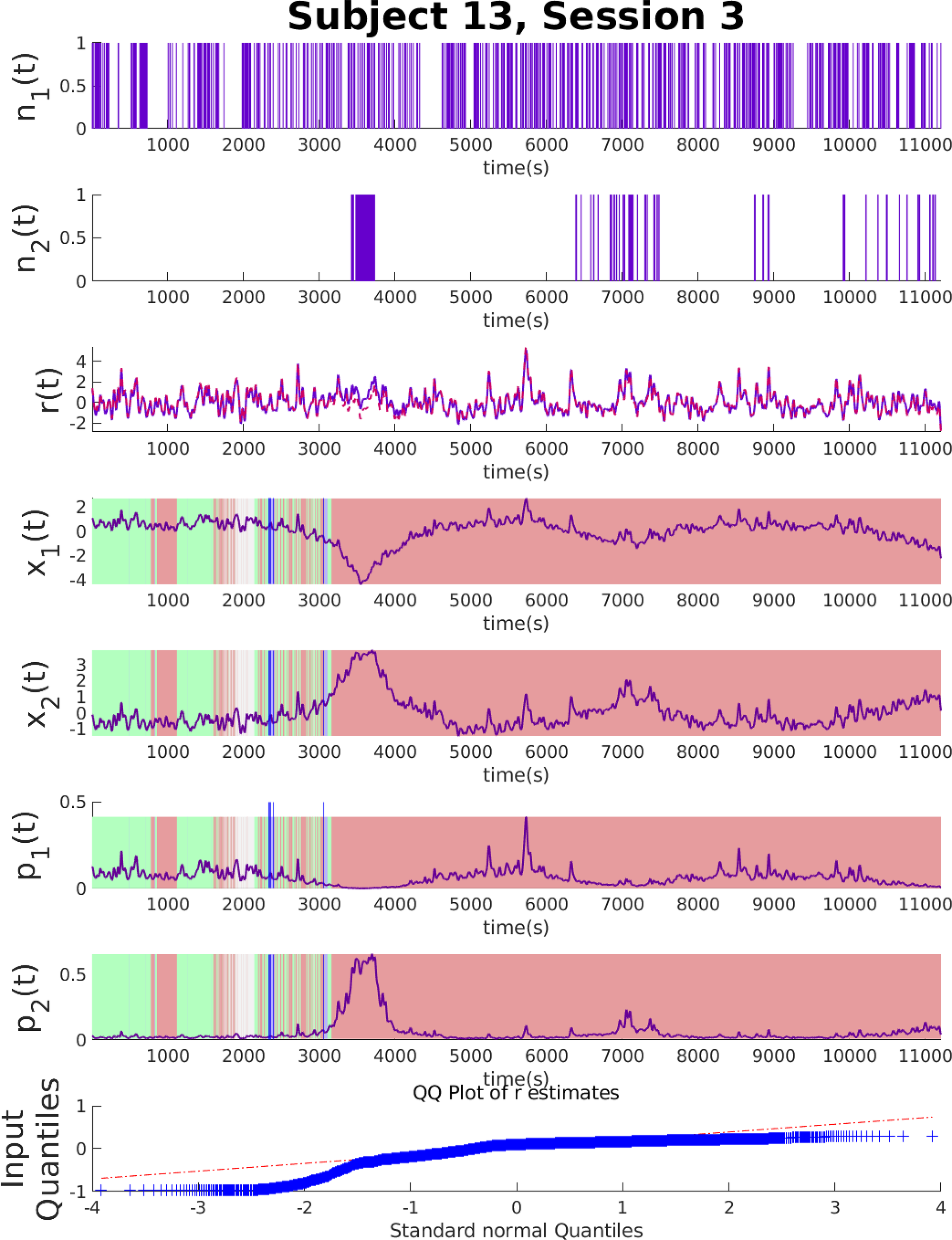

Supplement: S36 Fig — The panel shows the experimental data with interruptions. From top, the binary variables n1 and n2 derived from deconvolved EDA data and typing data respectively, the continuous variable r denoting the RR intervals derived from heart rate (red line) and r˜ estimated from latent variables x1 and x2 (purple line), x1 and x2 in order from top indicating cognitive arousal state and expressive typing state respectively. p1 and p2 show the estimated probabilities. Patches of green, red, and cyan indicate what application the subject was using at the time of measurement. Green indicates applications for information search like internet explorer, red is for typing like Microsoft word and PowerPoint and cyan is for when subjects are looking at their emails. The Blue vertical line indicates the time email notifications were sent. Finally, the QQ plot for the residual error of r is shown. (TIF) [file pone.0300786.s037.tif]

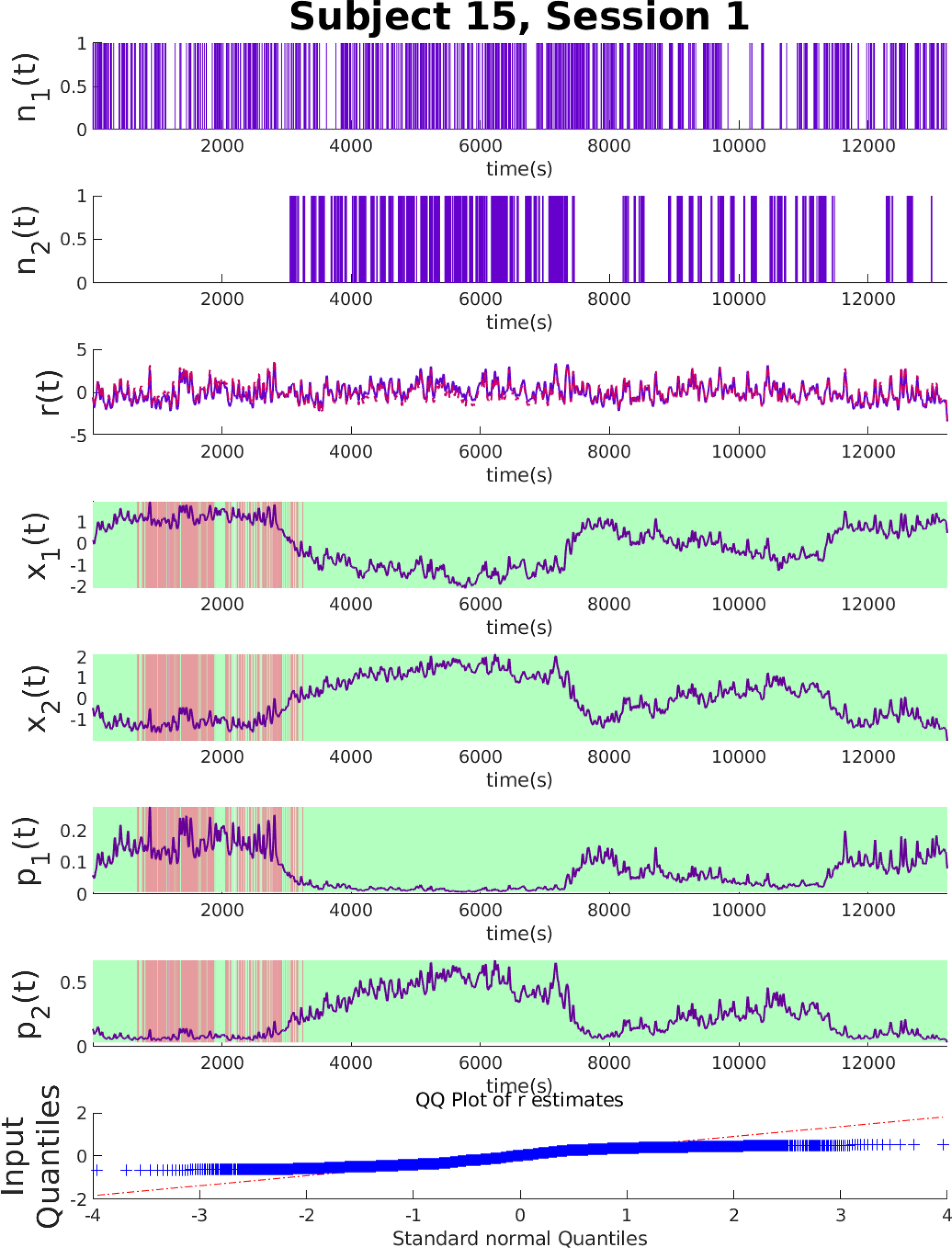

Supplement: S37 Fig — The panel shows the experimental data for no stressor sessions. From top, the binary variables n1 and n2 derived from deconvolved EDA data and typing data respectively, the continuous variable r denoting the RR intervals derived from heart rate (red line) and r˜ estimated from latent variables x1 and x2 (purple line), x1 and x2 in order from top indicating cognitive arousal state and expressive typing state respectively. p1 and p2 show the estimated probabilities. Patches of green, red, and cyan indicate what application the subject was using at the time of measurement. Green indicates applications for information search like internet explorer, red is for typing like Microsoft word and PowerPoint and cyan is for when subjects are looking at their emails. Finally, the QQ plot for the residual error of r is shown. (TIF) [file pone.0300786.s038.tif]

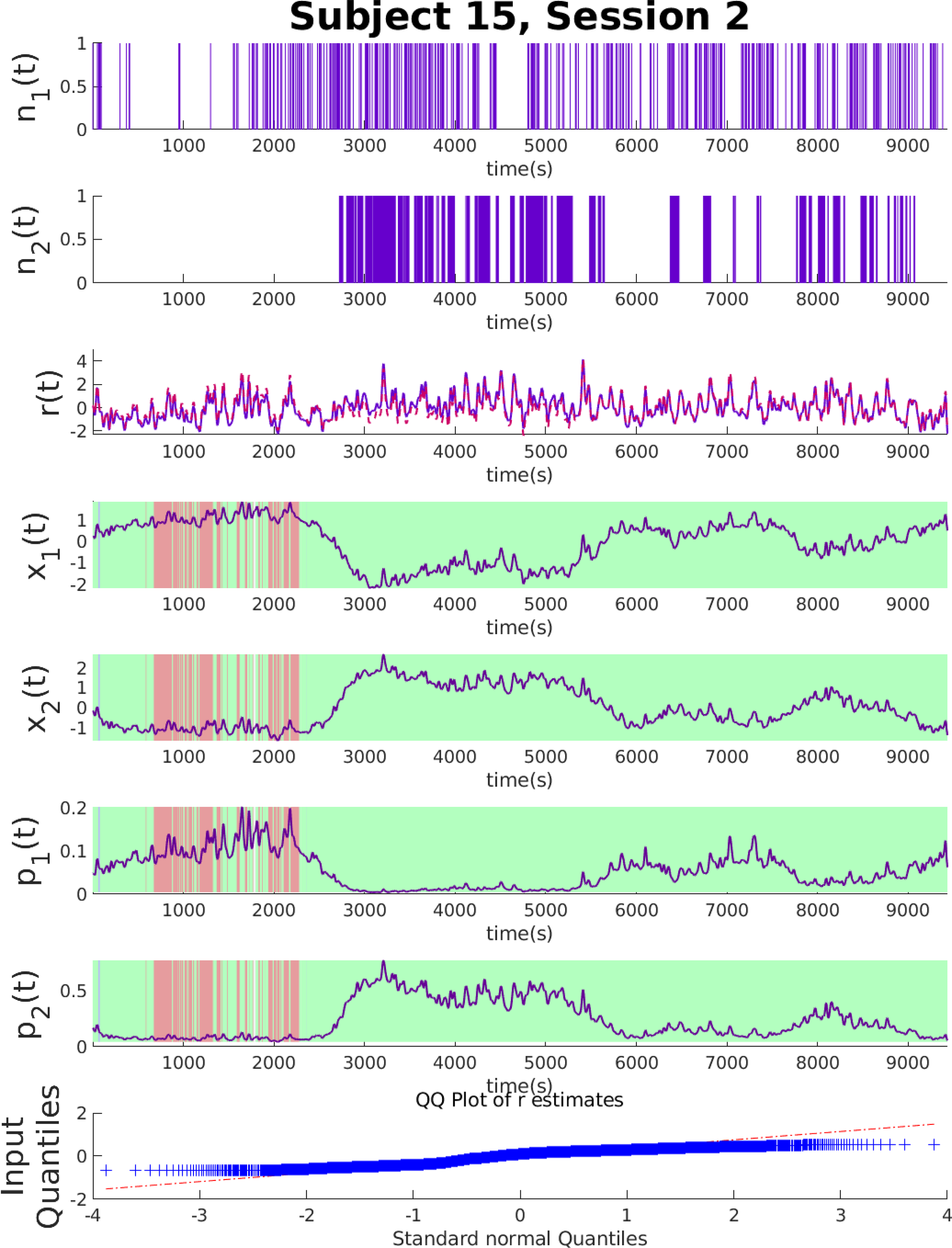

Supplement: S38 Fig — The panel shows the experimental data with time limit. From top, the binary variables n1 and n2 derived from deconvolved EDA data and typing data respectively, the continuous variable r denoting the RR intervals derived from heart rate (red line) and r˜ estimated from latent variables x1 and x2 (purple line), x1 and x2 in order from top indicating cognitive arousal state and expressive typing state respectively. p1 and p2 show the estimated probabilities. Patches of green, red, and cyan indicate what application the subject was using at the time of measurement. Green indicates applications for information search like internet explorer, red is for typing like Microsoft word and PowerPoint and cyan is for when subjects are looking at their emails. Finally, the QQ plot for the residual error of r is shown. (TIF) [file pone.0300786.s039.tif]

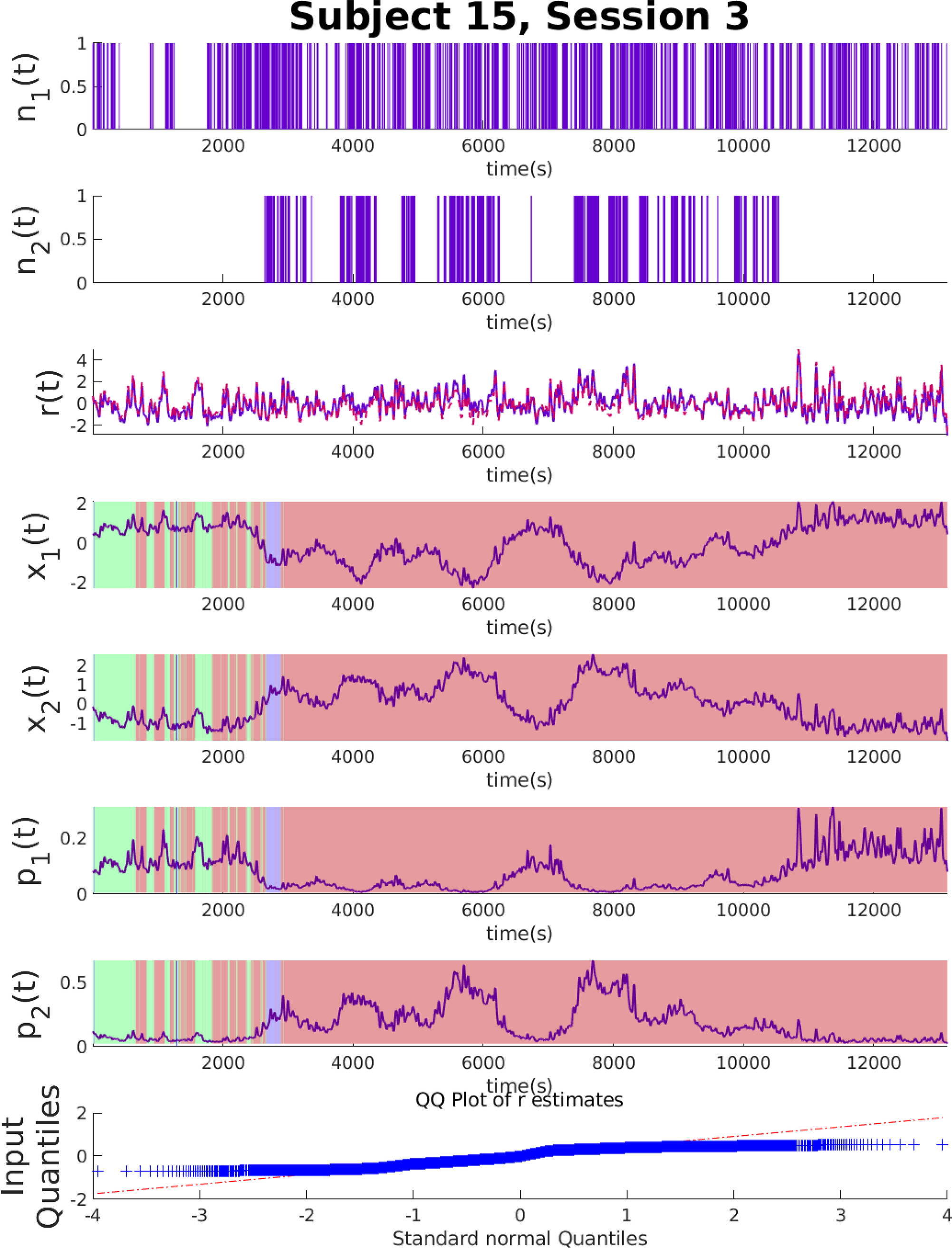

Supplement: S39 Fig — The panel shows the experimental data with interruptions. From top, the binary variables n1 and n2 derived from deconvolved EDA data and typing data respectively, the continuous variable r denoting the RR intervals derived from heart rate (red line) and r˜ estimated from latent variables x1 and x2 (purple line), x1 and x2 in order from top indicating cognitive arousal state and expressive typing state respectively. p1 and p2 show the estimated probabilities. Patches of green, red, and cyan indicate what application the subject was using at the time of measurement. Green indicates applications for information search like internet explorer, red is for typing like Microsoft word and PowerPoint and cyan is for when subjects are looking at their emails. The Blue vertical line indicates the time email notifications were sent. Finally, the QQ plot for the residual error of r is shown. (TIF) [file pone.0300786.s040.tif]

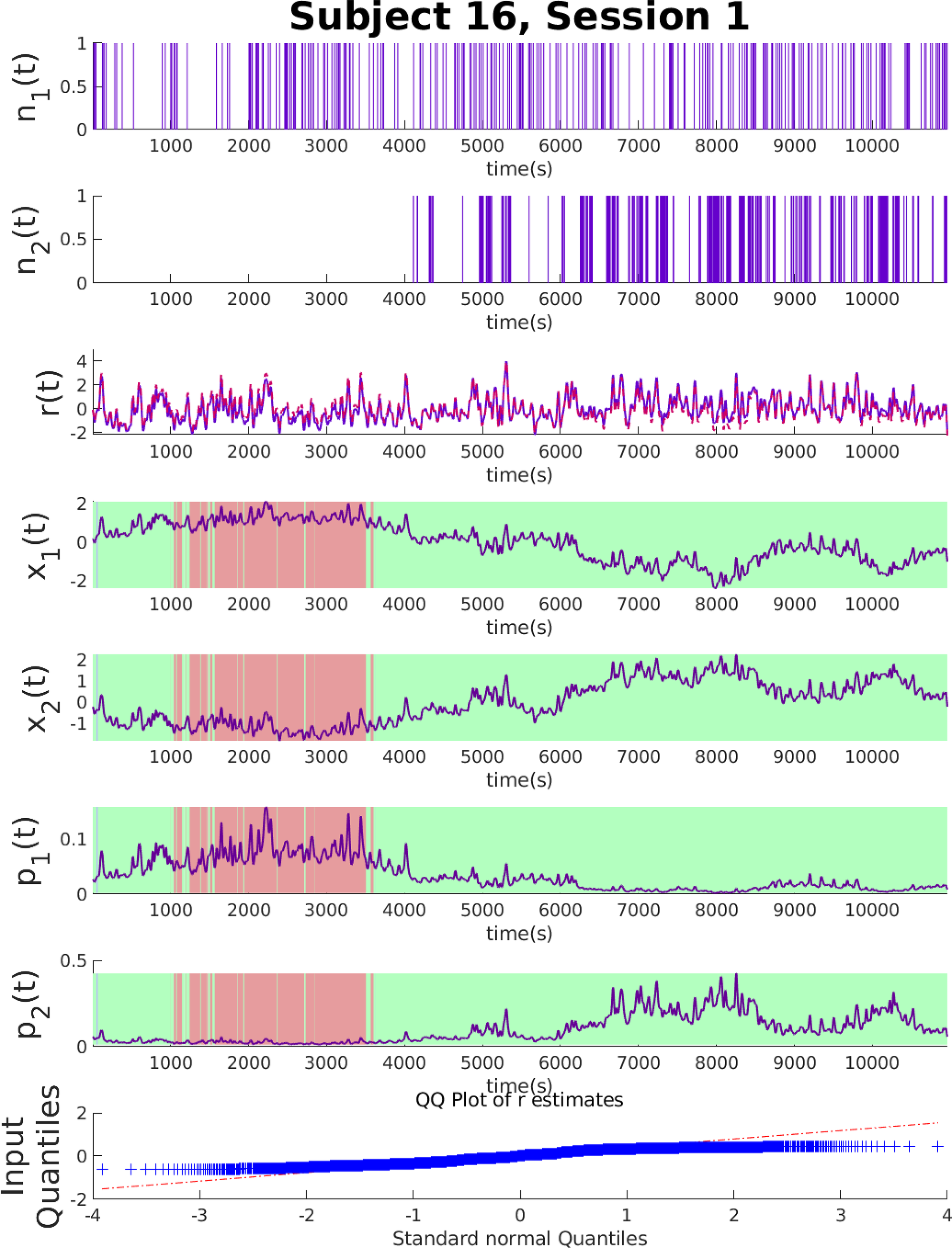

Supplement: S40 Fig — The panel shows the experimental data for no stressor sessions. From top, the binary variables n1 and n2 derived from deconvolved EDA data and typing data respectively, the continuous variable r denoting the RR intervals derived from heart rate (red line) and r˜ estimated from latent variables x1 and x2 (purple line), x1 and x2 in order from top indicating cognitive arousal state and expressive typing state respectively. p1 and p2 show the estimated probabilities. Patches of green, red, and cyan indicate what application the subject was using at the time of measurement. Green indicates applications for information search like internet explorer, red is for typing like Microsoft word and PowerPoint and cyan is for when subjects are looking at their emails. Finally, the QQ plot for the residual error of r is shown. (TIF) [file pone.0300786.s041.tif]

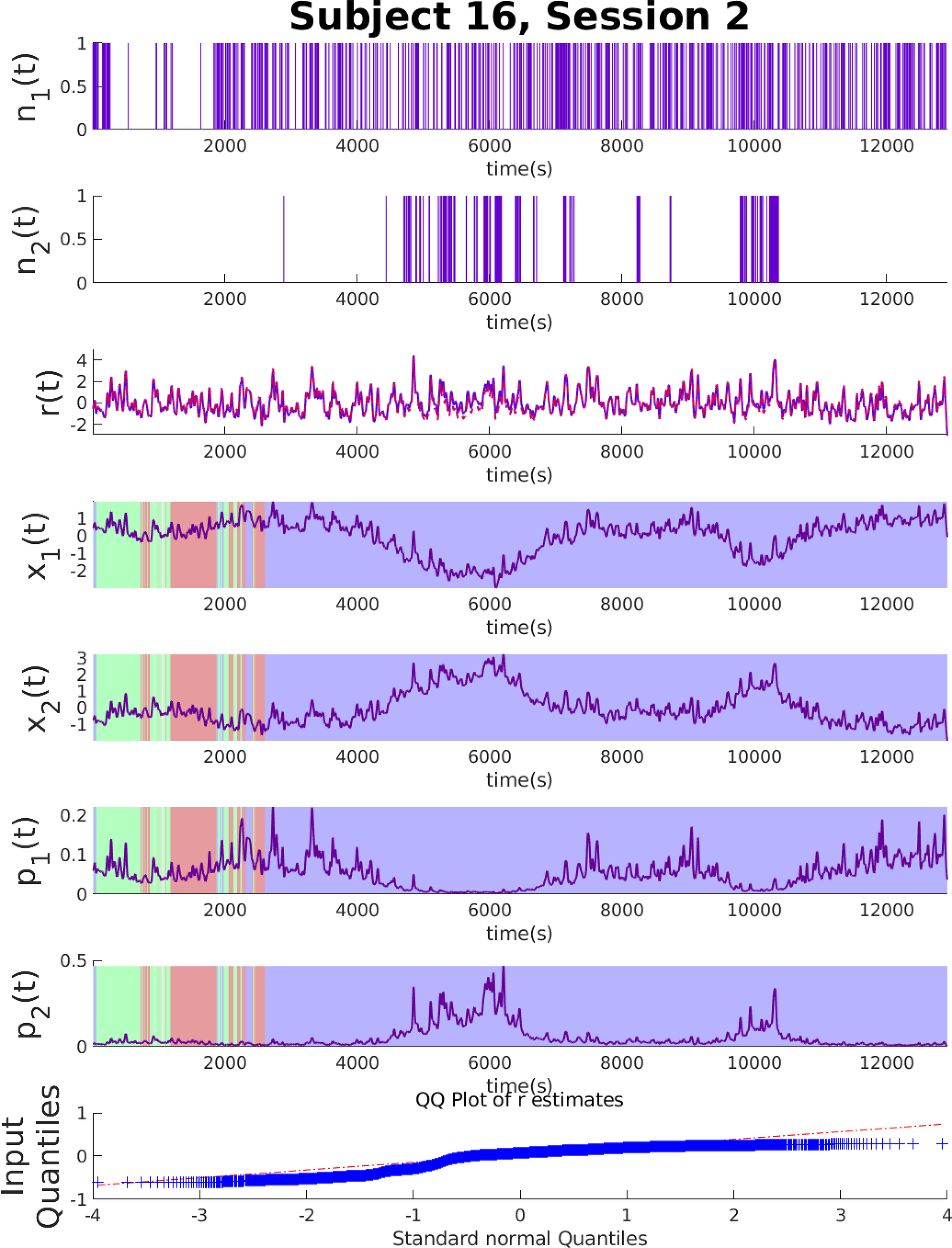

Supplement: S41 Fig — The panel shows the experimental data with time limit. From top, the binary variables n1 and n2 derived from deconvolved EDA data and typing data respectively, the continuous variable r denoting the RR intervals derived from heart rate (red line) and r˜ estimated from latent variables x1 and x2 (purple line), x1 and x2 in order from top indicating cognitive arousal state and expressive typing state respectively. p1 and p2 show the estimated probabilities. Patches of green, red, and cyan indicate what application the subject was using at the time of measurement. Green indicates applications for information search like internet explorer, red is for typing like Microsoft word and PowerPoint and cyan is for when subjects are looking at their emails. Finally, the QQ plot for the residual error of r is shown. (TIF) [file pone.0300786.s042.tif]

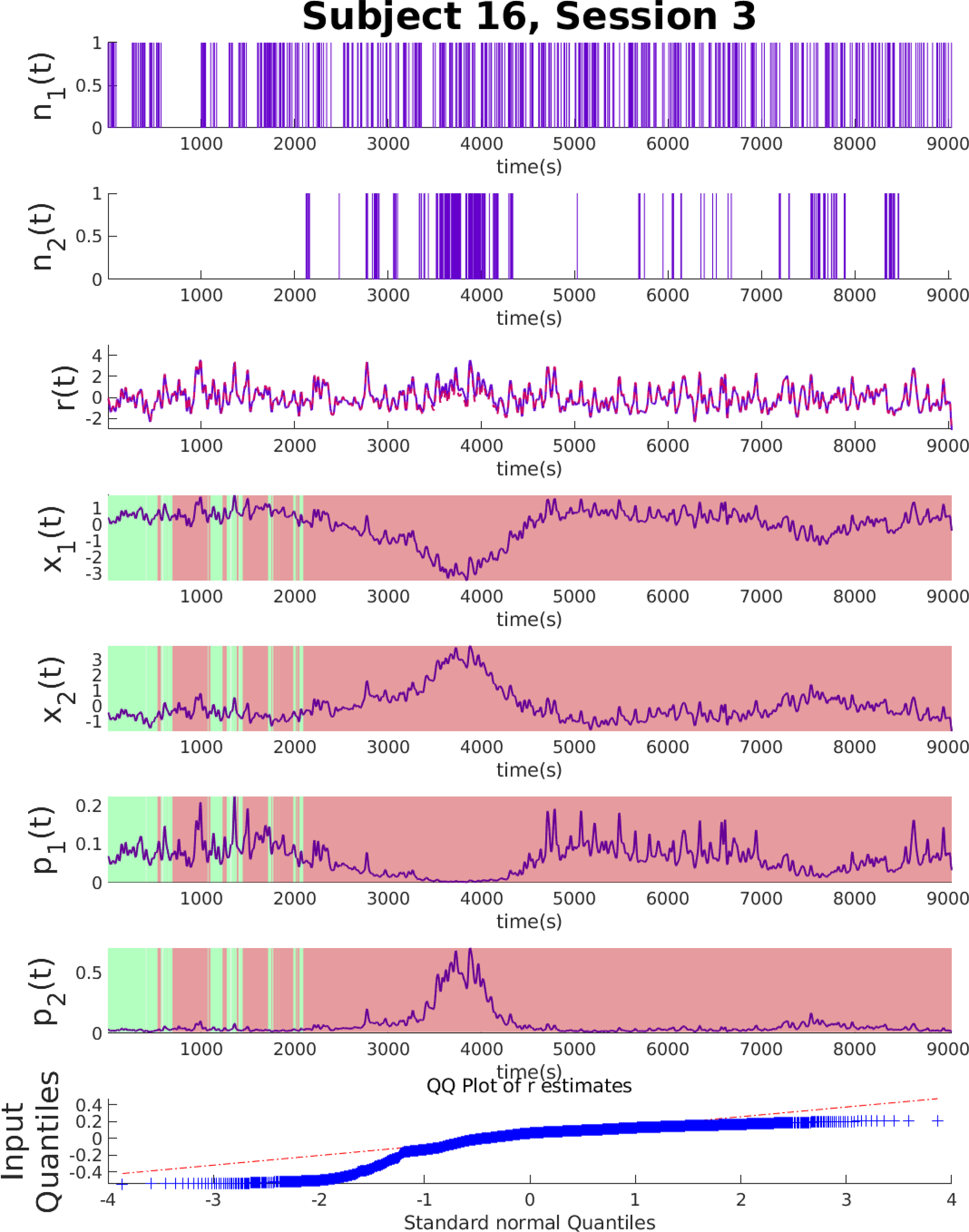

Supplement: S42 Fig — The panel shows the experimental data with interruptions. From top, the binary variables n1 and n2 derived from deconvolved EDA data and typing data respectively, the continuous variable r denoting the RR intervals derived from heart rate (red line) and r˜ estimated from latent variables x1 and x2 (purple line), x1 and x2 in order from top indicating cognitive arousal state and expressive typing state respectively. p1 and p2 show the estimated probabilities. Patches of green, red, and cyan indicate what application the subject was using at the time of measurement. Green indicates applications for information search like internet explorer, red is for typing like Microsoft word and PowerPoint and cyan is for when subjects are looking at their emails. The Blue vertical line indicates the time email notifications were sent. Finally, the QQ plot for the residual error of r is shown. (TIF) [file pone.0300786.s043.tif]

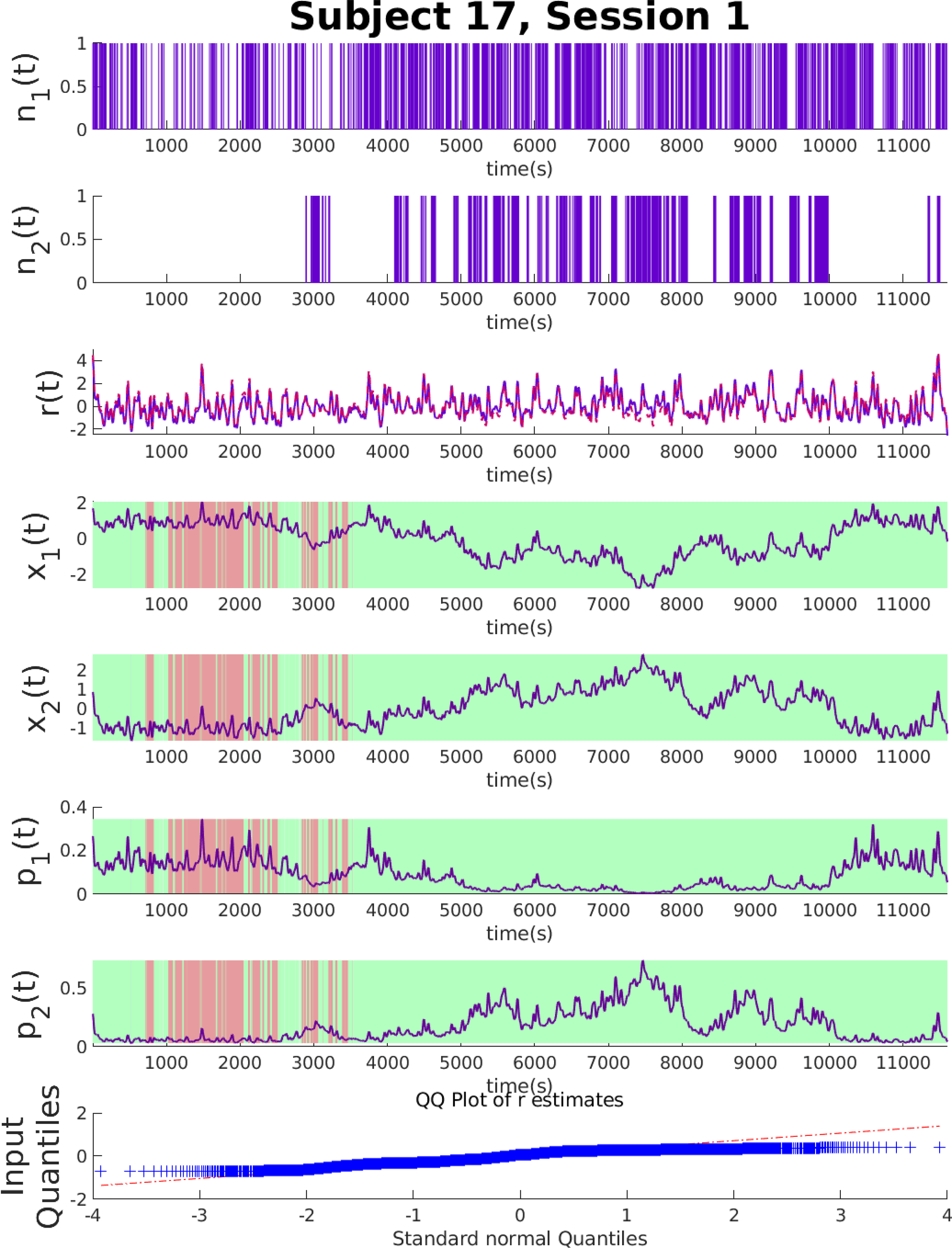

Supplement: S43 Fig — The panel shows the experimental data for no stressor sessions. From top, the binary variables n1 and n2 derived from deconvolved EDA data and typing data respectively, the continuous variable r denoting the RR intervals derived from heart rate (red line) and r˜ estimated from latent variables x1 and x2 (purple line), x1 and x2 in order from top indicating cognitive arousal state and expressive typing state respectively. p1 and p2 show the estimated probabilities. Patches of green, red, and cyan indicate what application the subject was using at the time of measurement. Green indicates applications for information search like internet explorer, red is for typing like Microsoft word and PowerPoint and cyan is for when subjects are looking at their emails. Finally, the QQ plot for the residual error of r is shown. (TIF) [file pone.0300786.s044.tif]

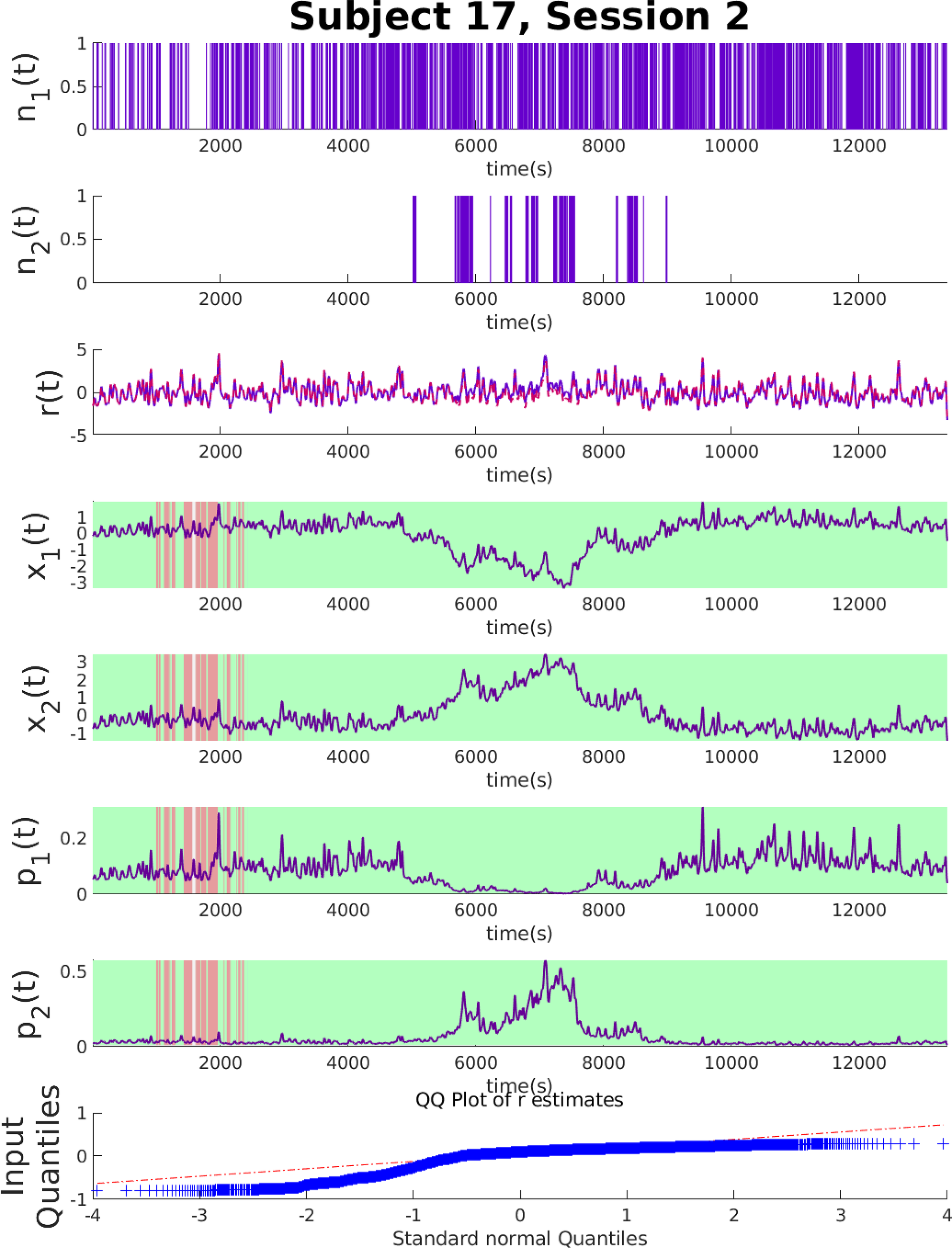

Supplement: S44 Fig — The panel shows the experimental data with time limit. From top, the binary variables n1 and n2 derived from deconvolved EDA data and typing data respectively, the continuous variable r denoting the RR intervals derived from heart rate (red line) and r˜ estimated from latent variables x1 and x2 (purple line), x1 and x2 in order from top indicating cognitive arousal state and expressive typing state respectively. p1 and p2 show the estimated probabilities. Patches of green, red, and cyan indicate what application the subject was using at the time of measurement. Green indicates applications for information search like internet explorer, red is for typing like Microsoft word and PowerPoint and cyan is for when subjects are looking at their emails. Finally, the QQ plot for the residual error of r is shown. (TIF) [file pone.0300786.s045.tif]

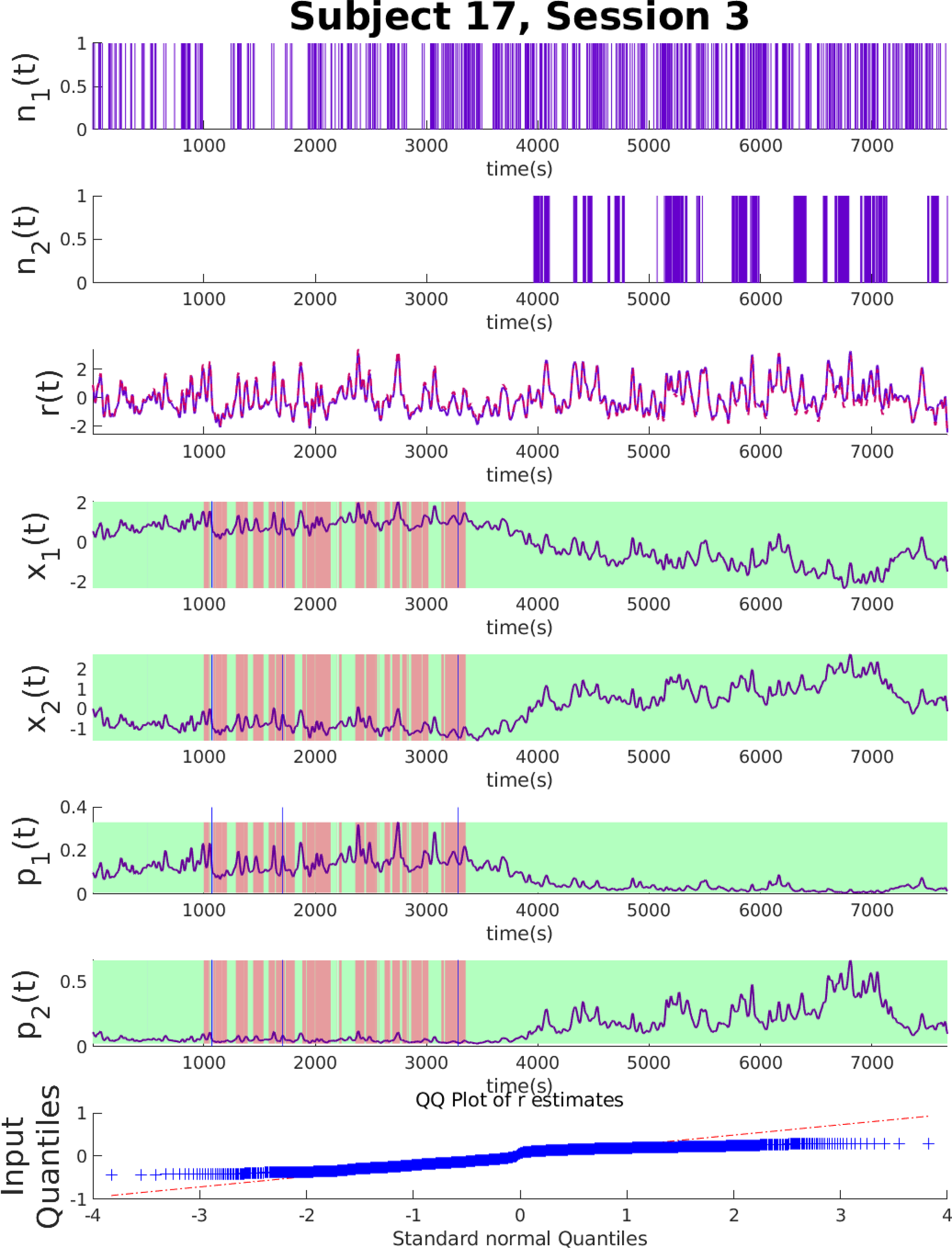

Supplement: S45 Fig — The panel shows the experimental data with interruptions. From top, the binary variables n1 and n2 derived from deconvolved EDA data and typing data respectively, the continuous variable r denoting the RR intervals derived from heart rate (red line) and r˜ estimated from latent variables x1 and x2 (purple line), x1 and x2 in order from top indicating cognitive arousal state and expressive typing state respectively. p1 and p2 show the estimated probabilities. Patches of green, red, and cyan indicate what application the subject was using at the time of measurement. Green indicates applications for information search like internet explorer, red is for typing like Microsoft word and PowerPoint and cyan is for when subjects are looking at their emails. The Blue vertical line indicates the time email notifications were sent. Finally, the QQ plot for the residual error of r is shown. (TIF) [file pone.0300786.s046.tif]

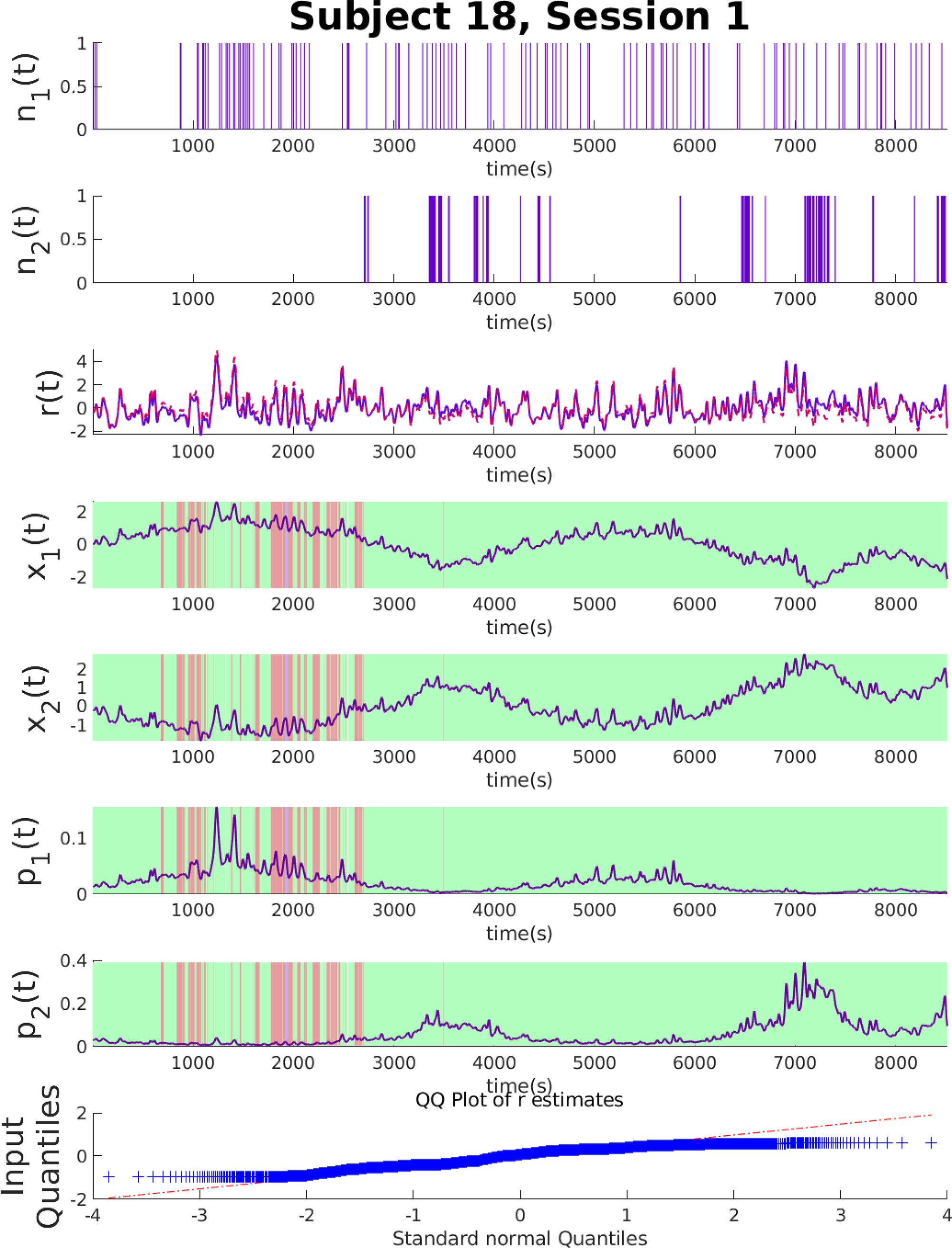

Supplement: S46 Fig — The panel shows the experimental data for no stressor sessions. From top, the binary variables n1 and n2 derived from deconvolved EDA data and typing data respectively, the continuous variable r denoting the RR intervals derived from heart rate (red line) and r˜ estimated from latent variables x1 and x2 (purple line), x1 and x2 in order from top indicating cognitive arousal state and expressive typing state respectively. p1 and p2 show the estimated probabilities. Patches of green, red, and cyan indicate what application the subject was using at the time of measurement. Green indicates applications for information search like internet explorer, red is for typing like Microsoft word and PowerPoint and cyan is for when subjects are looking at their emails. Finally, the QQ plot for the residual error of r is shown. (TIF) [file pone.0300786.s047.tif]

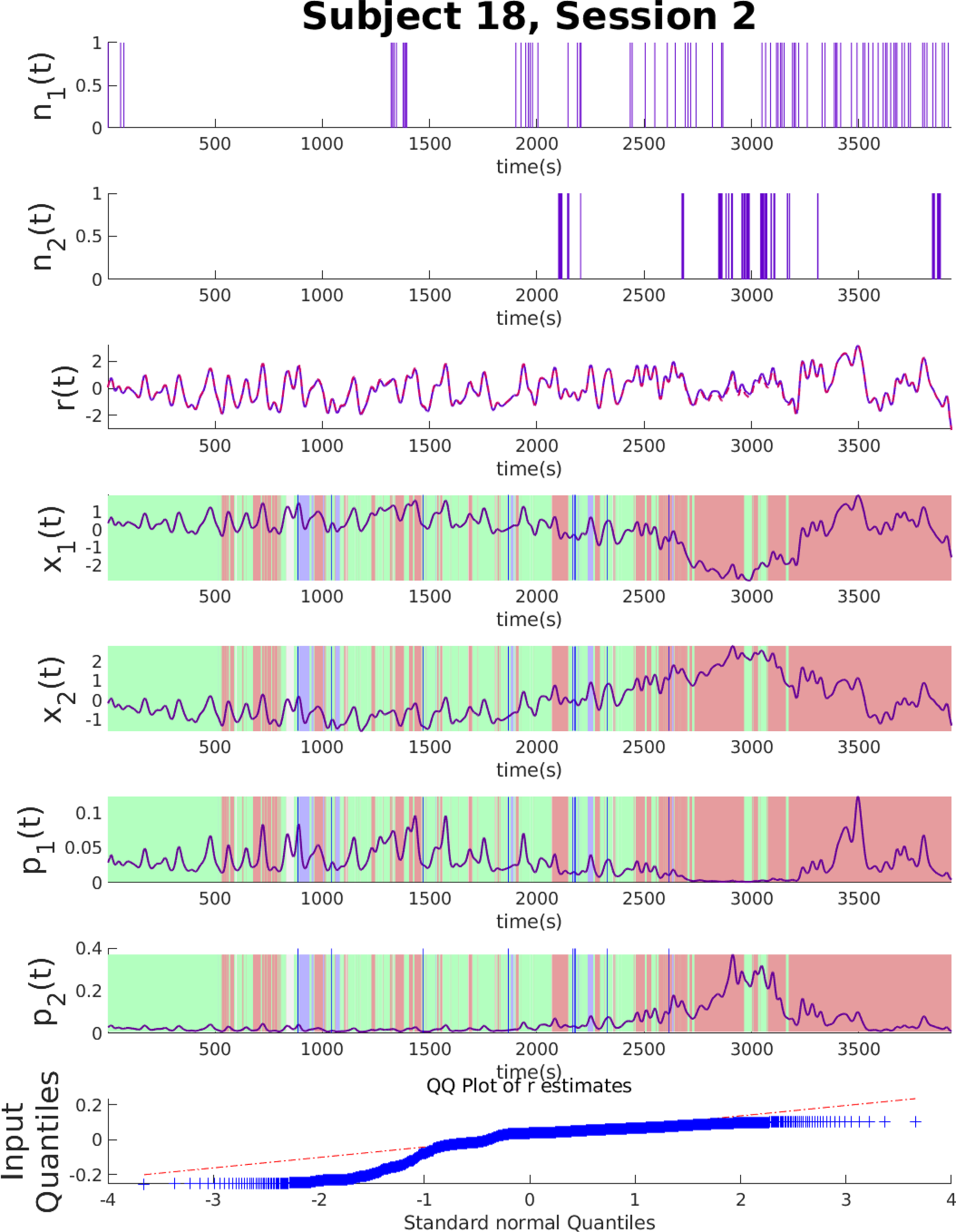

Supplement: S47 Fig — The panel shows the experimental data with time limit. From top, the binary variables n1 and n2 derived from deconvolved EDA data and typing data respectively, the continuous variable r denoting the RR intervals derived from heart rate (red line) and r˜ estimated from latent variables x1 and x2 (purple line), x1 and x2 in order from top indicating cognitive arousal state and expressive typing state respectively. p1 and p2 show the estimated probabilities. Patches of green, red, and cyan indicate what application the subject was using at the time of measurement. Green indicates applications for information search like internet explorer, red is for typing like Microsoft word and PowerPoint and cyan is for when subjects are looking at their emails. Finally, the QQ plot for the residual error of r is shown. (TIF) [file pone.0300786.s048.tif]

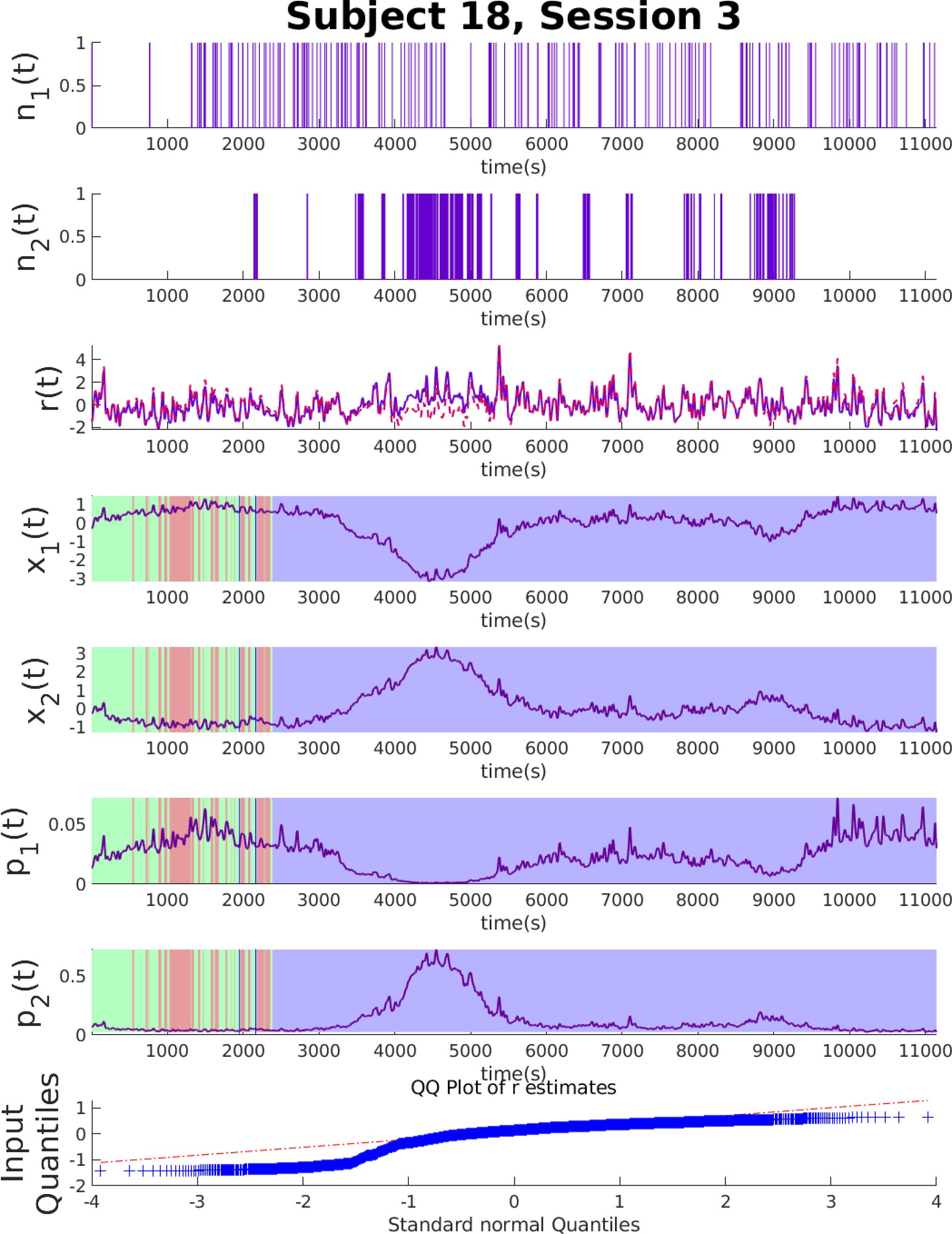

Supplement: S48 Fig — The panel shows the experimental data with interruptions. From top, the binary variables n1 and n2 derived from deconvolved EDA data and typing data respectively, the continuous variable r denoting the RR intervals derived from heart rate (red line) and r˜ estimated from latent variables x1 and x2 (purple line), x1 and x2 in order from top indicating cognitive arousal state and expressive typing state respectively. p1 and p2 show the estimated probabilities. Patches of green, red, and cyan indicate what application the subject was using at the time of measurement. Green indicates applications for information search like internet explorer, red is for typing like Microsoft word and PowerPoint and cyan is for when subjects are looking at their emails. The Blue vertical line indicates the time email notifications were sent. Finally, the QQ plot for the residual error of r is shown. (TIF) [file pone.0300786.s049.tif]

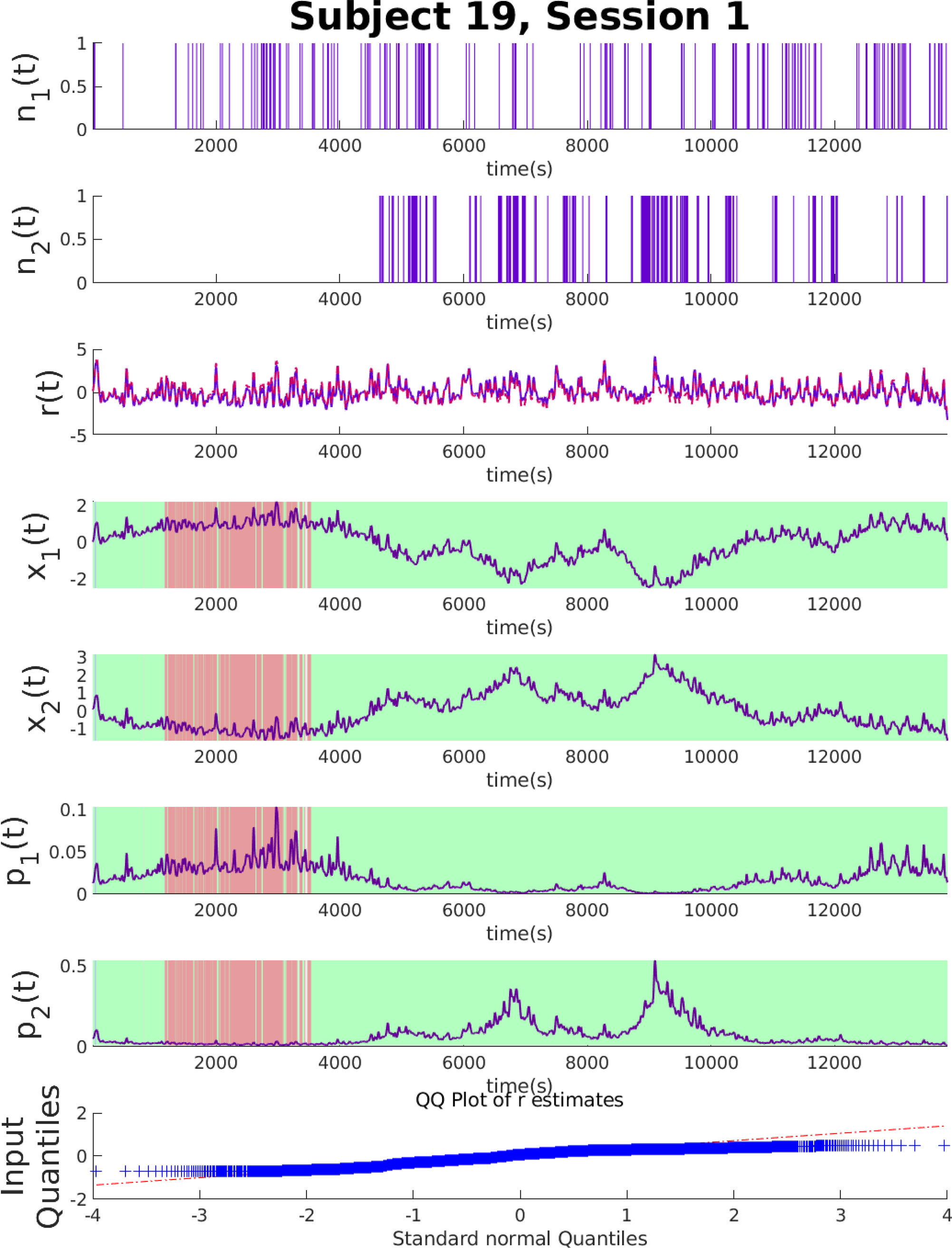

Supplement: S49 Fig — The panel shows the experimental data for no stressor sessions. From top, the binary variables n1 and n2 derived from deconvolved EDA data and typing data respectively, the continuous variable r denoting the RR intervals derived from heart rate (red line) and r˜ estimated from latent variables x1 and x2 (purple line), x1 and x2 in order from top indicating cognitive arousal state and expressive typing state respectively. p1 and p2 show the estimated probabilities. Patches of green, red, and cyan indicate what application the subject was using at the time of measurement. Green indicates applications for information search like internet explorer, red is for typing like Microsoft word and PowerPoint and cyan is for when subjects are looking at their emails. Finally, the QQ plot for the residual error of r is shown. (TIF) [file pone.0300786.s050.tif]

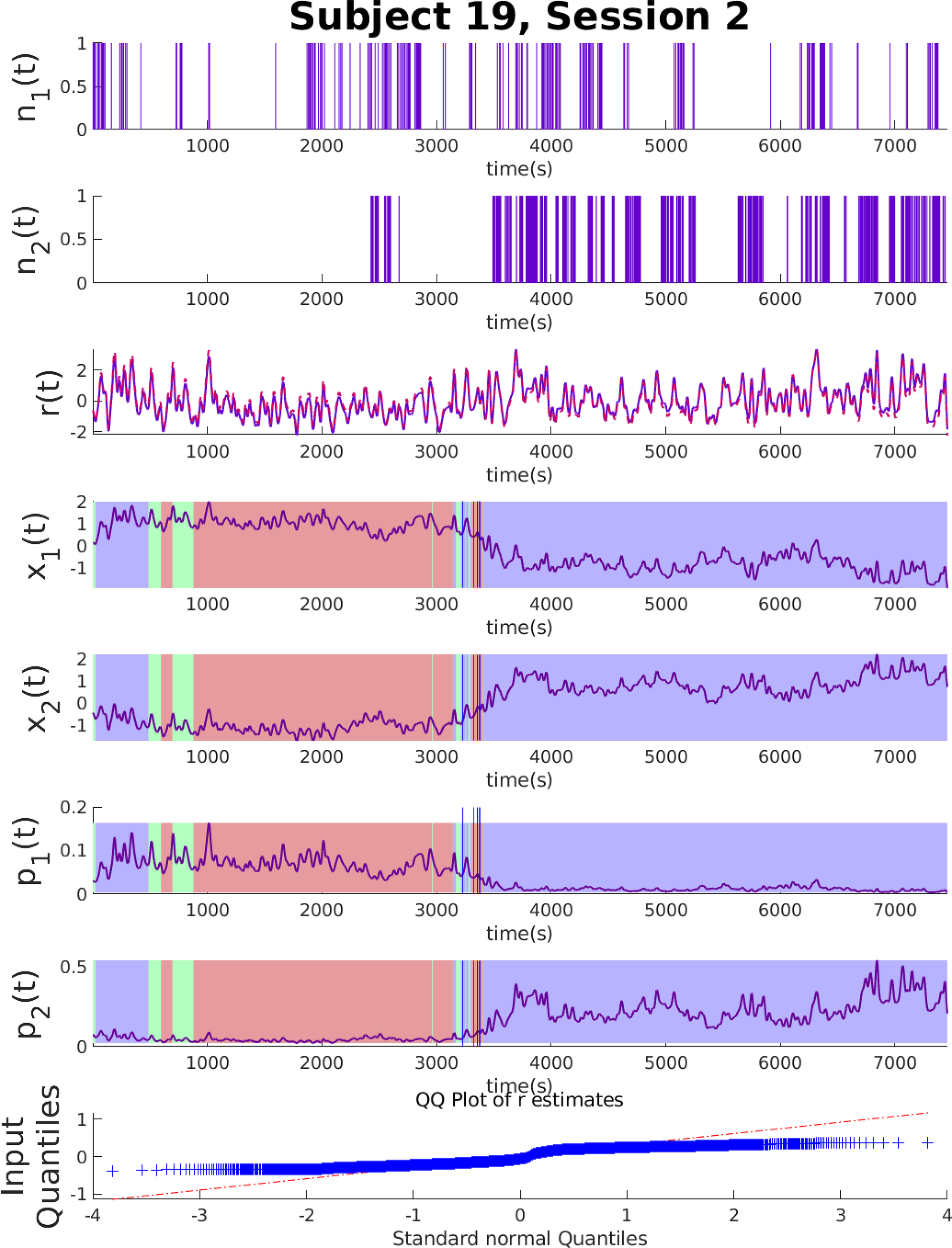

Supplement: S50 Fig — The panel shows the experimental data with time limit. From top, the binary variables n1 and n2 derived from deconvolved EDA data and typing data respectively, the continuous variable r denoting the RR intervals derived from heart rate (red line) and r˜ estimated from latent variables x1 and x2 (purple line), x1 and x2 in order from top indicating cognitive arousal state and expressive typing state respectively. p1 and p2 show the estimated probabilities. Patches of green, red, and cyan indicate what application the subject was using at the time of measurement. Green indicates applications for information search like internet explorer, red is for typing like Microsoft word and PowerPoint and cyan is for when subjects are looking at their emails. Finally, the QQ plot for the residual error of r is shown. (TIF) [file pone.0300786.s051.tif]

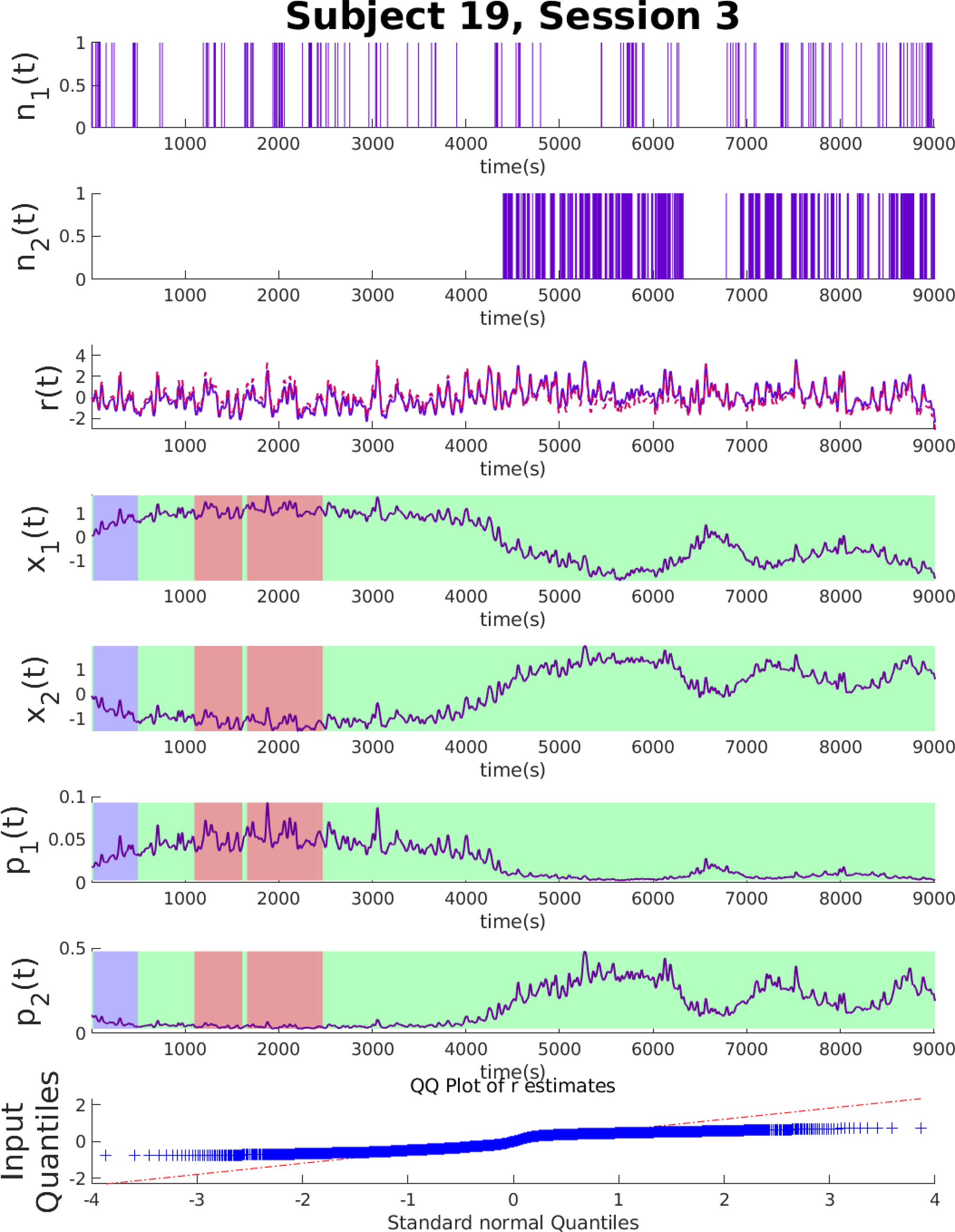

Supplement: S51 Fig — The panel shows the experimental data with interruptions. From top, the binary variables n1 and n2 derived from deconvolved EDA data and typing data respectively, the continuous variable r denoting the RR intervals derived from heart rate (red line) and r˜ estimated from latent variables x1 and x2 (purple line), x1 and x2 in order from top indicating cognitive arousal state and expressive typing state respectively. p1 and p2 show the estimated probabilities. Patches of green, red, and cyan indicate what application the subject was using at the time of measurement. Green indicates applications for information search like internet explorer, red is for typing like Microsoft word and PowerPoint and cyan is for when subjects are looking at their emails. The Blue vertical line indicates the time email notifications were sent. Finally, the QQ plot for the residual error of r is shown. (TIF) [file pone.0300786.s052.tif]

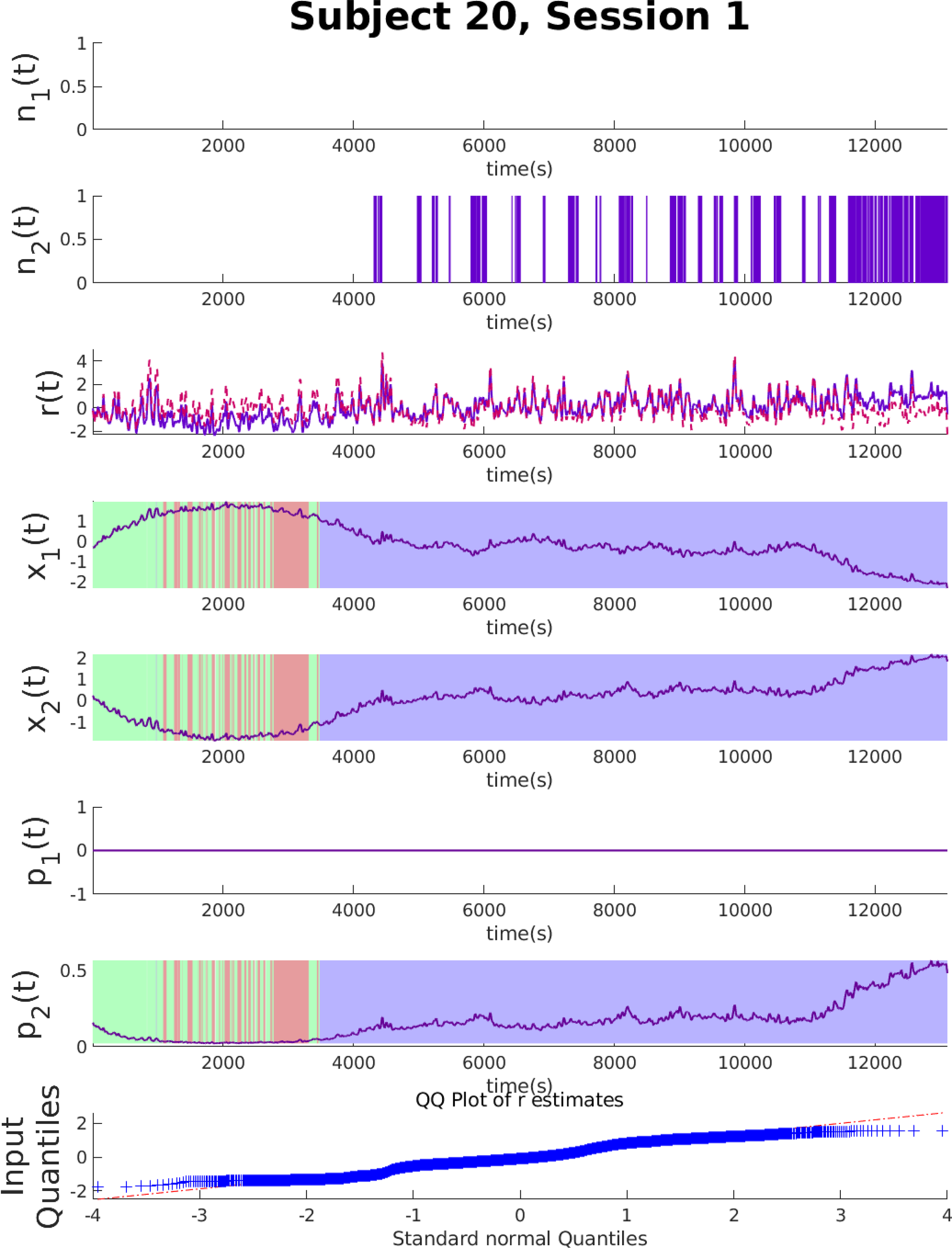

Supplement: S52 Fig — The panel shows the experimental data for no stressor sessions. From top, the binary variables n1 and n2 derived from deconvolved EDA data and typing data respectively, the continuous variable r denoting the RR intervals derived from heart rate (red line) and r˜ estimated from latent variables x1 and x2 (purple line), x1 and x2 in order from top indicating cognitive arousal state and expressive typing state respectively. p1 and p2 show the estimated probabilities. Patches of green, red, and cyan indicate what application the subject was using at the time of measurement. Green indicates applications for information search like internet explorer, red is for typing like Microsoft word and PowerPoint and cyan is for when subjects are looking at their emails. Finally, the QQ plot for the residual error of r is shown. (TIF) [file pone.0300786.s053.tif]

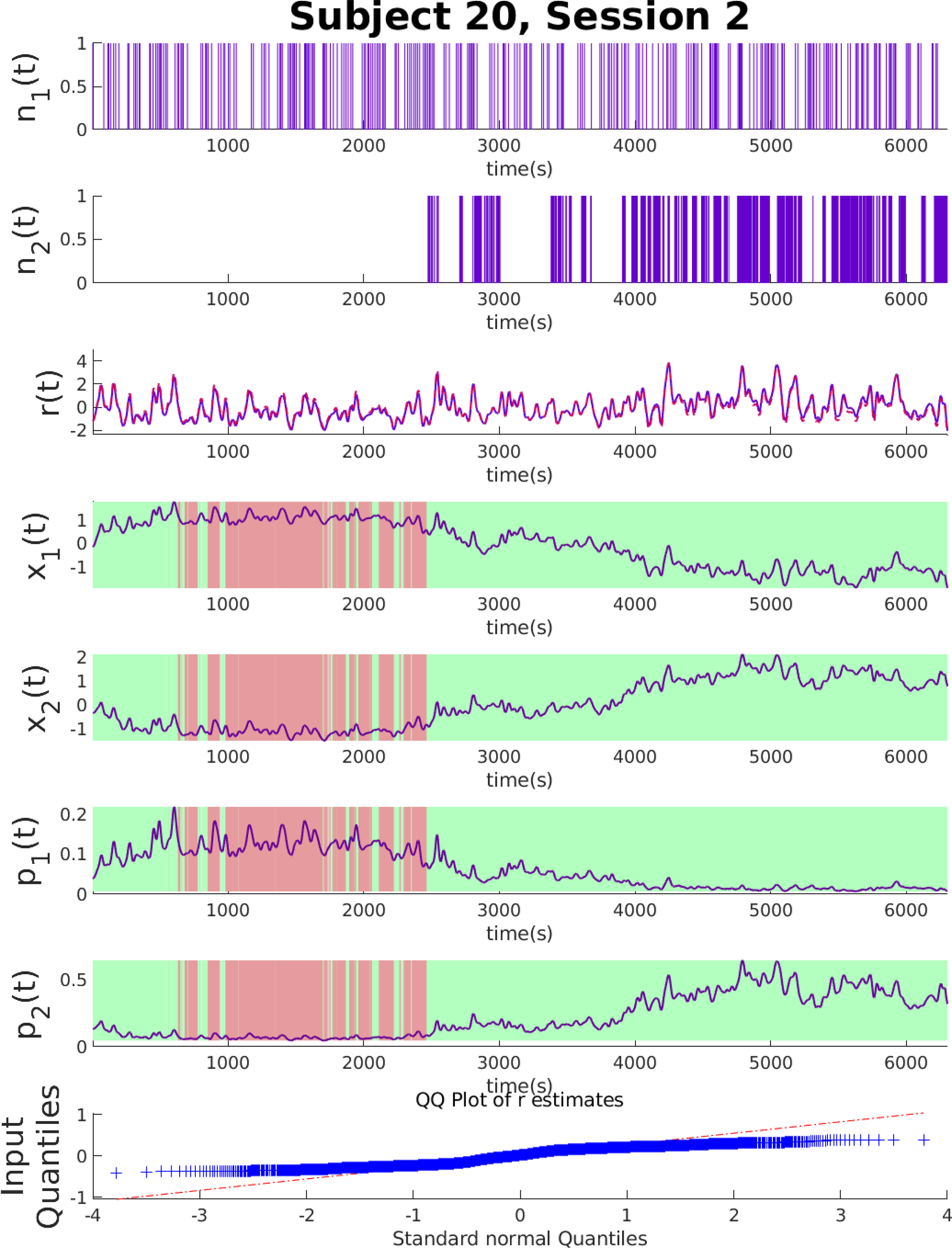

Supplement: S53 Fig — The panel shows the experimental data with time limit. From top, the binary variables n1 and n2 derived from deconvolved EDA data and typing data respectively, the continuous variable r denoting the RR intervals derived from heart rate (red line) and r˜ estimated from latent variables x1 and x2 (purple line), x1 and x2 in order from top indicating cognitive arousal state and expressive typing state respectively. p1 and p2 show the estimated probabilities. Patches of green, red, and cyan indicate what application the subject was using at the time of measurement. Green indicates applications for information search like internet explorer, red is for typing like Microsoft word and PowerPoint and cyan is for when subjects are looking at their emails. Finally, the QQ plot for the residual error of r is shown. (TIF) [file pone.0300786.s054.tif]

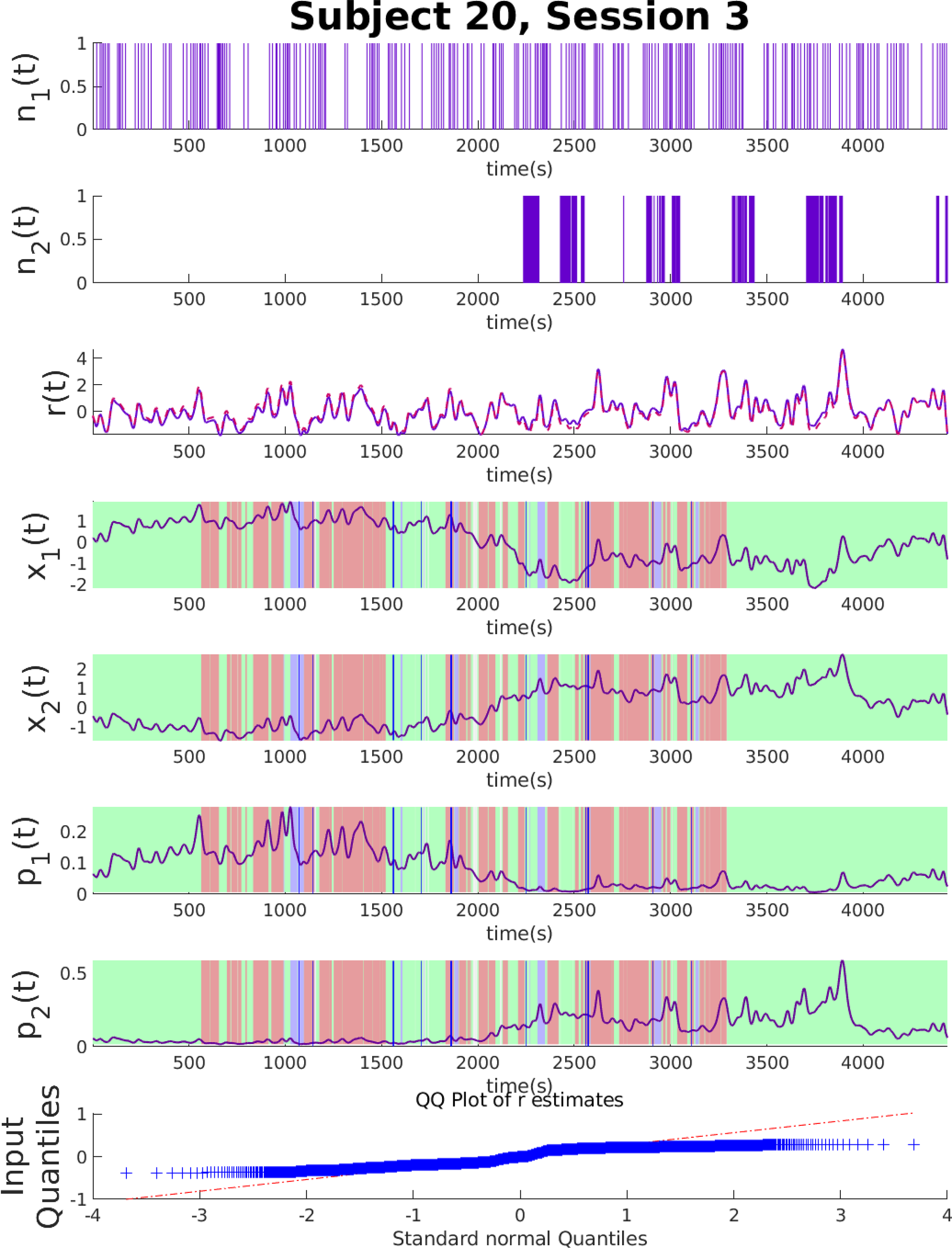

Supplement: S54 Fig — The panel shows the experimental data with interruptions. From top, the binary variables n1 and n2 derived from deconvolved EDA data and typing data respectively, the continuous variable r denoting the RR intervals derived from heart rate (red line) and r˜ estimated from latent variables x1 and x2 (purple line), x1 and x2 in order from top indicating cognitive arousal state and expressive typing state respectively. p1 and p2 show the estimated probabilities. Patches of green, red, and cyan indicate what application the subject was using at the time of measurement. Green indicates applications for information search like internet explorer, red is for typing like Microsoft word and PowerPoint and cyan is for when subjects are looking at their emails. The Blue vertical line indicates the time email notifications were sent. Finally, the QQ plot for the residual error of r is shown. (TIF) [file pone.0300786.s055.tif]

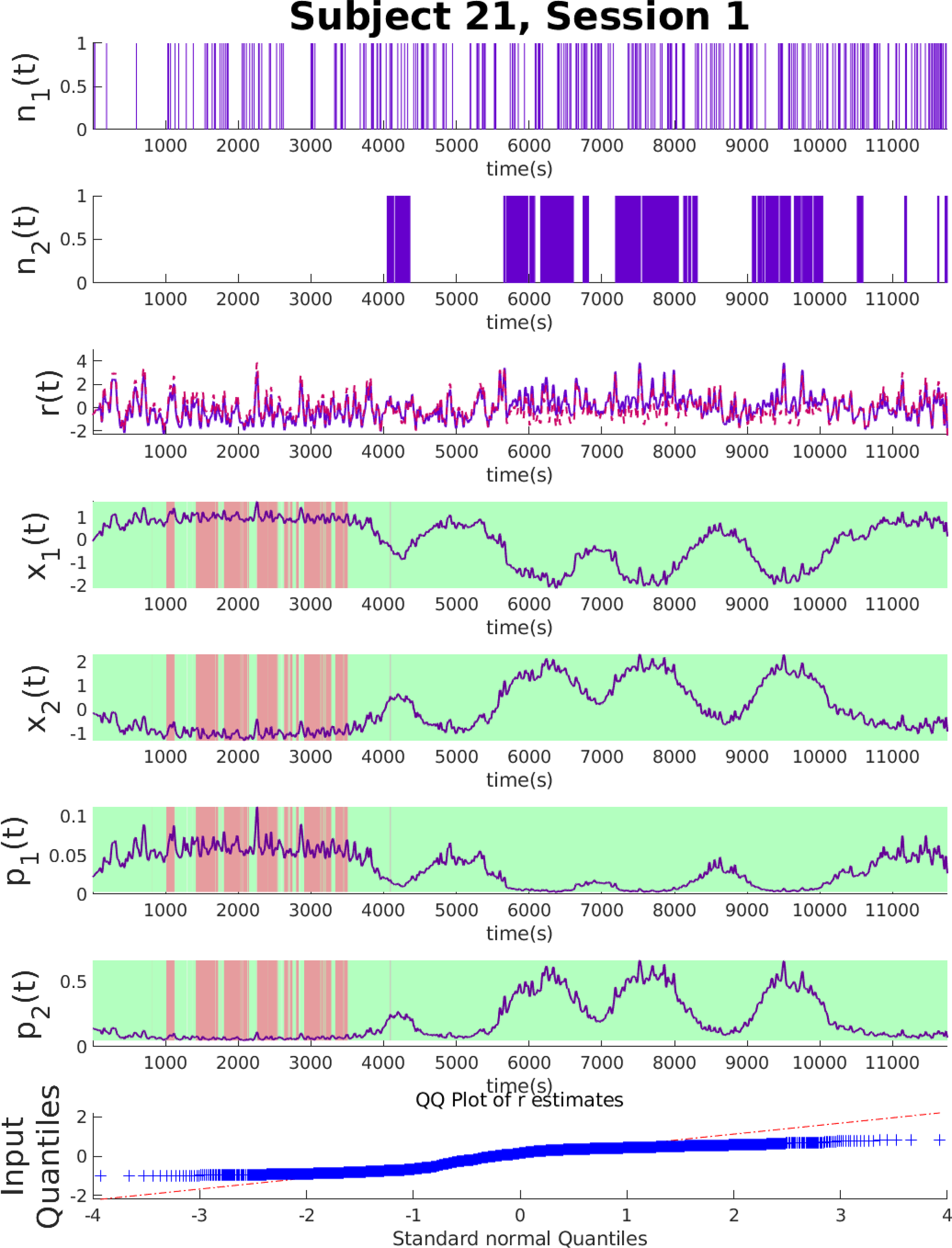

Supplement: S55 Fig — The panel shows the experimental data for no stressor sessions. From top, the binary variables n1 and n2 derived from deconvolved EDA data and typing data respectively, the continuous variable r denoting the RR intervals derived from heart rate (red line) and r˜ estimated from latent variables x1 and x2 (purple line), x1 and x2 in order from top indicating cognitive arousal state and expressive typing state respectively. p1 and p2 show the estimated probabilities. Patches of green, red, and cyan indicate what application the subject was using at the time of measurement. Green indicates applications for information search like internet explorer, red is for typing like Microsoft word and PowerPoint and cyan is for when subjects are looking at their emails. Finally, the QQ plot for the residual error of r is shown. (TIF) [file pone.0300786.s056.tif]

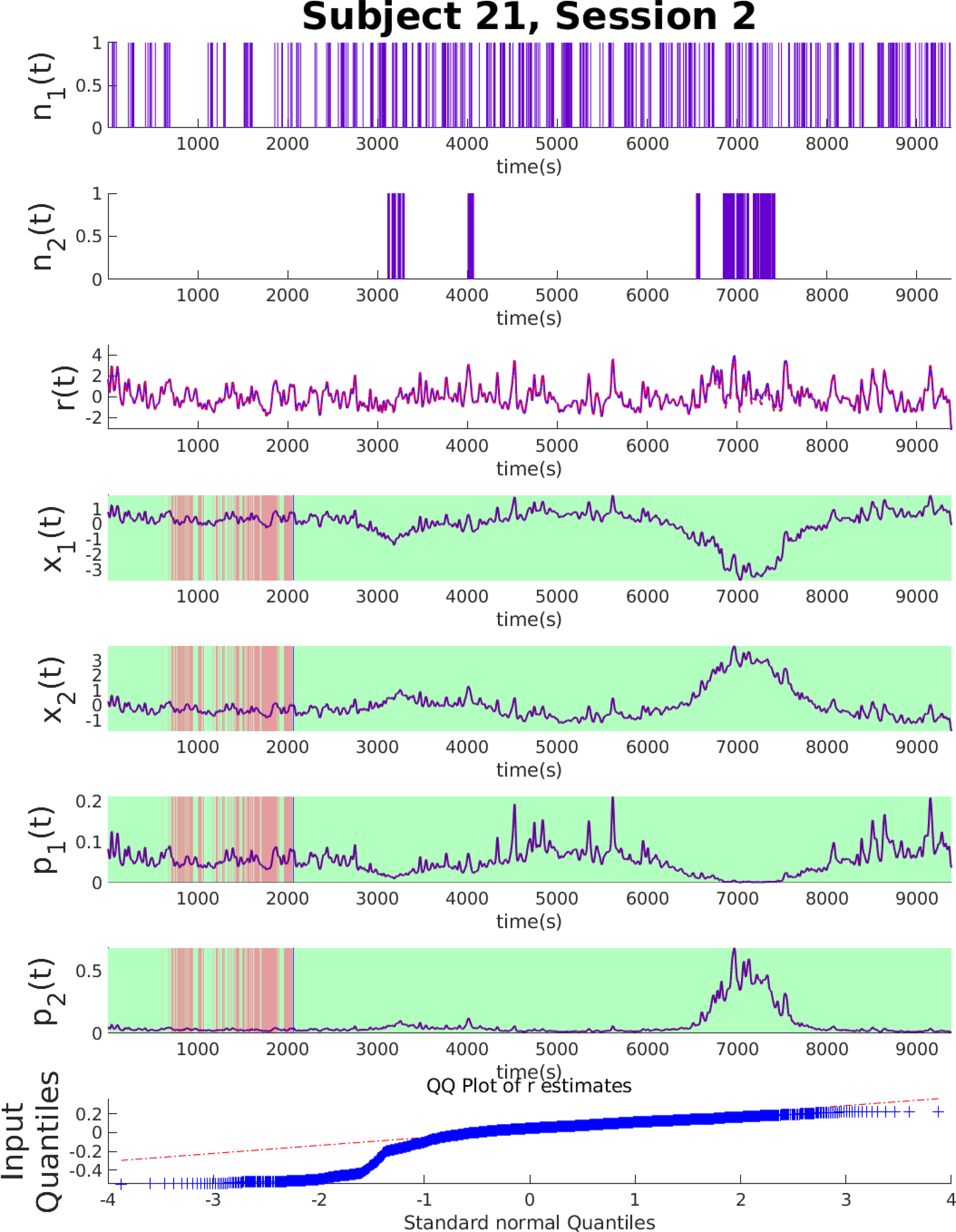

Supplement: S56 Fig — The panel shows the experimental data with time limit. From top, the binary variables n1 and n2 derived from deconvolved EDA data and typing data respectively, the continuous variable r denoting the RR intervals derived from heart rate (red line) and r˜ estimated from latent variables x1 and x2 (purple line), x1 and x2 in order from top indicating cognitive arousal state and expressive typing state respectively. p1 and p2 show the estimated probabilities. Patches of green, red, and cyan indicate what application the subject was using at the time of measurement. Green indicates applications for information search like internet explorer, red is for typing like Microsoft word and PowerPoint and cyan is for when subjects are looking at their emails. Finally, the QQ plot for the residual error of r is shown. (TIF) [file pone.0300786.s057.tif]

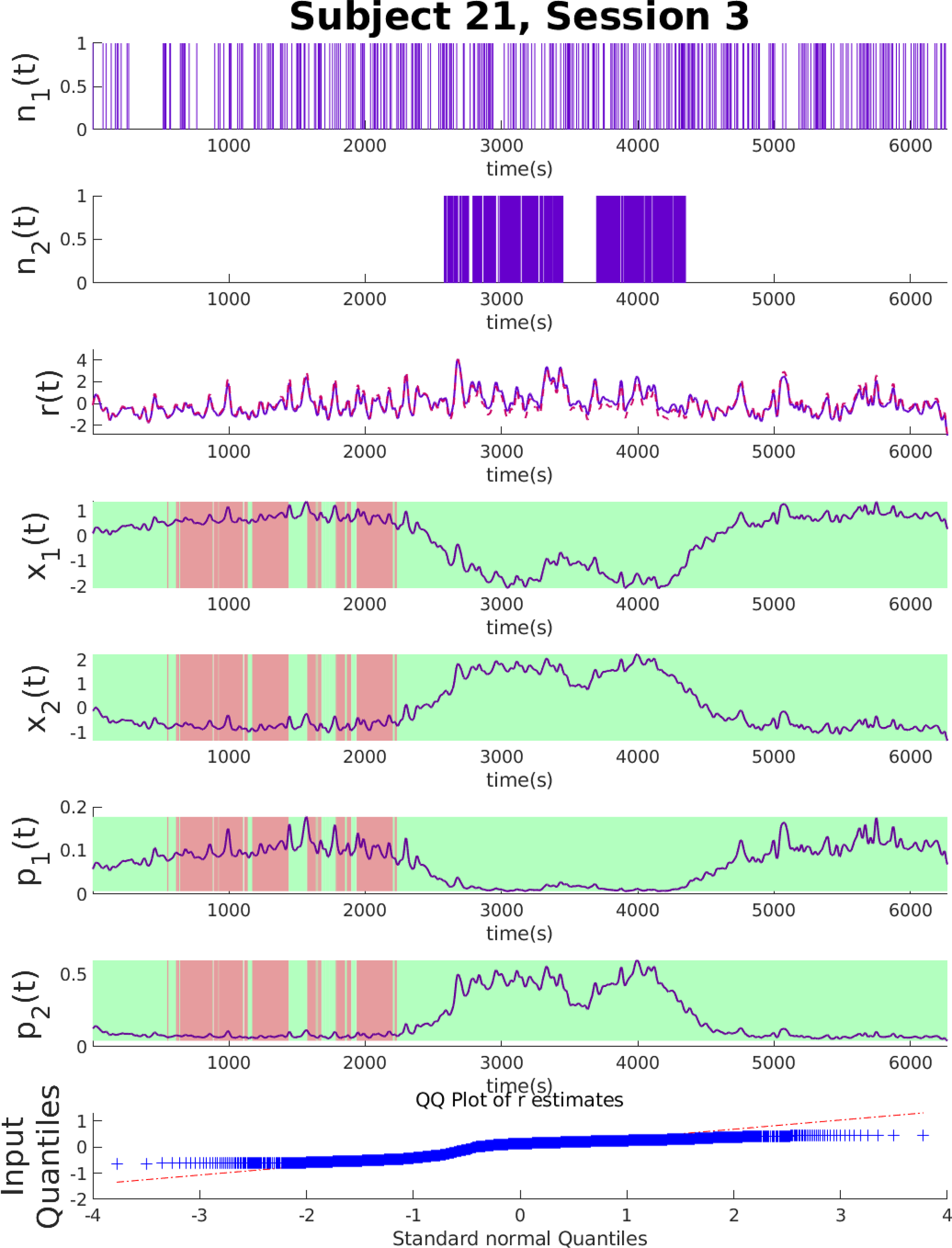

Supplement: S57 Fig — The panel shows the experimental data with interruptions. From top, the binary variables n1 and n2 derived from deconvolved EDA data and typing data respectively, the continuous variable r denoting the RR intervals derived from heart rate (red line) and r˜ estimated from latent variables x1 and x2 (purple line), x1 and x2 in order from top indicating cognitive arousal state and expressive typing state respectively. p1 and p2 show the estimated probabilities. Patches of green, red, and cyan indicate what application the subject was using at the time of measurement. Green indicates applications for information search like internet explorer, red is for typing like Microsoft word and PowerPoint and cyan is for when subjects are looking at their emails. The Blue vertical line indicates the time email notifications were sent. Finally, the QQ plot for the residual error of r is shown. (TIF) [file pone.0300786.s058.tif]

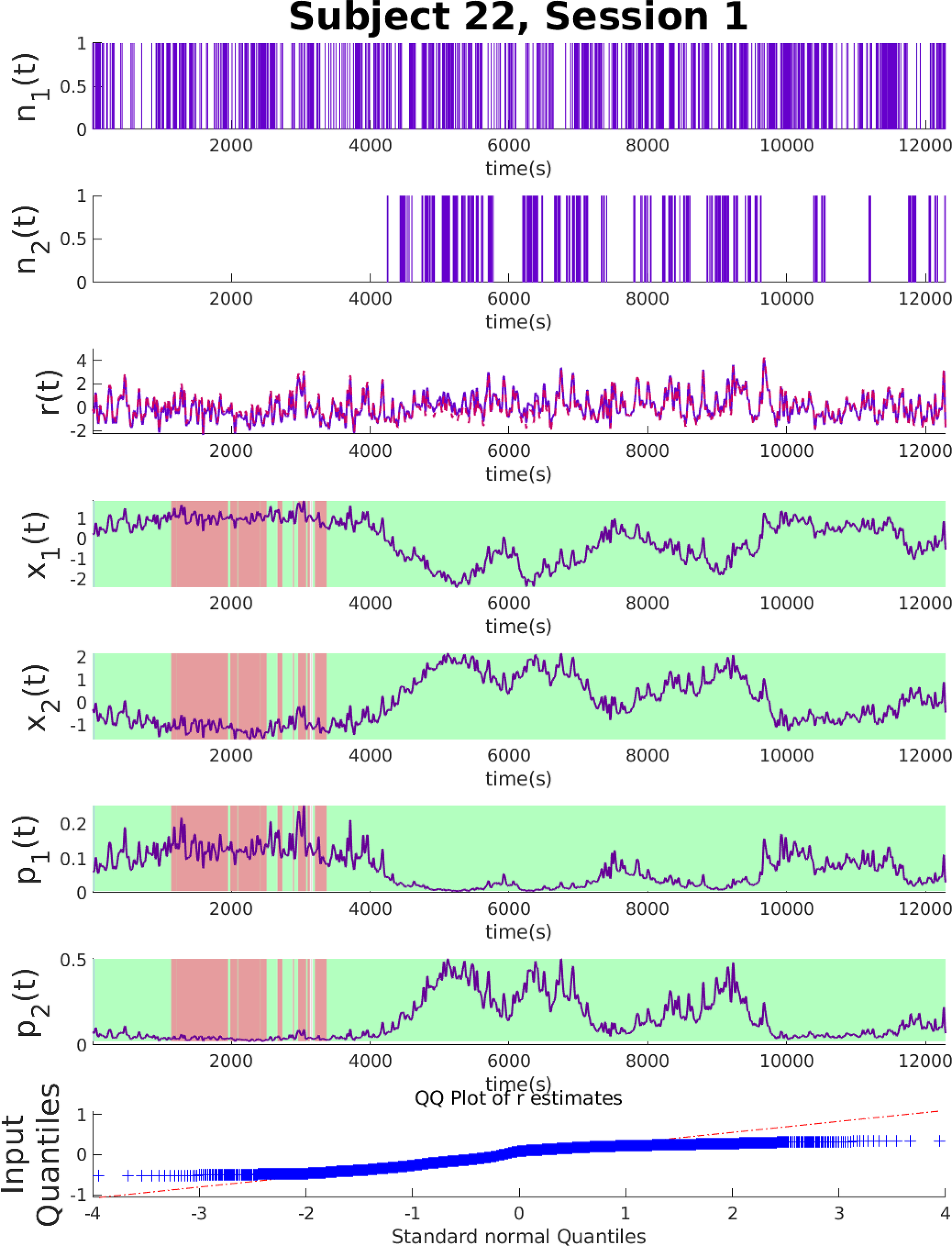

Supplement: S58 Fig — The panel shows the experimental data for no stressor sessions. From top, the binary variables n1 and n2 derived from deconvolved EDA data and typing data respectively, the continuous variable r denoting the RR intervals derived from heart rate (red line) and r˜ estimated from latent variables x1 and x2 (purple line), x1 and x2 in order from top indicating cognitive arousal state and expressive typing state respectively. p1 and p2 show the estimated probabilities. Patches of green, red, and cyan indicate what application the subject was using at the time of measurement. Green indicates applications for information search like internet explorer, red is for typing like Microsoft word and PowerPoint and cyan is for when subjects are looking at their emails. Finally, the QQ plot for the residual error of r is shown. (TIF) [file pone.0300786.s059.tif]

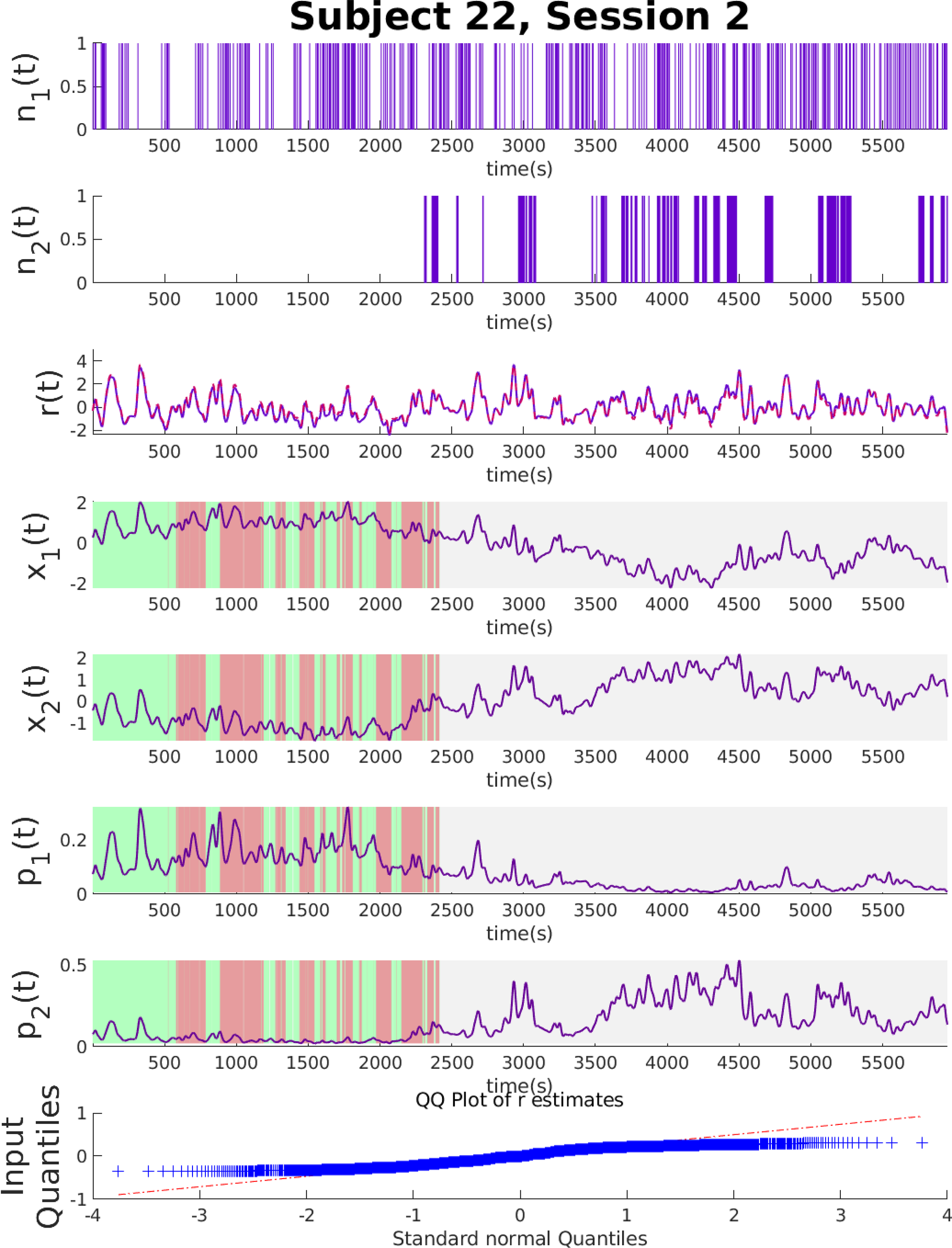

Supplement: S59 Fig — The panel shows the experimental data with time limit. From top, the binary variables n1 and n2 derived from deconvolved EDA data and typing data respectively, the continuous variable r denoting the RR intervals derived from heart rate (red line) and r˜ estimated from latent variables x1 and x2 (purple line), x1 and x2 in order from top indicating cognitive arousal state and expressive typing state respectively. p1 and p2 show the estimated probabilities. Patches of green, red, and cyan indicate what application the subject was using at the time of measurement. Green indicates applications for information search like internet explorer, red is for typing like Microsoft word and PowerPoint and cyan is for when subjects are looking at their emails. Finally, the QQ plot for the residual error of r is shown. (TIF) [file pone.0300786.s060.tif]

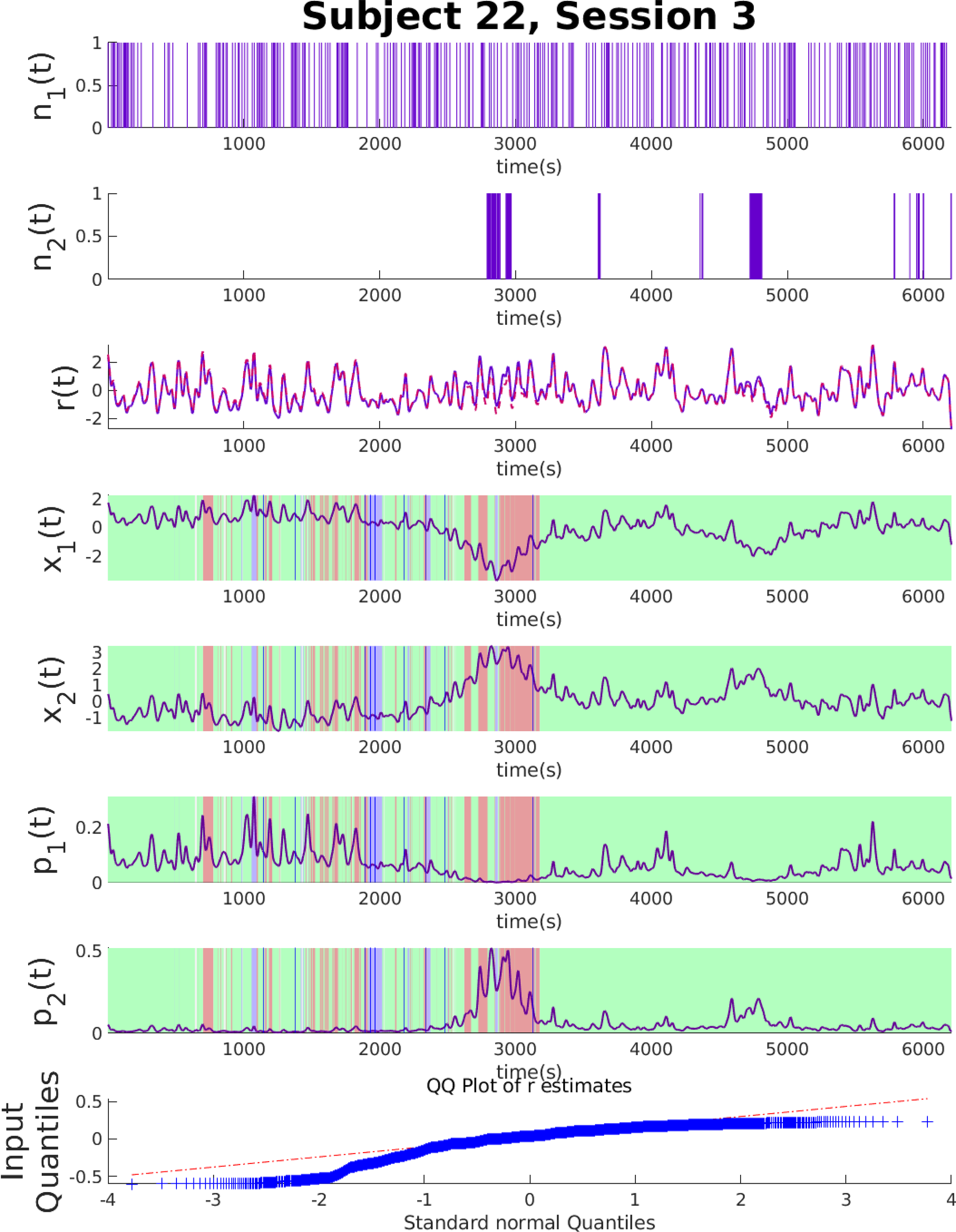

Supplement: S60 Fig — The panel shows the experimental data with interruptions. From top, the binary variables n1 and n2 derived from deconvolved EDA data and typing data respectively, the continuous variable r denoting the RR intervals derived from heart rate (red line) and r˜ estimated from latent variables x1 and x2 (purple line), x1 and x2 in order from top indicating cognitive arousal state and expressive typing state respectively. p1 and p2 show the estimated probabilities. Patches of green, red, and cyan indicate what application the subject was using at the time of measurement. Green indicates applications for information search like internet explorer, red is for typing like Microsoft word and PowerPoint and cyan is for when subjects are looking at their emails. The Blue vertical line indicates the time email notifications were sent. Finally, the QQ plot for the residual error of r is shown. (TIF) [file pone.0300786.s061.tif]
